# A putative autonomous 20.5 kb-CACTA transposon insertion in an *F3'H *allele identifies a new CACTA transposon subfamily in *Glycine max*

**DOI:** 10.1186/1471-2229-8-124

**Published:** 2008-12-02

**Authors:** Gracia Zabala, Lila Vodkin

**Affiliations:** 1 Department of Crop Sciences, University of Illinois, Urbana, Illinois 61801, USA

## Abstract

**Background:**

The molecular organization of very few genetically defined CACTA transposon systems have been characterized thoroughly as those of *Spm/En *in maize, *Tam1 *of *Antirrhinum majus Candystripe1 *(*Cs1*) from *Sorghum bicolor *and *CAC1 *from *Arabidopsis thaliana*, for example. To date, only defective deletion derivatives of CACTA elements have been described for soybean, an economically important plant species whose genome sequence will be completed in 2008.

**Results:**

We identified a 20.5 kb insertion in a soybean flavonoid 3'-hydroxylase (*F3'H*) gene representing the t* allele (stable gray trichome color) whose origin traces to a single mutable chimeric plant displaying both tawny and gray trichomes. This 20.5 kb insertion has the molecular structure of a putative autonomous transposon of the CACTA family, designated Tgmt*. It encodes a large gene that was expressed in two sister isolines (T* and *t*^*m*^) of the stable gray line (t*) from which Tgmt* was isolated. RT-PCR derived cDNAs uncovered the structure of a large precursor mRNA as well as alternatively spliced transcripts reminiscent of the TNPA-mRNA generated by the *En-1 *element of maize but without sequence similarity to the maize TNPA. The larger mRNA encodes a transposase with a tnp2 and TNP1-transposase family domains. Because the two soybean lines expressing Tgmt* were derived from the same mutable chimeric plant that created the stable gray trichome t* allele line from which the element was isolated, Tgmt* has the potential to be an autonomous element that was rapidly inactivated in the stable gray trichome t* line. Comparison of Tgmt* to previously described *Tgm *elements demonstrated that two subtypes of CACTA transposon families exist in soybean based on divergence of their characteristic subterminal repeated motifs and their transposases. In addition, we report the sequence and annotation of a BAC clone containing the *F3'H *gene (*T *locus) which was interrupted by the novel Tgmt* element in the gray trichome allele t*.

**Conclusion:**

The molecular characterization of a 20.5 kb insertion in the flavonoid 3'-hydroxylase (*F3'H*) gene of a soybean gray pubescence allele (t*) identified the structure of a CACTA transposon designated Tgmt*. Besides the terminal inverted repeats and subterminal repeated motifs,Tgmt* encoded a large gene with two putative functions that are required for excision and transposition of a CACTA element, a transposase and the DNA binding protein known to associate to the subterminal repeated motifs. The degree of dissimilarity between Tgmt* transposase and subterminal repeated motifs with those of previously characterized defective CACTA elements (*Tgm1-7*) were evidence of the existence of two subfamilies of CACTA transposons in soybean, an observation not previously reported in other plants. In addition, our analyses of a genetically active and potentially autonomous element sheds light on the complete structure of a soybean element that is useful for annotation of the repetitive fraction of the soybean genome sequence and may prove useful for transposon tagging or transposon display experiments in different genetic lines.

## Background

The CACTA transposons are characterized by terminal inverted repeats (TIRs), target-site duplications of conserved length and transposition through a DNA intermediate. One of the best genetically characterized transposons is the *Suppressor-Mutator *(*Spm*) also named *Enhancer *(*En*) system of *Z. mays *[1,2]. It consists of two components, the *Spm/En *element that is autonomous with regard to excision, transposition and integration. The defective *Spm *(*dSpm*) or Inhibitor (*I*) elements are non-autonomous, generally internal deletion derivatives, that transpose only if an active *Spm/En *is present elsewhere in the genome to provide the *trans*-active Suppressor and Mutator functions of the element [3].

The *Spm/En *element was molecularly characterized [4,5] and shown to be 8.3 kb with capacity to encode at least two alternatively spliced transcripts of 5.8 kb and 2.5 kb. The 2.5 kb transcript was 100 times more abundant than the 5.8 kb mRNA and contains 11 exons of the *tnpA *gene. In addition to the multiple exons of the tnpA gene, the 5.8 kb *tnpD *transcript (molecularly known as the *tnp2 *region in similar elements in other species) contains the large orf from the Intron-1 region at the 5'-end of the element. The larger *tnpD *transcript (with a *tnp2 *domain) encoded a putative transposase required for the excision/integration in a transposition event whereas *tnpA *codes for a putative protein of 67 kd that functions as a DNA binding protein [6,7]. The TNPA protein recognizes the subterminal repeats, which act as *cis*-determinants for the excision and transposition of the *Spm/En *element [8]. The Suppressor function of *Spm/En *was also assigned to the TNPA protein [9]. The binding of TNPA to subterminal domains of defective *Spm/En *elements has been shown to create a steric block to the advancement of RNA polymerase, resulting in transcripts terminated prematurely [10]. Both proteins, TNPD and TNPA, are absolutely required for transposition of *Spm/En *[11,12]. The proteins may be provided *in trans *from active autonomous (intact) elements to allow the transposition of non-autonomous (deletion derivatives) elements, as long as they retain the *cis*-acting target sequences. In addition, TNPA can act as a positive and negative regulator of its own activity [3].

A 17 kb *Tam1 *CACTA-element of *Antirrhinum majus *also encodes two transcripts with somewhat parallel organization as the *Spm/En *element, an abundant 2.5 kb and a low abundance 5-kb mRNA [13]. The 2.5 kb transcript of *Tam1 *(known as *Tnp1*) is also pasted together from distant exons as is the *tnpA *mRNA of *Spm/En*; however, they have no sequence similarity. On the other hand, the larger transcripts of both elements containing sequences of the open reading frames (*Tnp2 *and *TnpD*), share significant (45%) amino acid homology [14] in the orf region that is found in the large Intron-1 of the maize *Spm/En *element. Similarly, previously reported non-autonomous CACTA-*Tgm*-elements of soybean feature a portion of an open reading frame 39% similar to the *tnpD *gene of *Spm/En *[15]. The conservation of these sequences suggests a common function of these gene products. It has been proposed but not proven that TNPD may interact with the conserved 13-bp TIRs and cleave the transposon termini [6,14].

Other active or full length CACTA elements have been isolated and characterized to some extent from several other species including, *Tpn1 *from *Ipomoea nil *[16],*Tdc1 *from *Daucus carota *[17], *PsI *from *Petunia hybrida *[18], *Candystripe1 *(*Cs1*) from *Sorghum bicolor *[19], *Atenspm1 *(or *CAC1*) from *Arabidopsis thaliana *[20], *Tpo1 *from *Lolium perenne *[21] and *TamRSA1 *of *A. majus *[22].

Only deletion derivative, non-autonomous CACTA family members (*Tgm1-Tgm7*) and *Tgm-Express1*, have been studied to date from soybean [23,15,25]. We recently identified the *T *locus in soybean that controls the tawny color of the trichome hairs on the plant leaves and stems as encoding a flavonoid 3' hydroxylase gene (*F3'H*) [26]. The stable gray t* allele, that was derived from a single mutable plant displaying both tawny and gray trichomes on the same plant, appeared to contain a large insertion that was not amplifiable by standard PCR conditions. Using PCR conditions designed to amplify large fragments, we here report finding a large 20.5 kb CACTA transposon (designated Tgmt*) inserted in Intron-1 of the *F3'H *gene, thus defining the novel t* mutant allele. Tgmt* had imperfect 13 bp CACTA TIRs and asymmetric subterminal repeats, and created a three base duplication upon insertion.

Comparison of the Tgmt* subterminal repeats to those of other reported CACTA elements of soybean and other plant species revealed wide divergence of the repeated motifs and the existence of two distinct CACTA transposon families in soybean. At the same time, all elements' subterminal repeated motifs had some regions of similarity. In addition, Tgmt* encodes a 14.6 kb complex Gene-1 with a transposase and a presumed DNA-binding function. A large precursor transcript encoded a transposase with tnp2 and TNP1 domains. Smaller alternatively spliced transcripts (2 – 2.6 kb) had little similarity to other CACTA transposon mosaic transcripts such as tnpA of maize and they likely encode a distinct type of binding protein that recognizes the soybean Tgmt* subtype of subterminal inverted repeats. Thus, in addition to broadening our understanding of the CACTA transposon's integral components, these findings point to Tgmt* as a potentially autonomous CACTA element isolated from soybean. Significantly, the data also clearly demonstrate diversification of the subterminal repeats of CACTA families within a species such as soybean (Tgmt* versus *Tgm1*), as well as between species as has been noted before for *Tgm1*, *En-1*, and *Tam1*, for example. This observation is substantiated not only by the divergence of the subterminal inverted repeats between the Tgmt* and *Tgm1-Tgm7 *type elements, but also by the divergence (76% similarity over a 1 kb segment) between the tnp2-containing orf of Tgmt* and the previously characterized partial tnp2-containing orf in the *Tgm5 *element. Thus, our data point to considerable within-species divergence of the CACTA element families. Whether these subtypes of element families originated before or after the origin of soybean as a distinct species is unknown. Bioinformatic analyses of these elements in many whole genome sequences within the legume family and other dicotyledonous plants in future years may shed light on how rapidly the DNA in the subterminal repeats and the tnpA-like exons that putatively recognize these subterminal repeats may coevolve and diversify both within and between species.

## Results

### Isolation of a large DNA insertion in the novel mutant allele with gray trichomes (t*)

In a previous study in which the *T *locus of soybean was identified as the flavonoid 3'-hydroxylase gene (*F3'H*), we reported the partial genomic sequences of three *F3'H *alleles of the soybean lines, Williams 43 (*T*, tawny), XB22A (T*, tawny) and its gray trichome isoline, 37609 (t*). We postulated that a large intron existed in the 5' region of the *F3'H *genes and that it prevented PCR amplification of the contiguous full length *F3'H *genomic sequence using standard methods. In order to obtain the full sequence of soybean *F3'H*, we isolated a BAC clone from cultivar PI437654 with the *T/T *genotype. The 64 kb of BAC sequence (to be detailed later) revealed a 4.3 kb intron in the *F3'*H gene. This information was utilized to synthesize primers intended to amplify the promoter and Intron-1 genomic regions of *F3'H *alleles in XB22A, 37609, 37643, 33745 and Williams 43 soybean lines (Table 1). The ~2 kb promoter region of *F3'H *in all those lines was identical although there were several differences from the PI437654 cultivar. The 4.1 kb Intron-1 sequence was also determined for the *F3'H *alleles of Williams 43 (*T*, tawny trichomes), XB22A (T*, tawny trichomes) and the stable gray-trichome isoline, 37609 (t*). The three *F3'H *full length genomic sequences: Williams 43 (*T*: 8,680 bp), XB22A (T*: 8,678 bp) and its isoline 37609 (t*: 29,609 bp) were entered in GenBank with accession numbers, EU190438, EU190439 and EU190440 respectively.

**Table 1:** Soybean cultivars and derived isolines: Phenotypes and genotypes

Cultivars	Genotype	Phenotype
XB22A	T*	Stable tawny trichomes
37609	t*	Stable gray trichomes
37643	t*	Stable gray trichomes
33745	*t*^*m*^	Mutable tawny/gray trichomes
		
Williams 43	*T*	Tawny trichomes

**Figure 1:**
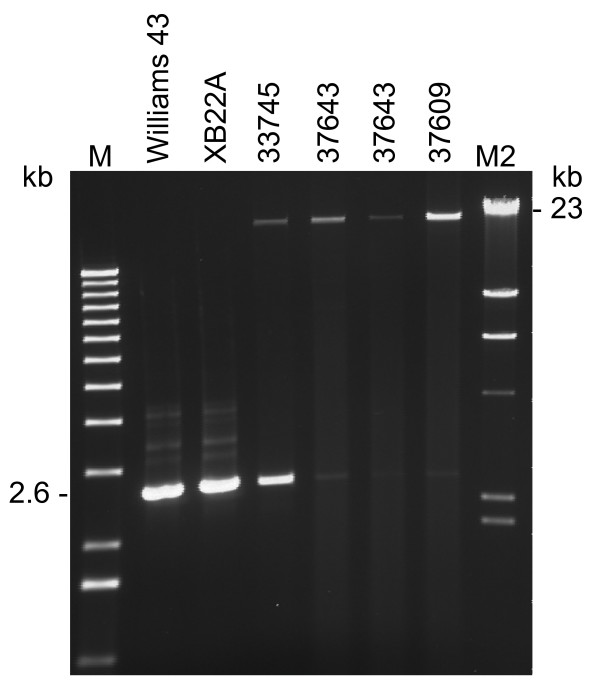
**Amplification of a large DNA insertion in Intron-1 of *F3'H *alleles**. DNA fragments amplified from genomic DNAs isolated from 5 soybean lines with different *T *locus alleles were visualized in this EtBr stained gel: Williams 43 (*T*), XB22A (T*), 33745 (*t*^*m*^), 37643 (t*) and 37609 (t*). The ~23 kb fragment contains a large insertion in Intron-1 of the t* allele. The 2.6 kb fragment is the Intron-1 DNA portion between the primers chosen for the PCR reactions (Z37F and IN663; see Additional file 5: Alignment of cDNA sequences of clones 43–53 to Tgmt* genomic sequence, and Methods). M is a 1 kb lambda DNA ladder. M2 is a *HindIII *lambda DNA marker.

**Figure 2:**
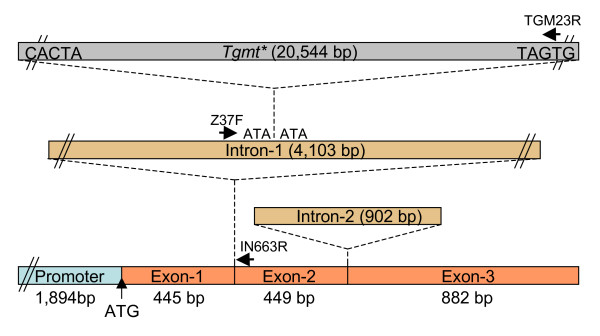
Schematic of the Tgmt* transposon insertion in the *F3'H *gene of the t* allele. A 20.5 kb transposon (Tgmt*) inserted near the center of Intron-1 is shown together with the location of primers (Z37F, IN663R) used to amplify it. The approximate location of a third primer (TGM23R) used with Z37F to amplify a 17 kb portion of Tgmt* is also indicated. The three primers are represented by solid black arrows. The parallel lines at the end of Tgmt*, Intron-1 and promoter's 5'-end indicate size reduction to fit the size scale of the drawing. The CACTA ends and the ATA target size duplication are also shown.

The dramatically differing sizes between the tawny (*T *and T*) and gray (t*) trichome *F3'H *alleles above was found to be due to a 20.5 kb insertion in Intron-1 in the 37609 mutant isoline (Figure 1). PCR amplification of the 20.5 kb insertion required the use of higher melting temperature oligonucleotide primers (Z37F and IN663R) and optimized conditions for *LA Taq *polymerase (TaKaRa Bio Inc.) as described in the Methods section. As shown in Figure 1, a large fragment of ~23 kb was amplified from genomic DNA from both of the stable gray trichome lines 37609 and 37643 as well as from the mutable tawny/gray trichome line, 33745 (Table 1). The same primers also amplified a smaller 2.6 kb fragment from the mutable 33745 line and those lines with the dominant alleles, Williams 43 and XB22A. The 2.6 kb fragment is the Intron-1 portion comprised between the two primers used in the PCR reactions. The insertion maps ~2.4 kb to the left of the IN663R primer (Figure 2).

### Cloning and sequencing a 20.5 kb CACTA transposon, Tgmt*

The ~23 kb PCR fragment was purified from 1% seaplaque agarose gel with the aid of GELase agarose gel-digesting preparation (Epicentre Biotechnologies), cut with *HindIII *and cloned in a pGem vector. Fortunately, two of the resulting clones that hybridized to a labeled probe prepared with an aliquot of the GELase purified 23 kb fragment, with sizes 199 bp, and 3,620 bp turned out to be the insertion's borders, each containing portions of intron sequence, a CACTA inverted repeat and the adjacent three base target duplication (ATA).

A 37-mer reverse primer (TGM23R) designed to the insertion's right border found in the 3.6 kb clone was paired with the Z37F primer upstream of the insertion's left border to amplify a 17 kb DNA fragment (Figure 2). An attempt at cloning the17 kb fragment into the pJAZZ-OC vector using the BigEasy v2.0 linear cloning kit from Lucigen Corp. failed to clone it in one piece but many partial clones ranging in size from 1 to 7 kb were recovered. Five different clones were sequenced and assembled to reveal a 20,544 bp CACTA transposable element that was named Tgmt* (Figure 2). This sequence was entered in GenBank as part of the t* allele genomic sequence from the 37609 line (Acc. No. EU190440). Previous studies of soybean clones containing CACTA ends analyzed seven distinct sequences (*Tgm1-Tgm7*) ranging in size from 1.6 to 12 kb that were believed to be portions of deletion derivatives of a larger active element (Rhodes and Vodkin, 1985 and 1988). Only *Tgm4 *and *Tgm5 *sequences had a 1 kb segment with 39% similarity to the ORF1 of maize *Spm/En *transposable element *tnpD *gene. The larger Tgmt* element we have cloned in this report has the capacity to encode all known functions required of an active and autonomous CACTA element as will be described later.

### Molecular structure of Tgmt*: terminal and subterminal repeats

**Figure 3:**
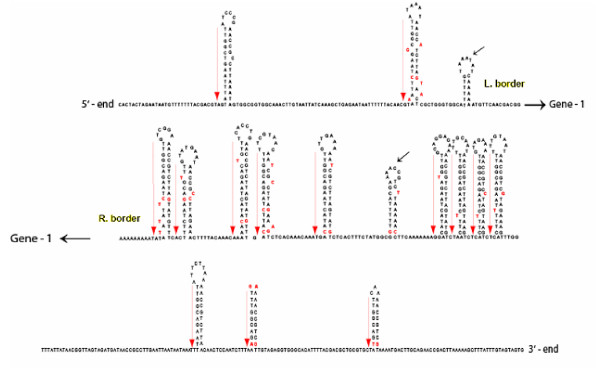
Regions of subterminal repeats in Tgmt* left and right borders. There are 4 subterminal sequence repeats in the left border in a stretch of 159 bp from the 5'-TIR. The right border contains 24 subterminal sequence repeats in a stretch of 732 bp from the 3'-TIR. These sequence repeats are capable of forming two palindromes in the left border and 12 in the right border. Red arrows mark the repeats shown in Figure 4. Red letters indicate base pair mismatches. An additional short stem loop in each border (marked with small solid black arrows) could be fold but the repeated sequences that form them are not related to the 28 repeats forming the other 14 stem loops. Thin black arrows point to the relative location of Tgmt* Gene-1.

**Figure 4:**
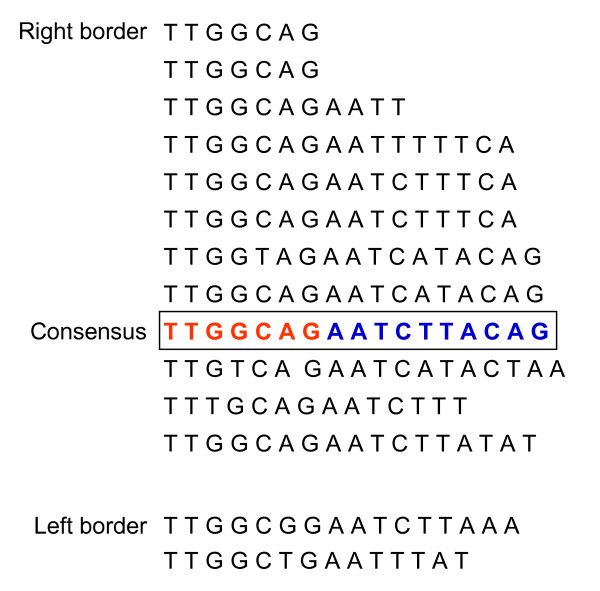
Alignment of Tgmt* subterminal direct repeats. The repeated sequences vary in length and they have been organized in this figure starting from the 3'end of the transposon right border. Each direct repeat was read from the 3'-end to the 5'-end. A consensus sequence motif was deduced and is shown boxed. This larger motif can be subdivided into two smaller sub-motifs. One is TTGGCAG that is present in all repeats (shown in red letters in the consensus sequence motif). The second, AATCTTACAG, is more divergent and absent in its entirety in two of the repeats (shown in blue letters in the consensus sequence motif).

Sequence analyses revealed features that are conserved among transposable elements of the *Spm/En *family. Tgmt* possesses nearly identical 13 bp CACTA inverted repeats with three reciprocal mismatches and features a three-base-target site duplication, ATA. It also contains asymmetric, reiterated direct and inverted sequence motif in the subterminal regions capable of forming 12 stem-loop structures in the right border and two in the left border (Figure 3). The largest stem-loop structures were formed by direct and inverted repeated sequence motif 17 bp long. This sequence motif could be divided in two sub-motifs, a 7 bp motif (TTGGCAG) present in all 14 stem-loop structures and a 10 bp motif (AATCTTACAG) that was missing or incomplete in some of the stem-loops. See an alignment of all direct stem repeated sequences (read from 3'-end to 5'-end) in Figure 4.

**Figure 5:**
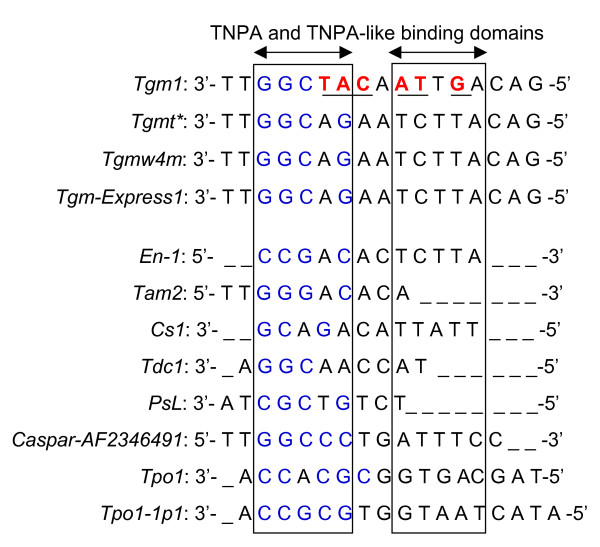
**Alignment of multiple CACTA transposon subterminal repeated motifs**. The consensus sequences of subterminal repeated motifs were aligned to determine the degree of divergence. The *Glycine max *(*Tgm*) transposon motifs fell into two subfamilies: *Tgm1 *had 6 base changes (shown in red) compared to the consensus motif of the Tgmt* family (Tgmt*, Tgmw4m and *Tgm-Express1*). The consensus sequences of the Tgmt* family members were identical. These *Tgm *motifs were compared to other published CACTA transposon's subterminal repeats. Two rectangles were use to demark the sequence portions of the motifs with some similarity, a GC rich (left) and AT rich (right). These sequence motifs are domains for TNPA-like proteins binding.

A similarly complex pattern of stem-loop structures was described for *Tgm1 *[15]. In this instance the length of the repeated sequence motif (17–20 bp) was more uniform (see Additional file 1: Alignment of *Tgm1 *subterminal direct repeats). The consensus sequence of these direct repeats from *Tgm1 *was compared to the one deduced from Tgmt*, *Tgm-Express1 *([25]; see Additional file 2: Alignment of *Tgm-Express1 *subterminal direct repeats) and *Tgmw4m *(GenBank Acc. No.: EU068464; see Additional file 3: Alignment of *Tgmw4m *subterminal direct repeats). Surprisingly, the *Tgm1 *sequence motif diverged from all three others in 6 nucleotide positions highlighted in red in Figure 5. The subterminal direct repeat motif of *En-1 *is smaller (12 bp) and except for a TCTTA domain its sequence is different from the motif of the soybean Tgmt* transposon family (*Tgm-Express1*and *Tgmw4m*) analyzed. The extended sequence divergence of *Tgm1 *is intriguing and suggests the existence of a second CACTA transposon family in soybean. This variability of subterminal repeats extends to other motifs reported. Figure 5 shows an alignment of subterminal repeats from 12 transposon sequences emphasizing both, their divergence and slight commonality. They all seem to have a GC rich and an AT rich portion. Whether or not these are recognition sites for the DNA-binding proteins remains to be determined.

### Molecular prediction of Tgmt* open reading frames

Softberry- FGeneSH gene prediction web-based algorithms using *Medicago truncatula *as training set  found one gene with 21 exons in the negative DNA strand relative to the *F3'H *gene (t*) where Tgmt* inserted. Thus, all sequence analysis description and depiction of Tgmt* Gene-1 is that of the reverse complement. The predicted Gene-1 first exon starts at base pair 6,123 and its polyA at 19,332 bp (Table 2). This gene could transcribe a 6,585 bp mRNA that would translate a 2,194 amino acid (aa) product. The derived aa sequence subjected to NCBI Non-redundant protein sequence database BLAST found similarity to many putative CACTA transposon proteins. The highest similarities (59% and 53%) were to *Vitis vinifera *hypothetical proteins CAN82870 and CAN66891 over a length of 1060 and 1400 aa respectively. The Tgmt* predicted protein had two putative conserved domains, a transposase family tnp2 domain [+](pfam02992) (E value 1e-80) located between 265 – 493 aa and a TNP1/EN/SPM transposase domain [+](pfam03017) (E value: 0.006) located at 1,493 – 1,534 aa. A search for similar domain architectures with NCBI CDART (Conserved Domain Architecture Retrival Tool) found two sequences from *Oryza sativa *(Os03g0714800) [27] to have the most closely related domain architectures. In contrast, the two *V. vinifera *hypothetical proteins with the highest similarity to Tgmt* Gene-1 product had only one transposase domain, the tnp2 domain.

**Table 2:** Softberry predicted gene exons and deduced from RT-PCR cDNA clones

Softberry-FGENESH Prediction					RT-PCR cDNA cloned sequences				
	
#		Start	End	Length	#		Start	End	Length
	TSS	4709				TSS	4708		
1	CDS f	6123	9287	**3165**	1	CDS f	6143	9319	**3177**
2	CDS i	9446	9753	**306**	2	CDS i	9446	9753	**308**
3	CDS i	9854	10066	**213**	3	CDS i	9854	10066	**213**
4	CDS i	10156	10419	**264**	4	CDS i	10156	10419	**264**
5	CDS i	10508	11200	**693**	5	CDS i	10508	11200	**693**
6	CDS i	11302	11555	**252**	6	CDS i	11302	11555	**254**
7	CDS i	11659	11826	**165**	7	CDS i	11659	11826	**168**
8	CDS i	11902	12006	**102**	8	CDS i	11902	12006	**105**
9	CDS i	12110	12182	**72**	9	CDS i	12110	12182	**73**
10	CDS i	12264	12359	**96**	10	CDS i	12264	12359	**96**
11	CDS i	12928	13028	99	11	CDS i	12446	13028	583
					12	CDS i	13113	13153	41
					13	CDS i	13254	13331	78
12	CDS i	13509	13671	**162**	14	CDS i	13505	13671	**167**
13	CDS i	13844	13948	**105**	15	CDS i	13844	13948	**105**
					16	CDS i	14160	14216	57
14	CDS i	14381	14461	**81**	17	CDS i	14381	14461	**81**
					18	CDS i	14711	14745	35
15	CDS i	15067	15156	90	19	CDS i	15067	15505	439
					20	CDS i	15614	15659	46
16	CDS i	16207	16482	**276**	21	CDS i	16207	16482	**276**
17	CDS i	16542	16573	30					
18	CDS i	17132	17314	**180**	22	CDS i	17132	17314	**183**
19	CDS i	17573	17681	108					
20	CDS i	18574	18621	**48**	23	CDS i	18574	18621	**48**
21	CDS l	19016	19072	**57**	24	CDS i	19016	19079	**64**
	PolA	19332				PolA	19335		

The soybean deletion derivative *Tgm5 *transposon sequence (Acc. No. X13528) has 79% similarity to the Tgmt* sequence stretch from 6,572 to 7,571 bp and contains the entire tnp2 transposase domain. It was previously estimated that *Tgm5 *was 39% similar to the ORF1 of the *Zea mays En-1 *transposable element [24].

When we applied Softberry- FGeneSH gene prediction web-based algorithms using *monocot plants *as training set  to the autonomous transposable element *En-1 *sequence [4], it predicted one gene with 12 exons, an mRNA of 5,190 bp and a protein of 1,729 aa. A Non-redundant protein sequence database BLAST search with the predicted 1,729 aa sequence found similarities to other maize transposable elements proteins and identified two transposase domains. One located at 264–491 aa, [+]pfam02992, transposase_21 transposase family tnp2 (E value: 7e-98) and the second at 1,391–1,487 aa, [+]pfam03004, transposase_24 plant transposase ptta/En/Spm family (E value: 8e-05). Thus, it appears that the predicted genes from Tgmt* and *En-1 *are similar at the 5'end with the tnp2 transposase domain at identical location (264(265)-491(493) aa). In contrast, the aa sequences of the predicted genes beyond the 780 aa, diverged significantly with two distinct, non aligned, transposase domains. Tgmt* and *En-1 *aa sequence alignment using the "Multiple sequence alignment with hierarchical clustering" (*MultAlin*) program [28], showed the similarities at the 5'end of the proteins with the tnp2 domains aligned and 258 identical aa's over the first 780 aa stretch (33% similar). The remaining 1,422 aa of the Tgmt* predicted protein had only 141 aa identities (10% similarity) to the *En-1 *predicted product and the TNP1/EN/SPM transposase domain of Tgmt* is different and did not align with the ptta/En/Spm domain of *En-1*. This second stretch of the aligned aa sequences included large gaps that would account for the smaller size of *En-1 *predicted gene and its product, 465 aa shorter. (see Additional file 4: Tgmt* and *En-1 *amino acid sequence alignment).

### Expression of Tgmt* Gene-1 in mutable and stable trichome color soybean isolines

Based on the molecular analysis of Tgmt* DNA sequence with web-based gene prediction algorithms and the extent of the similarity of the predicted gene to the well characterized gene of the *En-1 *autonomous CACTA transposable element, it was presumed that Tgmt* Gene-1 should be expressed in soybean lines where the putative autonomous Tgmt* element could be active. An initial attempt at determining Gene-1 expression was a search for RNAs hybridizing to Gene-1 DNA probes on RNA blots with samples extracted from multiple tissues of four soybean lines varying at the *T *locus, Williams 43 (*T*), XB22A (T*), 37609 (t*) and 33745 (*t*^*m*^) (Table 1). No clear, significant hybridization was detected in any of the RNA blots with any of the tested DNA probes which together covered the region of Gene-1 encoding Exons 1–7, with the tnp2 and TNP1 transposase domains (data not shown).

**Figure 6:**
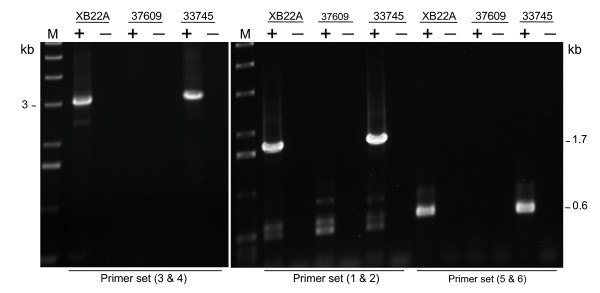
Tgmt* Gene-1 expression in isolines with differing *T*-locus alleles. DNA fragments amplified in RT-PCR reactions using three different sets of primers and RNAs from XB22A (T*), 37609 (t*) and 33745 (*t*^*m*^) isolines were visualized in an EtBr stained agarose gel. The primer numbers are indicated at the bottom of the figure for each set of RT-PCR reactions. See Methods for sequences of DNA primers. The primer's locations are indicated in Figure 8. The negative controls (-) were reactions in which the cDNA synthesis step was allowed in the absence of superscript.

**Figure 7:**
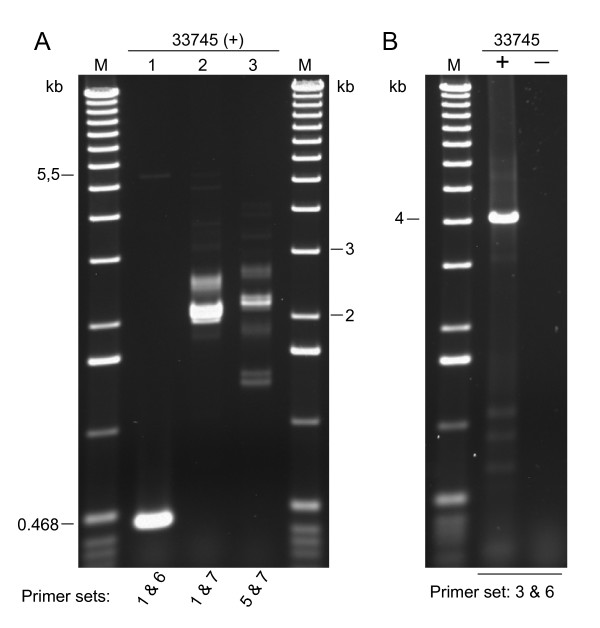
Tgmt* Gene-1 expression in mutable 33745 (*t*^*m*^) line. DNA fragments amplified in RT-PCR reactions using four different sets of primers and RNAs from the mutable 33745 (*t*^*m*^) line were visualized in EtBr stained agarose gels. The primer numbers are indicated at the bottom of the figure for each set of RT-PCR reactions. A) 1 and 6; 1 and 7; 5 and 7. B) 3 and 6. See Methods for sequences of DNA primers. The negative controls (-) were reactions in which the cDNA synthesis step was allowed in the absence of superscript.

**Figure 8:**
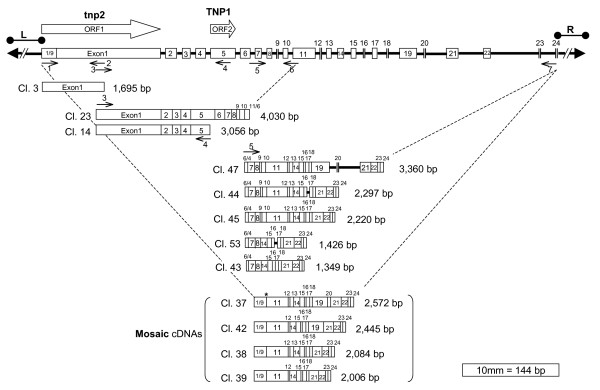
Schematic representation of Tgmt* Gene-1 and cloned cDNAs. The amplification products shown in Figures 5 and 6 were cloned and their sequences aligned to determine the exon-intron boundaries. Gene-1 larger exons are shown boxed and numbered on a solid line that represent the introns and the 5'- and 3'-ends of Gene-1. The smallest exons (-9, -12, -16, -18, -20, -23, -24) are represented by two parallel lines. Left (L) and right (R) borders are reduced in size to fit the size scale. L is 6,122 bp and R, 1,465 bp. Solid arrow heads represent the CACTA terminal inverted repeats. The two unfilled arrows above the diagram of the transposon represent ORF1 with the transposase tnp2 domain (tnp2) and ORF2 with the TNP1 transposase domain. Below the Tgmt* diagram are the cDNAs amplified with the different sets of primers shown in Figures 6 and 7. The numbered primers are sketched with small arrows underneath Tgmt* or near the cDNAs to simplify the figure. The splice site of the mosaic cDNAs amplified with primers 1 and 7 is indicated with an asterisk (*). The 1/9 portion of Exon-1 represents the 346 bp of Exon-1 that are spliced to Intron-10 right border. Cl = clone number.

These results suggested that if the transposase gene was expressed it did so at very low levels, and thus we opted for the more sensitive reverse transcriptase polymerase chain reaction technique (RT-PCR) to assay Gene-1 expression. Figure 6 shows the amplification products obtained using three primer pairs. Each pair amplified a different portion of the Gene-1 region with the tnp2 and TNP1 domains. The RT-PCR reactions shown were carried out with RNAs from three isolines that were derived from a single rogue soybean plant that had shown variegation in trichome color in a field breeding program: XB22A (T*), 37609 (t*) and 33745 (*t*^*m*^) (Table 1). The negative controls (-) were reactions where the cDNA synthesis step was allowed in the absence of superscript (See Methods). Interestingly, the 37609 line with the recessive allele (t*) from which Tgmt* was isolated did not appear to express Gene-1. In contrast, XB22A (T*) and 33745 (*t*^*m*^) isolines seem to have retained a putatively active Tgmt* expressing the transposase region of Gene-1. This differential pattern of RT-PCR amplification amongst the isolines was repeated with several other primer sets tested covering other regions of Gene-1 (data not shown). Figure 7 shows results of additional examples of PCR reactions performed with cDNAs synthesized from RNA of the mutable isoline 33745 (*t*^*m*^) and that generated larger or more complex DNA fragment patterns. Lane 2 of Figure 7A shows the range of amplification products (~2- 5.5 kb) obtained with two primers (1 and 7) each at either end of the predicted Gene-1. Many of the products amplified from cDNAs of this mutable line, 33745 (*t*^*m*^), were cloned and sequenced and the analysis and assembly of the cDNA sequences provided a clear picture of Tgmt* expression which is shown in Figure 8. The location of all primer pairs used in the PCR reactions which results are shown in Figures 6 and 7 were marked in Figure 8 with small arrows numbered from 1–7. In certain instances the arrows were placed near the clones (Cl. 23, 14 and 47) to simplify the diagram.

The sequences of 17 cDNA fragments were used to map all the exons and introns on the genomic Tgmt* sequence. The exon-intron boundaries obey the canonical GT-AG rule [29] in the majority of the cDNA clones. See Additional files 5 and 6 where exon sequences of cDNA clones have been aligned with the Tgmt* genomic sequence. The sizes and locations of the cDNA exons are listed in Table 2 and highlighted in bold are 17 of them that are almost identical in size and location to exons predicted by the Softberry- FGeneSH program. Two of the predicted Exons (No. 11 and 15) were portions of larger exons in the cloned cDNAs (No. 11 and 19). However, predicted Exons No. 17 and 19 towards the 3' end of the gene were not part of the cloned cDNAs and since we recovered 11 different cDNA clones from this region of the gene it can be assume with certainty that predicted Exons-17 and -19 were errors of the FGeneSH program. A full length cDNA of 7,554 bp spanning the predicted Gene-1 could be assembled with all the exon sequences of the cDNA clones (see Additional file 7: Tgmt* Gene-1 precursor transcripts sequence). Most likely, transcripts of this size were synthesized in vivo given the RT-PCR results obtained and the locations of the primers used on the gene sequence (Figures 7 and 8).

Of most relevance were the sequences of the amplified cDNAs with primers at the ends of the gene (1 and 7) because they revealed splicing of the first 346 bp (1/9 portion) of Exon-1 with Exon-11 to generate transcripts 2 – 2.5 kb in size (Figure 8, Mosaic cDNAs, Cl. 37 – 39; see Additional file 8: Tgmt* Mosaic-transcript sequence). The splicing of the 346 bp of Exon-1, ending in ATAAT, did not occur at the intron-exon junction of Exon-11 but rather 23 bp into Intron-10 right border, adding the 23 bp of intron sequence in the synthesis of mosaic transcripts. The Intron-10 sequence up-stream of the splice site also ends on ATAAT, the same motif found up-stream of Exon-1 splice site (see Additional file 9: Tgmt* genomic sequence (20,544 bp)). Whether or not this sequence motif is involved in the recognition and splicing mechanism to create the mosaic gene is not known.

The splicing of all the many exons to form the mosaic transcripts as well as the full length precursor transcripts must be cumbersome judging by the multiplicity of related cDNAs amplified with a given set of primers (Figure 7 A, lanes 2 and 3; Figure 8, mosaic gene transcripts (Cl. 37–39) and transcripts amplified with primers 5 and 7, Cl. 47-43). Some of the cDNAs retained entire introns such as Cl. 47 or intron portions in Cl. 44 and 53 (Figure 8). Exon-19 was spliced out in six of the cDNA clones sequenced (Cl.44-43 and 38 and 39). This erratic splicing of exons was observed earlier in transcripts from another complex soybean CACTA transposon,*Tgm-Express1 *containing multiple host-gene fragments [25,30].

Nevertheless, a putative full length mosaic cDNA of 2,572 bp was cloned and sequenced (Figure 8 and see Additional file 8: Tgmt* Mosaic-transcript sequence). The splice site between Exon-1 (first 1/9 portion of the exon) and Exon-11 is indicated with an asterisk in Figure 8. This mosaic transcript may encode the DNA-binding protein that is required for excision of these CACTA transposons in soybean in the same fashion TNPA does it for the *En-1 *element of maize. NCBI/BLAST/blastx (version 2.2.18) found similarities to some predicted proteins from *V. vinifera*, *Arabidopsis *and *O. sativa *in the non-redundant protein database but not to maize. This result supports previous observations that proteins that share little similarity, such as TNPA and TNP1, may have a similar function, that of binding to the subterminal repeats, regions that vary considerably amongst the different CACTA transposons [31].

In summary, Tgmt* expresses the predicted Gene-1 in XB22A (T*) and the mutable 33745 (*t*^*m*^) lines, but not in the stable, gray trichome 37609 (t*) isoline from which it was cloned. A multiplicity of mRNAs, were transcribed from Gene-1 and the putative largest transcript assembled was 7,554 bp in length. In addition, mosaic transcripts of different sizes were also synthesized and the largest was 2,572 bp. The precursor mRNA codes for a transposase with a tnp2 and TNP1 domains in Exons-1 and 5 respectively. The 2,572 mosaic transcript may encode a DNA-binding protein with a function similar to that of TNPA of the maize *En-1 *and TNP1 of *A. maju*s *Tam3*, but with very limited amino acid sequence similarity.

### Redundancy of Tgmt*-like sequences in the soybean genome

In order to determine the abundance of Tgmt* sequences in the soybean genome, an initial NCBI/BLAST/blastn search of the nucleotide collection (nr/nt) database optimized for highly similar sequences was run using either the entire 20,544 bp Tgmt* sequence, the 5,078 bp comprising Exon-1 through Exon-5 with the transposases ORF1 and ORF2 or the 7,778 bp starting at Exon-11 through Exon-24. The transposase portion of the element had 84% identity with 98% coverage to a *Glycine max *clone gmw1-45m6, (AC166742.25), 75% identity over a 58% coverage to two *Glycine tomentella *clones gtt1-62b6 (AC188785.18) and gtt1-188p11 (AC183809.15), 75%identity and 26% coverage to a *Glycine max *clone BAC GM_WBb080D (EF533700.1), and finally, 76% identity over 17% coverage, to the soybean *Tgm5 *transposable element (X13528.1). However, no highly similar sequences were found when the BLAST search was performed with the second half of Gene-1 sequence (7,778 bp). Two additional sequences, one from *Glycine max *cultivar T322 dihydroflavonol-4-reductase 2 (*DFR2*) gene, Intron II and transposon *Tgmw4m *(EU068464.1) and a second one from *Glycine max *cultivar T321 dihydroflavonol-4-reductase 2 (*DFR2*) gene, DFR2-w4-dp allele, disrupted promoter region and transposon *Tgmw4m *(EU068463.1) had 99% identity to the right and left border of Tgmt* over 1,325 and 975 bases, respectively, indicating that these may be deletion derivatives from an element similar to Tgmt*.

The search was extended to the 7× draft sequence assembly Glyma0 from the Joint Genome Inititive (JGI version 12/6/07, see methods). The blastn search with the Tgmt* entire sequence (20.5 kb), found high level of similarity to 52 scaffolds (E value = 0.0). To determine how many of the 52 scaffolds had the transposase and the exons of the mosaic transcript, two blastn searches with the 5,078 bp (Exon-1 through Exon-5) and the 7,778 bp (Exon-11 through Exon-24) were performed. The transposase exons found high similarity (E value = 0.0) to 45 scaffolds while the exons of the mosaic transcript found high similarity (E value = 0.0) to 15 scaffolds. Scaffolds with high similarities to both halves of Tgmt* Gene-1 were, Scaffold_4, _81, _24, and _5. Thus, it appears that the soybean genome of cultivar Williams has at least 4 regions with extensive sequence similarity to Tgmt*.

We examined also the regions of similarity in the Glyma0 soybean genome (JGI 7× draft sequence assembly) to the *Tgm5 *transposase sequence (Ac. No. X13528.1) and the equivalent sequence region of Tgmt* (932 bp) using the Phytozome *Glycine max *(v3.0) BLAST search . We found that the two transposons had similarities to two different sets of scaffolds. *Tgm5 *transposase had the highest similarities to scaffolds_38, _20, _10, _229, _7, _12, _97 and _31 while Tgmt* transposase had the highest similarities to scaffolds_24, _4, _81, _6, _5 and _15. This distinction is additional supporting evidence that *Tgm5 *and Tgmt* are elements representing two different CACTA families in soybean.

A closer examination of the regions of similarities in each one of the scaffolds revealed a large number of partial transposase sequence copies in each one of those scaffolds when the 932 bp segment carrying the tnp2 domain was used as query. For *Tgm5 *scaffolds_38, _20, _10, _229, _7, _12, _97 and _31 there were 50, 37, 47, 6, 31, 23, 6 and 31 copies respectively. For Tgmt* scaffolds_24, _4, _81, _6, _5 and _15 there were 27, 55, 13, 53, 55 and 3 copies respectively. However most of these were relatively short regions not containing the element ends. Thus it appears that some regions of the 932 bp orf that carries the conserved tnp2 domain are widely dispersed in the soybean genome. These observations of moderately high copy number is supported by the intense hybridization signals of varying sizes obtained when this region of the element is used as a probe on genomic DNA blots. Furthermore, this region also hybridized to many clones when used to screen genomic libraries (unpublished observations).

### Isolation and characterization of a *T *locus BAC clone

**Figure 9:**
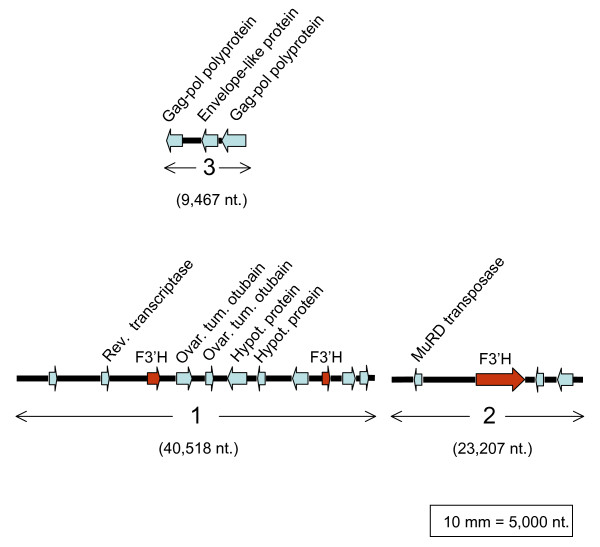
**Schematic representation of *F3'H *gene sequences in the PI437654 line BAC clone**. Three non overlapping sequence contigs-1 (40,518 nt),-2 (23,207 nt) and-3 (9,467 nt) of 71B1 BAC clone were arranged based on the organization of corresponding sequences in scaffold_83 of the 7× Glyma0 sequence assembly (JGI) of cultivar Williams. Contig-1 and -2 sequences are adjacent in Glyma0 assembly in the order shown. Contig-3 sequence is an insertion in the center of contig-1 in the Glyma0 assembly and here it is displayed a top and center of contig-1 to indicate the likely approximate location in 71B1 BAC clone. The sizes and orientations of genes are represented by gray arrows with their respective annotations. *F3'H *sequences are shown in red. The full length (1,590 bp) *F3'H *gene maps to Contig-2, and the smaller (504 and 354 bp) fragments with homology to *F3'H *map to Contig-1. Contig-3 encodes functions of a retrotransposon. The introns of genes are not displayed.

As previously discussed, to fully determine the molecular defect of the *F3'H *allele (t*) with the gray trichome phenotype, it required the cloning of a full length *F3'H *gene including its promoter. With such an aim, two *Glycine max *BAC libraries, one from Williams 82 (GMWBa libarary) and the other from the more distantly related plant introduction line PI437654 (Clemson University Genomic Institute) were screened. No clones were obtained from the Williams 82 GMWBa library but a BAC clone (71B1) containing a full length *F3'H *gene copy was isolated from the PI437654 library. Partial sequence of the *F3'H *gene in 71B1 clone was determined initially from PCR amplified fragments and later confirmed and extended with sequences resulting from a shotgun library of the entire BAC clone. These sequences were assembled into three contigs, most likely arranged as shown in Figure 9 (Acc. No.: EU721743). Softberry FGeneSH gene prediction and annotation web-based program identified and placed the full *F3'H *copy (1,590 bp) of the gene in contig-2. In addition, two smaller fragments with *F3'H *similarity (504 and 354 bp) mapped to contig-1. All three *F3'H *sequences are marked with red arrows in Figure 9. All other genes found in 71B1 clone were also annotated and their size and relative location in the three contigs are depicted in Figure 9 and listed in the annotation Table 3. As noted, other full copy genes in this BAC clone were three located in tandem in contig-3 encoding functions of a retrotransposon (gag-pol polyprotein and envelop-like protein). The assembly of the sequences resulting from the shotgun library did not overlap the three contigs shown in Figure 9 and their order could not be determine. With the recent release of the 7× draft sequence assembly (JGI version 12/6/07) (see Methods) from the soybean genome of cultivar Williams 82, a BLASTn search was run with the sequences of the three contigs from 71B1 BAC clone. It was determined that contigs-1 and -2 are adjacent in scaffold_83 and contig-3 sequence is an insertion in the middle of contig-1. For that reason contig-3 was drawn above contig-1 in Figure 9. Because the sequences with high similarity to the three contigs mapped closely together in scaffold_83, we can presume conservation of this region in the two cultivars, Williams 82 and PI437654 (see Additional file 10: Alignment of sequences of 3 contigs from 71B1 BAC clone in the 7× draft sequence assembly (JGI)).

**Table 3:** Gene annotation of BAC clone 71B1 from the *Glycine max *PI437654 library

**Contig**	**Gene No**.	**Strand**	**Exons**	**Nt**.	**AA**	**Annotation**	**Acc. No**.	**E-value**	**Organism**
1	1	(+)	1	351	116	Unknown Protein			
	2	(+)	1	459	152	Reverse transcriptase	ABB00038	2e^-79^	*Glycine max*
	3	(+)	4	504	167	F3'H	BAB83261	4e^-47^	*Glycine max*
	4	(+)	4	290	156	Ovarian tumor otubain	ABN05752	1e^-28^	*M. truncatula*
	5	(+)	1	387	128	Ovarian tumor otubain	ABN05752	3e^-19^	*M. truncatula*
	6	(-)	4	1407	468	Hypothetical protein	CAN67561	8e^-14^	*Vitis vinifera*
	7	(-)	2	693	230	Hypothetical protein	CAN70327	4e^-34^	*Vitis vinifera*
	8	(-)	5	432	143	Unknown protein			
	9	(+)	2	354	117	F3'H	AA047853	5e^-43^	*Glycine max*
	10	(+)	2	723	240	Unknown protein			
	11	(-)	3	801	266	Unknown protein			
									
2	1	(-)	2	381	126	MuRD transposase	AAC26234	2e^-06^	*Arabidopsis*
	2	(+)	4	1590	529	F3'H	BAB83261	0	*Glycine max*
	3	(-)	1	309	102	Unknown protein			
	4	(-)	3	435	144	Unknown protein			
									
3	1	(-)	1	2181	726	Gag-pol polyprotein	AA073523	0	*Glycine max*
	2	(-)	1	1851	616	Envelope-like protein	AA073528	0	*Glycine max*
	3	(-)	1	3018	1005	Gag-pol polyprotein	AA073521	0	*Glycine max*

We reported earlier that the *F3'H *gene appeared to be single copy in soybean [26]. The results of a BLAST search of the 7× draft sequence assembly (JGI version 12/16/07, see Methods) from the soybean genome of cultivar Williams with the Williams *F3'H *gene sequence (Ac. No.: EU190438) and the sequence of BAC clone 71B1, all pointing to a single Scaffold_83 with only one full *F3'H *gene copy, are further evidence that *F3'H *is a single copy gene in soybean. These results also explain the gray trichome phenotype of the soybean line in which the large Tgmt* element inserted in Intron-1 of the single copy of the *F3'H *gene in soybean.

## Discussion

Although the molecular structures of 7 deletion derivative CACTA transposable elements (*Tgm1-7*; [23,24,15]) as well as that of a *Tgm-Express1 *CACTA transposon of 5.7 kb that carries 5 gene fragments [25] have been described for the soybean, the existence of an autonomous element has remained elusive. A recent study that helped identify the *T *locus as a flavonoid 3'-hydroxylase (*F3'H*) gene was based on the sequence and expression of two different recessive alleles [26] that specify gray instead of tawny color trichomes. The *t *allele of Richland was characterized molecularly and was found to have a base deletion that creates a frame shift and terminates the F3'H open reading frame prematurely. The second gray trichome allele studied (t*) was derived from a different genetic stock, line 37609, that had undetectable F3'H mRNA levels but no significant differences in the genomic sequence of the two F3'H fragments we had isolated and analyzed. The 37609 stable, gray-trichome, line, was originally derived from a single rogue plant having chimeric sectors of both tawny and gray trichomes on the same plant that appeared spontaneously in the field of a breading program. A stable tawny line, XB22A (T*) and the gray/tawny mutable 33745 (*t*^*m*^) line were also derived from that initial rogue plant. The continued mutability of this isoline and the spontaneous appearance of the rogue progenitor plant suggested the existence of an autonomous transposable element in this soybean genetic stock that manifested in the mixed trichome phenotype.

As we had predicted in the above mentioned study, the portion of *F3'H *genomic sequence that eluded us at that time was a 4.1 kb Intron-1 in which a CACTA tranposon, 20.5 kb in size, had inserted in the gray trichome allele (t*) (Figure 2). This large insertion could have prevented proper splicing of Intron-1 and assembly of a functional F3'H mRNA. The mutable line 33745 has the gray (t*) and tawny (T*) alleles in its genetic make-up (Figure 1). The 20.5 kb element was named Tgmt* and its isolation through long-distance PCR amplification permitted its sequencing, molecular characterization and the study of its expression in the mutable and the stable gray or tawny trichome isolines.

Tgmt* has imperfect 13 base CACTA inverted repeats, a target site duplication (ATA) and the asymmetric highly structured subterminal regions characteristic of the CACTA transposon family. The 13 bp CACTA inverted repeats and the reiterated subterminal sequence motif, are *cis*-determinants for transposition. Unlike the 13 bp CACTA inverted repeats that are conserved, the subterminal repeats vary in size and sequence among the different CACTA transposons. The subterminal repeated motifs of the soybean CACTA elements analyzed (Tgm1, Tgmt*, *Tgm-Express1*and *Tgmw4m*) were more complex than the *En-1 *motif. It has been proposed that a DNA-binding protein encoded by the transposon attaches to these subterminal motifs to help or suppress the element's excision [31]. Two of the well characterized DNA binding proteins TNPA of *En-1 *and TNP1 of *Tam1 *shared little similarity and it was suggested that non-homologous DNA binding proteins may recognize diverse subterminal DNA-binding motifs [32]. The Tgmt* subterminal repeated sequence motif is not only different from that of *En-1 *transposon but it diverged from the repeated motif in another soybean CACTA element, *Tgm1 *[24,32] (Figure 5). Our results, thus, reinforce the notion of variability in subterminal regions of a CACTA element that is likely paralleled by DNA-binding protein diversity. In addition, the difference observed between the *Tgm1 *subterminal repeat motif and that of the Tgmt* family (*Tgm-Express1*and *Tgmw4m*), suggests that *Tgm1 *is a deletion derivative from another CACTA element distancing from Tgmt*. Could the TNPA-like DNA binding protein encoded in Tgmt* help in the excision/suppression of *Tgm1*? Although the repeated sequence motifs are different among all CACTA transposons characterized, it is possible that there is some communality among them that is recognized by the dissimilar TNPA-like proteins. The alignment of all subterminal repeated motifs shown in Figure 5 showed some broad similarities with a GC rich and AT rich domains. The GC rich domain may also be a site susceptible to methylation which could hinder TNPA-like binding and consequent inhibition of transposon excision [6].

The large and complex Gene-1 found in the negative strand through gene prediction web-based algorithms was confirmed by RT-PCR amplified cDNAs. It is a dual function gene with 24 exons stretching a length of 14,627 bp from the start site (4,708 bp) to the PolyA (19,335 bp) out of the 20,544 bp Tgmt* element. A precursor transcript of ~7.5 kb codes for a putative transposase with a tnp2 and a TNP1domains. A smaller mRNA of ~2.5 kb resulted from alternatively splicing the 5'end (346 bp) of Exon-1 to the 3'end of Intron-10, 23 bp upstream of Exon-11 (Figure 8; see Additional file 9: Tgmt* genomic sequence (20,544 bp)). The product of this mosaic transcript had no conserved domains and in a NCBI basic blastx the highest similarity was to a hypothetical *V.vinifera *protein (CA048701) with 79% similarities over a stretch of 428 aa. Likewise, the transposase mRNA in a similar blastx had 64% similarity to a *V. vinifera *hypothetical protein (CAN82870). In addition, the domain architecture of the transposase is most similar to that of an *O. sativa *protein (Os03g0714800) with a (pfam02992) tnp2 motif and a (pfam03017) TNP1/EN/SPM motif. These results suggest that the soybean Tgmt* Gene-1 is more closely related to genes in *V. vinifera*, *O.sativa *and *Arabidopsis *than to those of *Z. mays*, *En-1 *and *A. majus*, *Tam1*. *En-1 *transposase has also two domains, the (pfam02992) tnp2 that seems to be conserved in all CACTA element transposases, but the second domain is of the (pfam03004) Ptta/En/Spm family.

Another distinction of Tgmt* is that the 82% high GC content of Exon-1 (between positions 300 and 550) in *En-1 *element does not occur. Because CpG residues are sensitive to methylation, that region of *En-1 *with high GC content was proposed as potential site for gene regulation. The GC content of Tgmt* Gene-1 is uniformly lower with a 40% average throughout. Thus, transposon inactivation by methylation may not require such large concentration of CpG residues.

Although the majority of intron-exon splicing occurs at the canonical GT-AG boundaries, it is of interest to point out that the splice signals used to create the ~2.5 kb mosaic transcripts do not appear to be either the GT-AG for intron splicing or the CTPuAPy branch site signal typically located 20–50 bp upstream of the acceptor site where Pu = A or G and Py = C or T [33]. We do not know if the ATAAT motifs adjacent to both the donor and acceptor splice sites are used as recognition signals in Tgmt*. Alternatively, the splicing mechanism in this transposon may resemble the sex lethal gene model of the fruit fly where splicing signals may be masked by a regulatory protein [34]. This type of mechanism could have evolved to help reduce the number of transposition events. A regulatory protein that binds to Exon-1 blocking transcription of the transposase gene Orf1 and Orf2 would nonetheless allow splicing of the mosaic transcript. This blockage could result in reduction of transposon excision while allowing the suppressor function of the mosaic protein that will bind to the subterminal repeats of the element when possible.

The copy number of CACTA elements in *Z. mays *has been estimated to 50–100 [8] and 4 (20)*A. thaliana *[20]. The soybean *Tgm *family is also repetitive with earlier estimates from DNA blots using the element ends as probes, determining that the family of deletion derivatives numbered less than 50 copies per genome [15]. That figure may have been an underestimate as it likely included only the *Tgm1 *family of elements, excluding all those elements of the Tgmt* family. Our BLAST search also showed that the transposase first orf is very repetitive throughout the soybean genome. However, based on RNA blot results probed with Exon-1 sequence as well as the under-representation in the soybean expressed sequence tag (EST) collections, the number of active elements seems to be very low. It is possible that there are multiple autonomous *Tgm *elements in soybean but that they are inactivated by methylation under optimal growth conditions and re-activated in stressful environments. Precedent for this has been shown with rice retrotransposons where the copy number increased from 2 to 30 in some strains as measured by DNA blots [35].

On support of the possible existence of multiple autonomous elements in the soybean genome is the result obtained from a BLAST search of the 7× draft sequence assembly Glyma0 (JGI) that produced 4 regions (Scaffold_4, _81, _24, and _5) with significant similarities to the Tgmt* Gene-1 sequence.

## Conclusion

We have determined the molecular bases for the soybean gray trichome phenotype of the mutant t* allele to be a 20.5 kb putative CACTA transposon (Tgmt*) inserted in Intron-1 of the single copy *F3'H *gene. Tgmt* has conserved 13 bp TIRs, the 3 bp target site duplication and asymmetric subterminal repeated motif. The latter distinguished two CACTA-transposon families in soybean and defined regions of some similarity among other CACTA transposons subterminal motifs. Tgmt* expressed a 14.6 kb Gene-1 that encodes the two functions required of an active, autonomous element: a transposase with a conserved Tnp2 domain and a mosaic transcript bearing little homology to other transposon mosaic gene products. Thus, Tgmt* has the potential to be an active and autonomous transposon expressed in two isolines of the genetic stock studied. Tgmt* transposase is more closely related to *O. sativa *and *A. Thaliana *transposases with a TNP1 (pfam03017) domain than to *En-1 *transposase with a Ptta (pfam03004) domain.

In addition, our results support previous assertions that CACTA transposases are conserved, most likely because they associate with the conserved CACTA TIRs during scission and insertion, while the DNA-binding proteins, products of mosaic transcripts that bind to subterminal less-conserved repeated motifs, share little similarity. The divergence of the subterminal repeats within soybean as exemplified by the two subtypes of *Tgm1 *versus Tgmt* indicate that within-species diversification into subtypes of subterminal repeats and the mosaic transcript products that bind them, will be more extensively found as more genomes are sequenced and analyzed.

## Methods

### Plant Material and Genotypes

The *Glycine max *cultivars and isolines used for this study were: Williams (*T*, tawny trichomes) XB22A (T*, tawny trichomes), 37609 (t* gray trichomes), 37643 (t*, gray trichomes) and 37345 (*t*^*m*^, with variegated hilum tawny and gray trichomes). Each is homozygous for the indicated alleles of the *T *locus. The origin, genetics, and discovery of the *T *allele as *F3'H *has been described previously [26]. Plants were grown in the greenhouse. Shoot tips (meristems surrounded by primordial leaves), seed coats and cotyledons dissected from seeds at varying stages of development were frozen in liquid nitrogen, freeze dried (Multi-dry lyophilazer; FTS systems), and stored at -20°C. The seed coats and cotyledons used in this study were those of seeds which fresh weight of the entire seed was 25–50 mg.

### RNA Extraction, Purification and cDNA Synthesis

Total RNA was isolated from shoot tips, seed coats and cotyledons using a phenol-chloroform and lithium chloride precipitation method [36,37]. RNA was stored at -70°C until used.

cDNA copies of the Tgmt* predicted gene(s) from three of the isolines (XB22A, 37609, 33745) and Williams 43 were amplified from a first-strand cDNA pool synthesized using 1 μg of cotyledon total RNA and the Superscript first strand synthesis system for reverse transcriptase (RT)-PCR (Invitrogen, San Diego). The total RNAs used for these RT-PCR reactions were treated with DNAaseI using Ambion's DNA-free kit and concentrated in Microcon YM-30 columns (Millipore, Bedford, MA). For each RNA sample, parallel reactions were allowed in the absence of superscript (- controls) to assess the extent of DNA contamination. The sequences of primer pairs used in RT-PCR reactions shown in Figure 6 were:

(1) Primer No. 3 5'-GGGACATCTGAGAATGAC-3' (TGME3-43F)

Primer No. 4 5'-AACAACATACAATCCCAT-3' (TGME3-36R)

(2) Primer No. 1: 5'-AACAGCACGCATAACTGAAGA-3' (TGM8-14677R)

Primer No. 2: 5'-AACTGGTGTCGTACTCCC-3' (43-43-FR)

(3) Primer No. 5: 5'-GGCTGAAATAATATCAGG-3' (TGME-36NZ-F)

Primer No. 6: 5'-CATCATATTTAGCATCTG-3' (43-43-R2)

Primer pairs of RT-PCR reactions shown in Figure 7A and 7B were:

(A1) Primer No. 1: 5'-AACAGCACGCATAACTGAAGA-3' (TGM8-14677R)

Primer No. 6: 5'-TTAGCATCTGTTATTTCTAATAGC-3' (TRP-1-F) ≅ (43-43-R2)

(A2) Primer No. 1: 5'-AACAGCACGCATAACTGAAGA-3' (TGM8-14677R)

Primer No.7: 5'-TGTGAAGTCATATAGAGTGGC-3' (TGM8-1741F)

(A3) Primer No. 5: 5'-GGCTGAAATAATATCAGG-3' (TGME-36NZ-F)

Primer No.7: 5'-TGTGAAGTCATATAGAGTGGC-3' (TGM8-1741F)

(B) Primer No. 3 5'-GGGACATCTGAGAATGAC-3' (TGME3-43F)

Primer No. 6: 5'-TTAGCATCTGTTATTTCTAATAGC-3' (TRP-1-F) ≅ (43-43-R2)

### Primer synthesis, PCR reaction conditions and DNA cloning

Oligonucleotide primers were synthesized on an Applied Biosystems (Foster City, CA) model 394A DNA synthesizer at the Keck Center, a unit of the University of Illinois Biotechnology Center. For small DNA fragment amplification, PCR reactions were performed by an initial denaturation step at 94°C for 2 min followed by 30 cycles of denaturing at 94°C for 30 sec, annealing at 56°C for 1 min, extension at 68°C for 9 min, to end with a 10 min extension at 72°C. High-fidelity and -efficiency *Ex Taq *(Takara Bio Inc. Otsu, Japan) polymerase was used at 0.75 units per 50 μl reaction. To amplify the larger DNA fragments, PCR reaction conditions were as follows: initial denaturation step at 94°C for 2 min followed by 30 cycles of 94°C for 30 sec and 68°C for 10 min, and ending with a 10 min extension at 72°C. *LA Taq *polymerase (Takara Bio Inc. Otsu, Japan) was used at 0.75 units per 50 μl reaction.

In most instances, amplified DNAs were separated from oligonucleotides with a QIAquick PCR Purification kit (QIAGEN), cloned into pGem-T-easy and sequenced in an ABI 3730 × l (Applied Biosystems, Inc. Foster City, CA) at the Keck Center. However, the larger, 23 kb PCR product (primers: Z37F, 5'-ATTTGAAACGCGTGGTGCCTGCATTTAAAGACAA

TTT-3' and IN663R, 5'-ATCACCTCCATCACCATAGCCTTAAACTCATCAGCCC-3') was extracted from 1% Seaplaque agarose gel in TA buffer with the aid of GELase Agarose Gel-Digesting Preparation (Epicenter Biot. Madison, WI) following the manufacturer's protocol. Two equal fractions (200 ng) of the 23 kb DNA fragment extracted in this fashion was utilized to prepare a radiolabel probe and in *HindIII *restriction digests. The resulting *HindIII *fragments were cleaned with QIAquick PCR Purification kit (QIAGEN) and cloned into CIP dephosphorylated pGem vector cut with *HindIII*. A DNA ligation kit Ver.2.1 (TaKaRa) aided the cloning step. The resulting 23 kb *HindIII *clones were verified in DNA blots hybridized to the radiolabel 23 kb probe.

The conditions used to optimize the cloning of a 17 kb DNA fragment included the phosphorylation of primers Z37F (sequence above) and TGM23R (5'-GTGATTTGATAGAACAAGGTACGTAAAAGCTGAAAC-3') with T4-polynucleotide kinase (Lucigen Co. Middleton, WI) prior to the PCR amplification reaction. The products of this reaction were extracted from a 1% low-melt Seaplaque agarose gel with QIAEX II gel extraction Kit (QIAGEN) and cloned into pJAZZ-OC vector using the BigEasy v2.0 Linear Cloning Kit (Lucigen Co. Middleton, WI). Multiple size fragments were cloned and verified by sequencing.

### RNA gel-blot analysis and synthesis of DNA probes

RNA (10 μg/sample) was electrophoresed in a 1.2% agarose-3% formaldehyde gel [38]. Size-fractionated RNAs were transferred to Optitran-supported nitrocellulose membrane (Midwest Scientific, Valley Park, MO) by capillary action as described in Sambrook et al., (1989) [38] and cross-linked with UV light (Stratagene, La Jolla, CA). Nitrocellulose RNA blots were prehybridized, hybridized, washed, and exposed to Hyperfilm (Amersham, Arlington Heights, IL) as described by Todd and Vodkin (1996) [39].

Cloned DNAs used as probes were PCR amplified, electrophoresed, and purified from the agarose using the QIAquick gel extraction kit (QUIAGEN, Valencia, CA). DNA concentration of the final eluate was determined with a NanoDrop (NanoDrop Technologies, Inc. Rockland, DE). Purified DNA fragments (25–250 ng) were labeled with [a-^32^P]dATP by random primer reaction [40].

### Sequencing and sequence annotation

A shotgun library of BAC clone 71B1 from *G. max *PI437654 cultivar was constructed at the Keck Center for Comparative and Functional Genomics (University of Illinois). BAC DNA was randomly sheared and cloned into the pCR4Blunt-TOPO vector using the Topo Shotgun Subcloning Kit (Invitrogen). Sequencing of the clones was done using the Big Dye Terminator chemistry (Applied Biosystems, Foster City, CA). Base-calling and quality assessment were performed automatically using PHRED [41]. High-quality sequences were assembled with PHRAP to order the contigs. PCR amplification and direct sequencing of subclones was used to close gaps with an end result of three contigs that could not be overlapped.

Sequencing of all other clones was done at the Keck Center (University of Illinois Biotechnology Center)

Blast searches with sequences of the Tgmt* transposon and the three contigs of 71B1 BAC clones were extended to the 7× draft sequence assembly *Glycine max *0 (Glyma0) from the Joint Genome Initiative (JGI) released 12/06/07. This 7× assembly consisted of 3,317 sequences with a total of 996,173,606 letters which was made accessible to us by the Matt Hudson Laboratory (U. of Illinois at Urbana/Champaign) . We also searched the JGI **Glyma0 **soybean genome assembly using the Phytozome: *Glycine max *(v3.0) BLAST web-site . Note that the scaffolds numbers are predicted to change once the final 8× soybean genome assembly is completed.

### Accession Numbers

Sequence data from this article can be found in the EMBL/GenBank data libraries under accession numbers: [EU190438: *F3'H *(*T*) allele in Williams43]; [EU190439: *F3'H *(T*) allele in XB22A]; [EU190440: *F3'H *(t*) mutant allele in 37609 isoline]; [EU721743: 71B1 BAC clone].

## Authors' contributions

GZ carried out the design of the study, performed and analyzed the results of all the experimental work described including the comparative analysis and drafted the manuscript. LV initiated, coordinated, and led the project and edited the manuscript. Both authors read and approved the final manuscript.

## Supplementary Material

Additional file 1 **Alignment of *Tgm1 *subterminal direct repeats**. The repeated sequences have been organized in this figure starting from the 3'end of the transposon right border. Each direct repeat was read from the 3'-end to the 5'-end. A consensus sequence motif was deduced and is shown boxed. Click here for file

Additional file 2 **Alignment of *Tgm-Express1 *subterminal direct repeats**. The repeated sequences have been organized in this figure starting from the 3'end of the transposon right border. Each sequence repeat was read from the 3'-end to the 5'-end. A consensus sequence motif was deduced and is shown boxed. Click here for file

Additional file 3 **Alignment of *Tgmw4m *subterminal direct repeats**. The repeated sequences have been organized in this figure starting from the 3'end of the transposon right border. Each repeat has been read from the 3'-end to the 5'-end. A consensus sequence motif was deduced and is shown boxed. Click here for file

Additional file 4 Tgmt* and *En-1 *amino acid sequence alignment. The transposase amino acid sequences predicted with Softberry- FGeneSH and aligned with the *MultAlin *program (Corpet, 1988) have 258 aa identities (33%) in the 780 aa at the 5'-end. These regions of both transposases contain the tnp2 domains that map at the same location (highlighted in yellow). Beyond the 780 aa stretch the two proteins diverge considerably with only 141 aa identities (10%), two different conserved domains TNP1 in Tgmt* (highlighted in green) and ptta in *En-1 *(highlighted in blue) that map in different locations, and many gaps that reflect their differences in length. Click here for file

Additional file 5 Alignment of cDNA sequences of clones 43–53 to Tgmt* genomic sequence. The sequences from RT-PCR derived cDNA clones (43, 44, 45, 47 and 53) were aligned to Tgmt* genomic sequence with *MultAlin *program (Corpet, 1988) to reveal the exon-intron junctions and the canonical GT-AG splice boundaries. The exon sequences appear in red and blue depending on the number of clones bearing the exon. Click here for file

Additional file 6 Alignment of Mosaic-cDNA sequences to Tgmt* genomic sequence. The sequences from RT-PCR derived cDNA clones amplified with primers 1 and 7 (Figure 8) (No.:37, 38, 39, 42 and 56) were aligned to Tgmt* genomic sequence with *MultAlin *program (Corpet, 1988). It revealed the splicing site (marked with an asterisk) that does not conform to the canonical GT-AG intron-exon splice boundaries (GA, following the 346 bp of Exon-1 and AT, prior to the 23 bp of Intron-10). The 23 bp of Intron-10 have been highlighted in yellow. The exon sequences appear in red and blue depending on the number of clones bearing the exon. Click here for file

Additional file 7 Tgmt* Gene-1 precursor transcripts sequence. The sequence of a precursor transcript (7,554 bp) expressed by Gene-1 was composed with all 24 exon sequences present in the cDNA clones analyzed (Figure 8 and see Additional file 5: Alignment of cDNA sequences of clones 43–53 to Tgmt* genomic sequence). Click here for file

Additional file 8 Tgmt* Mosaic-transcript sequence. The sequence of the largest (2,572 bp) cDNA clone, No. 37, amplified with primers -1 and -7 contains all 14 exons of the Gene-1 3'-end and the 346 bp (1/9) portion of Exon-1. Purple letters represent Exon-1 bases, highlighted yellow are Intron-10 bases and orange letters are Exons 11–24 bases. Highlighted pink and green are primer 1 and 7 sequences respectively. Click here for file

Additional file 9 Tgmt* genomic sequence (20,544 bp). The reverse complement of Tgmt* genomic sequence (20,544 bp) is shown. Exons are in orange letters. Purple letters are the portion of Exon-1 that splices with Intron-10 to form the mosaic-transcripts. The 23 bases of Intron-10 that are part of Mosaic transcripts are shown in yellow. Also in yellow are the bases of Intron-19 and -20 that are not spliced out in cDNA clone 47. The direct and reverse subterminal repeats are highlighted in yellow and green respectively. The 13 bp CACTA terminal inverted repeats are highlighted in blue. Sequences of Primers 1, 3 and 5 are highlighted in pink and those of primers 2, 4, 6 and 7 are highlighted in green. Click here for file

Additional file 10 Alignment of sequences of 3 contigs from 71B1 BAC clone in the 7× draft sequence assembly (JGI). Click here for file
