# Supplementary material for: A putative autonomous 20.5 kb-CACTA transposon insertion in an F3'H allele identifies a new CACTA transposon subfamily in Glycine max
Source: BMC Plant Biol. 2008 Dec 2;8:124. doi: 10.1186/1471-2229-8-124 (PMC2613891; doi:10.1186/1471-2229-8-124)
Supplement: Additional file 2 — Alignment of Tgm-Express1 subterminal direct repeats. The repeated sequences have been organized in this figure starting from the 3'end of the transposon right border. Each sequence repeat was read from the 3'-end to the 5'-end. A consensus sequence motif was deduced and is shown boxed. [file 1471-2229-8-124-S2.pdf]

Alignment of *Tgm-Express1* subterminal direct repeats

Right border	T T A G C A G C A T C T T G C
	T T G G C A G A A A C A T
	T T G G C A G A A T C A T A
	T T G A C A G A A T T T T T C A
Consensus	**T T G G C A G A A T C T T A C A G**
	T T G G C A G C A T C T T T C A
	T T G G C A G A A T C A T A C A G
Left border	T T G G C G G A A T C T T A C A

Additional file 2
