# Supplementary material for: A putative autonomous 20.5 kb-CACTA transposon insertion in an F3'H allele identifies a new CACTA transposon subfamily in Glycine max
Source: BMC Plant Biol. 2008 Dec 2;8:124. doi: 10.1186/1471-2229-8-124 (PMC2613891; doi:10.1186/1471-2229-8-124)
Supplement: Additional file 3 — Alignment of Tgmw4m subterminal direct repeats. The repeated sequences have been organized in this figure starting from the 3'end of the transposon right border. Each repeat has been read from the 3'-end to the 5'-end. A consensus sequence motif was deduced and is shown boxed. [file 1471-2229-8-124-S3.pdf]

Alignment of *Tgmw4m* subterminal direct repeats

Right border	T T G G C A G T A T C G T
	T T G G C A G A A A T T T C T A
	T T G G C A G A A T T T T A A
	T T G G C A A T A T T A T T
	T T G G C A G A A T T T T T C A
	T T G G C A G A A T C T T T C A
	T T G G C A G A A T C T T T C A
	T T G G T A G A A T C A T A C A G
	T T G G C G G T A T C T T T C A
	T T G G C A G A A T C A T A C A G
Consensus	T T G G C A G A A T C T T A C A G
	T T G T C A G A A T C A T A
	T T G G C A G A A T C T T A

Additional file 3
