# Supplementary material for: A putative autonomous 20.5 kb-CACTA transposon insertion in an F3'H allele identifies a new CACTA transposon subfamily in Glycine max
Source: BMC Plant Biol. 2008 Dec 2;8:124. doi: 10.1186/1471-2229-8-124 (PMC2613891; doi:10.1186/1471-2229-8-124)
Supplement: Additional file 4 — Tgmt* and En-1 amino acid sequence alignment. The transposase amino acid sequences predicted with Softberry- FGeneSH and aligned with the MultAlin program (Corpet, 1988) have 258 aa identities (33%) in the 780 aa at the 5'-end. These regions of both transposases contain the tnp2 domains that map at the same location (highlighted in yellow). Beyond the 780 aa stretch the two proteins diverge considerably with only 141 aa identities (10%), two different conserved domains TNP1 in Tgmt* (highlighted in green) and ptta in En-1 (highlighted in blue) that map in different locations, and many gaps that reflect their differences in length. [file 1471-2229-8-124-S4.pdf]

Additional file 4: *Tgmt** and *En-1* amino acid sequence alignment

```
           1                                                                                                                      130
    Tgmt*  MDRHWMKTAR ITEEYENGVE GFLKFAKDNA SDNGGLYFCP CVKCLNGRRQ CLDDIRTHLI CDGICPTYTK WIWHGELPEM SSTPPTAPTD --EQVGDQIE DMLRDLGQEG FRQANAPYYD TLHNDSKIPL
     En-1  MYDGF--DSV THGHSDAWLR VADEFVALAF VGDARLARCP CIKCRNLVRL KKVELSYHIF KHGFMPNYLV WHEHGEVDHT IESDGDQDID RMEEMLDDIR NEYPDLQNNQ AFPEDVREFY KLLEASE-AK
Consensus  Mddgf..dar iheesdagle gadeFaadaa sddagLafCP CiKClNgrRl ckddirtHif cdGfcPnYlk WheHGEldem iesdgdadiD ..E"mgDdIe delpDLgneg afpadapefd kLheaSe.ak

           131                                                                                                                    260
    Tgmt*  FIGCTKYTRL SGVLALVNLK ARFGWSDKSF NELLLLLKNM LPGDNTLPKT HYEAKKILCP VGMEYQKIHA CRNDCILYRH EFAELRNCPT CGVSRYKVGS GASSEAGSTY IDRPAKVCWY LPVIPRFKRL
     En-1  VHEGTNVSVL QVVTRLMAMK SKYTFSNKCY NDIVKLIIDI SPPNHNMPKD LYHCKKLVAG LGMNYQKIDA CEDNCMLFWK EHENTTHCIH CSKSRYAVVL DEDGNEVTTK V--PIKQLRY MPITPRLKRL
Consensus  fhecTkvsrL qgVlaLmalK akfgfSdKcf NdilkLiidi lPgdhnlPKd hYeaKKilag lGMeYQKIdA CeddCiLfrh EfaelrhCih CgkSRYaVgl dadgeagsTk i..PaKqcrY lPiiPRfKRL

                                             ---------tnp2---------
           261                                                                                                                    390
    Tgmt*  FANAEDAKNL TWHVDGRTKD ---GLLRHPA DSPQWKKVDQ LYPVFAEDPR NLRVGLASDG MNPFRSLSCN HSSWPVLLII YNLPPWLCIK RKYIMMSMMI AGPRQPGNDI DVYLAPLIED LTKLWVEGVD
     En-1  FLNQETAKQM RWHKEGDRQG QDPDVMVHPS DGEAWQALDR FDPEFARDPR SVRLGLSTDG FTPYSNNSTS YSCWPVFMMP YNLPPNKCMK EEVMFLALIV PGPKDPVTKI NVFMEPLIEE LKMLW-QGVE
Consensus  FaNaEdAKnl rWHkdGdrkd ...dllrHPa DgeaWkalDq fdPeFAeDPR nlRlGLasDG fnPfrnlScn hScWPVflii YNLPPnkCiK eeviflalii aGPkdPgndI dVflaPLIEd LkkLW."GVd

           391                                                                                                                    520
    Tgmt*  VYDGNAHESF RLRAMIFCTI NDFPAYGNLS GYSVKGHLAC PICEKDTTYL QLKHGKK-TV YTRHRRFLQP FHPYRRLKKA FD-GTSENDS ASIPLSGVEV FDRVKNICNI YGKTQKKDGA PKNIWKKRSI
     En-1  AYDSHLKCCF TLRAAYLWSI HDLLAYGIFS GWCVHGILRC PICMGDSQAY RLEHGKKETF FDVHRRLLPY NHPFRKDTKS FRKGKRVRDG PPKRQTGENI MRQHRDLKPG VGGRFQGYGK -EHNWTHISF
Consensus  aYDghahccF rLRAaifcsI hDflAYGifS GwcVhGhLaC PICegDsqal qLeHGKK.Tf fdrHRRfLpp fHPfRkdkKa Fd.GkrenDg apiplsGeei fdqhkdicng vGgrfkgdGa .ehiWkhiSf

           521                                                                                                                    650
    Tgmt*  FFDLPYWCNL DVRHCLDVMH VEKNVCDSLV GTLLNIKGKT KDGLKCRQDL VEMGVRHQLH PVSKGLRT-Y LPPACHTMST YEKKSFCHCL KNVKVPQGYS SNIKSLVSVD EMKLVGLKSH DCHVLMQQLL
     En-1  IWELPYTKAL LLPHNIDLMH QERNVAESII SMCFDFTGQT KDNMNARRDL AELCDRPHLE LRKNPSGSES RPQAPYCLKR QEREEIFQWL KKLRFPDRYA ANIKRAVNLD TGKLVGLKSH DYHILIERLV
Consensus  ffdLPYtcaL dlpHciDlMH qEkNVadSii gmcfdfkGkT KDglkaRqDL aElcdRhhLe lrkkglgs.s lPpAchclkr qEkeefchcL KklkfPdgYa aNIKraVnlD egKLVGLKSH DcHiLi"qLl

           651                                                                                                                    780
    Tgmt*  PVAIRGILPD KVRVAITRLC FFFNAICSKV IDPKQLDDLE NEAAIIICQL EMYFPPTFFD IMIHLLVHLV REIRLCGPVY LRWMYPVERY MKVLKSYTKN QYRPEASIVE RYVAEEAIEF CSTYIEDASP
     En-1  PVMFRGYFSP DVWKIFAELS YFYKQICAKE ISKKLMLRFE KEIVVLVCKM EKVFPPGFFN CMQHLLVHLP WEALVGGPAQ FRWMYSQERE LKKLRGMVRN KARVEGCIAE AFAAREITLF SSKYFSDTNN
Consensus  PVafRGifpd dVrkafaeLc fFfkaICaKe IdkKllddfE kEaaiiiCkl EkvFPPgFFd cMiHLLVHLp rEallcGPaq fRWMYpqERe lKkLkgmtkN kaRpEacIaE afaAeEaieF cSkYfeDann

           781                                                                                                                    910
    Tgmt*  VGIPESRHEA TRQGRGTRGF NVVTMDRQKL SQAHLYVLNN TAEVIPYIDA HKEYVAASHP NMNMMRVLQE HNRSFINWFR NTIFASDSAS KTLSLLAVGP NLNVLTWKGY DINNYSFYTK SQDDKSTVQN
     En-1  VNAQTTRYHV AEQAPIT-DL SAFKWDGKGV GAYTSHLVGT IER------- ---------- ---------- ---------- ---------N KTLLFLYV-- --NMPELHPY FQIFDSIYKP NKQLTQKQLD
Consensus  VgapesRhea aeQapgT.df nafkmDgkgl gaahlhllgn iae....... .......... .......... .......... .........n KTLlfLaV.. ..NmlelhgY diifdSfYkk nkddkqkqld

           911                                                                                                                    1040
    Tgmt*  SGVMIDAHSD HFSRASDNNP IRASMAYYGV ITDIWELDYG EFRVPVFKCQ WVNGNVGVRQ DKLGFTLVDL QRIGYKDEPF IMAAQARQVF YVEDPSDSTW SVVLQGKTSG IPADTDQATL DVNEIPTFAQ
     En-1  DLRLKGLHGG PSFVQWFHEH CKKPEAS--V SKDLLQISHG QLTARKFDRY DINGY-RFRT AKLEASRPLA ATTNSGVVAS SYSDDGQLED Y--------- YGIVQDITEY TFGGHKPLRL VTFDCIWFDP
Consensus  dgrlidaHgd hffrasdheh ckapeAs..V ikDil"idhG "frapkFdcq diNGn.gfRq aKLeaslpda arigsgdeaf imaadaqled Y......... sgilQdiTeg ifadhdparL dtfdcitFap

           1041                                                                                                                   1170
    Tgmt*  QMPSINAEND DDDV-YANRI DHDEEPMATP PTSPPPPTSP PPADSPSAIS KPKTRQATRL RKLTARTLDQ PRPIVNVNPV TGRGSGSEKD KFHSYLGVVA REKIPIVHSS WKVVPESLKN IVWNDILGKF
     En-1  QVGTRVDEFG MVEVKHASRY KGNEYNNIIL AHQAHQVYYL SYPHKSFKTW WVAYKVNPEV HPYRYQNYNL STNDDDEDDV VFQEVGDQAD DSDNDSIVSE GAGLNELASL TVELMEEPIS SNSKRQRLEE
Consensus  QmgsinaEfd dddV.hAnRi dgdEenmail ahqahpptsl ppadkpfais kpatkqapel hklraqnldl prndddeddV tfqesGd"aD dfdndlgVsa gaginelaSl tkelmEelin inskdilgee

           1171                                                                                                                   1300
    Tgmt*  DIPEGTAAKK KVMSTVATRW RQFKSSLTSR YIYAEKHGED NPDAASKYGM EQQTWEQFAK SRQTPTWQGI RKKAQEIQKF NDSPHLLSRG GYELMEKKLM EEKMKTRQRQ AECTENTPMV VDPPSPIARH
     En-1  TVLE----TQ QWMFRMDSSG RRSRSRRSRG SSGAPNMFEG TTTSRSRQEQ LLASLEQMRG SSGPSNTEGT TSRAADLVAP ---------- ---------- -----TMAPT AEAAVDAEAA VD--------
Consensus  dilE....kk kvMfrmasrg RqfkSrlsrg sigAekhfEd npdaaSkqem elaslEQfag Srgppnt"Gi rkkAadiqaf .......... .......... .....Tmapq AEaaedaeaa VD........

           1301                                                                                                                   1430
    Tgmt*  VKWKMARTNK YGKMTSAAAQ QISDKIDELE EQSTQGTFVP HGRNDILNTA LGREEHPGRV LAAGHGVTIS SYFGQRSSAS NSSAATITPD QLVQIIGNLK QEWTKEVEDA SKQKMDMLQK ELDAIKTELS
     En-1  ---------- ------AEAE EAAAELDDGE ETS------- ---------- ---GADASTE EAATQAPPRR AIRYRRSLTL KPS---KPFD QRRVIEPKGT RAWKEVSWDG TGHRTPIL-- ----------
Consensus  .......... ......AaA" "aaaeiDdgE EqS....... .......... ...eadagre eAAghappir aifgqRSlal kpS...ipfD QlrqIegkgk qaWkeeseDa sghkmdiL.. ..........
```

```
                                                                               ---------TNP1---------
           1431                                                                                                          1560
    Tgmt*  QMQTQQSAPV QPANPNVLIA RVSTKESCAE AVANVVAGDP SAVEENTMGL YVVCGDSKQL VALGKVYQVG GMIHNVPYAD EVVRVSVITV YDGDARVPIP TPEIEYVREA MNTFIGWPTN LVKPFSADSN
     En-1  ---TELGICL RFAYPAMVTE GGQEIAAHYW AHWDLKPYG- NDGTHTSKVW DLFWGQFRVC DPY------- --TLDDSYVR EVFNGSADRA VKGMMYKARL RAVTVYQKRQ GNYCDANMAK EIHLTAQQYK
Consensus  ...T"lgacl qfAnPamlia ggqeiaacae Ahadlkagd. nageenskgl dlfcGdfkqc dal....... ..ihddpYad EVfngSadra vdGdarkail raeieYqkea gNtcdanmak eihlfaadsk

                     ---------ptta---------
           1561                                                                                                          1690
    Tgmt*  QDVRNPKGHV DRSNAGDAMD PLGEIMKILY EVYMNPVELP WEASRFGIPN IDAKFYITHA DMAEIISGHK CLNISILQLW MMYLDECATS RGDGSVYGFL EPQSIHIGKE DRQQCQLYIE TWVKESQRCL
     En-1  ESEVDWLSH- -HSDAWAWMC EYWASEEFLA ISNRNRM--- ---NRLSKPG VHFFGADGHV GKAARMAARN GVEPTLLQV- ---------- ----FVEGHK GPDPNHPEI- ---------- ----------
Consensus  "derdpkgH. .hSdAgaaMc elgaieefLa esnmNpm... ...nRfgiPg idaffadgHa dkAaiiaahk cleisiLQl. .......... ....fVeGfk ePdpiHiei. .......... ..........

           1691                                                                                                          1820
    Tgmt*  YLGAYLHQSH WQLFVLCPRE NMVVWFCSLR KKPDVNIKAV INSAMKTISS SLEGMSQQGP PRWIEPKSHV QSGGYECGYY VMHWMWCIVS GRLKDDWNRP CKRMLLFDLL AQEKQWLQRL WQLKPEEKQY
     En-1  ---------- --LNDSNATE KLARYIDNVR EKNGPDT-DW LTGEFDTEAA YKAGGG---- ---------V PHGRLAIGDG VVPRRSYTRR SNFSAGSNRP RRP------- ---------- ----------
Consensus  .......... ..LfdlcarE klarwfcnlR eKndpdi.av ingafdTeaa skaGgg.... .........V phGglacGdg Vmhrmscirr gnfkadsNRP ckp....... .......... ..........

           1821                                                                                                          1950
    Tgmt*  PNSDTDEDVL QIFFKERELN GDFISRASDL LWRRDFRSSG DYDISELTDN TSQQIEQIIE TDSDGGLLKL TRTQEWLTGD NSPPINKKVT AKLKRELLLL SVGIGLACSG YCLVIFSVQV ETWQDIFAGG
     En-1  ---------- ---------- ---------- ---------- ---------- ---------- SAREGELLEK MTQMEESMAQ YKQQVQQQMQ QMQNWMLHQM YGGAG----- ---------- ----------
Consensus  .......... .......... .......... .......... .......... .......... sardGeLLek mrqmEelmad nkppinkkmq aklkreLhll sgGaG..... .......... ..........

           1951                                                                                                          2080
    Tgmt*  INDSDIISNL GMEMNHKQKM DVRTNSLVRP SGTIDKDKEK LRIANNGVVQ SEEQTLPIGG DGWEKSKMKK KRSCIKLDVS PSTTLTKPVN TFQETKQGMQ QRLATDSRFF QAKIIFYGKI RKSVVCNGTI
     En-1  -------TQF GMPPFQQPPI ITHPVSGQSS DRSTAAADGS QGSATSVQDQ L----MPLGV IGGQMMPWAP RQPGIWPPMQ TQMPPPMPW- ---------- ---------- ---------- ----------
Consensus  .......snf GMemfhkpki dthpnSgqrp dgsiaaadek lgiAnngqdQ l....lPiGg dGg"kmkmak kqpcIkldmq pqmplpkPv. .......... .......... .......... ..........

           2081                                                                                                   2202
    Tgmt*  GVGKSDGISQ QTGLGIRAST PRNNQDNNSL VNDRRGRPVS SDKERVNFRV VNKATARDEF NSSSPTSSAK INTAICVMIF FISSIACNQV FSDLERSVCR MVHRQVATIA WLEADSVCGS HS
     En-1  ---------- --GFPPRGQS QSPGLPSHSP GSGSGSHHAS PPPDQSTFMD LLMNTSGGGS NDPPTE---- ---------- ---------- ---------- ---------- ---------- --
Consensus  .......... ..GfgiRaqs prngldnhSl gndrgghhaS pdkdqsnFmd llkaTagdef Ndpppe.... .......... .......... .......... .......... .......... ..
```
