# Supplementary material for: A putative autonomous 20.5 kb-CACTA transposon insertion in an F3'H allele identifies a new CACTA transposon subfamily in Glycine max
Source: BMC Plant Biol. 2008 Dec 2;8:124. doi: 10.1186/1471-2229-8-124 (PMC2613891; doi:10.1186/1471-2229-8-124)
Supplement: Additional file 5 — Alignment of cDNA sequences of clones 43–53 to Tgmt* genomic sequence. The sequences from RT-PCR derived cDNA clones (43, 44, 45, 47 and 53) were aligned to Tgmt* genomic sequence with MultAlin program (Corpet, 1988) to reveal the exon-intron junctions and the canonical GT-AG splice boundaries. The exon sequences appear in red and blue depending on the number of clones bearing the exon. [file 1471-2229-8-124-S5.pdf]

Additional file 5: Alignment of cDNA sequences of clones 43-53 to *Tgmt** genomic sequence

```
          1                                                                                                                         130
     gDNA GGCTGAAATA ATATCAGGTC ACAAGTGTTT AAACATTTCT ATACTGCAAC TATGGATGAT GTAAGTCATT TAATTACAAC CTTTAACCCT AAATGTTATC ATAAGCAACA AATTGTAAAT GTAATACATT
     Cl47 GGCTGAAATA ATATCAGGTC ACAAGTGTTT AAACATTTCT ATACTGCAAC TATGGATGAT ---------- ---------- ---------- ---------- ---------- ---------- ----------
     Cl44 GGCTGAAATA ATATCAGGTC ACAAGTGTTT AAACATTTCT ATACTGCAAC TATGGATGAT ---------- ---------- ---------- ---------- ---------- ---------- ----------
     Cl45 GGCTGAAATA ATATCAGGTC ACAAGTGTTT AAACATTTCT ATACTGCAAC TATGGATGAT ---------- ---------- ---------- ---------- ---------- ---------- ----------
     Cl53 GGCTGAAATA ATATCAGGTC ACAAGTGTTT AAACATTTCT ATACTGCAAC TATGGATGAT ---------- ---------- ---------- ---------- ---------- ---------- ----------
     Cl43 GGCTGAAATA ATATCAGGTC ACAAGTGTTT AAACATTTCT ATACTGCAAC TATGGATGAT ---------- ---------- ---------- ---------- ---------- ---------- ----------
Consensus GGCTGAAATA ATATCAGGTC ACAAGTGTTT AAACATTTCT ATACTGCAAC TATGGATGAT .......... .......... .......... .......... .......... .......... ..........

          131                                                                                                                       260
     gDNA TGTGGTATAA TGATTGTATT GTCTTTCAAA TAGGTATTTG GATGAGTGTG CTACAAGCAG AGGTGATGGC TCAGTGTATG GCTTCCTTGA GCCTCAATCA ATACACATTG GTAAGGAGGA CCGTCAACAA
     Cl47 ---------- ---------- ---------- ---GTATTTG GATGAGTGTG CTACAAGCAG AGGTGATGGC TCAGTGTATG GCTTCCTTGA GCCTCAATCA ATACACATTG GTAAGGAGGA CCGTCAACAA
     Cl44 ---------- ---------- ---------- ---GTATTTG GATGAGTGTG CTACAAGCAG AGGTGATGGC TCAGTGTATG GCTTCCTTGA GCCTCAATCA ATACACATTG GTAAGGAGGA CCGTCAACAA
     Cl45 ---------- ---------- ---------- ---GTATTTG GATGAGTGTG CTACAAGCAG AGGTGATGGC TCAGTGTATG GCTTCCTTGA GCCTCAATCA ATACACATTG GTAAGGAGGA CCGTCAACAA
     Cl53 ---------- ---------- ---------- ---GTATTTG GATGAGTGTG CTACAAGCAG AGGTGATGGC TCAGTGTATG GCTTCCTTGA GCCTCAATCA ATACACATTG GTAAGGAGGA CCGTCAACAA
     Cl43 ---------- ---------- ---------- ---GTATTTG GATGAGTGTG CTACAAGCAG AGGTGATGGC TCAGTGTATG GCTTCCTTGA GCCTCAATCA ATACACATTG GTAAGGAGGA CCGTCAACAA
Consensus .......... .......... .......... ...GTATTTG GATGAGTGTG CTACAAGCAG AGGTGATGGC TCAGTGTATG GCTTCCTTGA GCCTCAATCA ATACACATTG GTAAGGAGGA CCGTCAACAA

          261                                                                                                                       390
     gDNA TGTCAACTTT ATATTGAGAC ATGGGTGAAG GAATCACAAC GATGCTTGTA CTTAGGAGCA TACTTGCATC AGTAAGTTAA ATTTTTTTGT GGCATTTAAC AAATGTTATG ATTTCTAAAC TTGCTAATTA
     Cl47 TGTCAACTTT ATATTGAGAC ATGGGTGAAG GAATCACAAC GATGCTTGTA CTTAGGAGCA TACTTGCATC A--------- ---------- ---------- ---------- ---------- ----------
     Cl44 TGTCAACTTT ATATTGAGAC ATGGGTGAAG GAACCACAAC GATGCTTGTA CTCAGGAGCA TACTTGCATC A--------- ---------- ---------- ---------- ---------- ----------
     Cl45 TGTCAACTTT ATATTGAGAC ATGGGTGAAG GAATCACAAC GATGCTTGTG CTTAGGAGCA TACTTGCATC A--------- ---------- ---------- ---------- ---------- ----------
     Cl53 TGTCAACTTT ATATTGAGAC ATGGGTGAAG GAATCACAAC GATGCTTGTA CTTAGGAGCA TACTTGCATC A--------- ---------- ---------- ---------- ---------- ----------
     Cl43 TGTCAACTTT ATATTGAGAC ATGGGTGAAG GAATCACAAC GATGCTTGTA CTTAGGAGCA TACTTGCATC A--------- ---------- ---------- ---------- ---------- ----------
Consensus TGTCAACTTT ATATTGAGAC ATGGGTGAAG GAAtCACAAC GATGCTTGTa CTtAGGAGCA TACTTGCATC A......... .......... .......... .......... .......... ..........

          391                                                                                                                       520
     gDNA TAATCATCAA CTTCAGGTCA CATTGGCAAC TATTTGTTCT CTGTCCTAGG GAAAACATGG TTGTTTGGTT TTGTTCGTTG CGAAAGAAGC CTGATGTTAA CATAAAAGCC GTAATAAATA GGTACGAGTG
     Cl47 ---------- ------GTCA CATTGGCAAC TATTTGTTCT CTGTCCTAGG GAAAACATGG TTGTTTGGTT TTGTTCGTTG CGAAAGAAGC CTGATGTTAA CATAAAAGCC GTAATAAATA G---------
     Cl44 ---------- ------GTCA CATTGGCAAC TATTTGTTCT CTGTCCTAGG GAAAACATGG TTGTTTGGTT TTGTTCGTTG CGAAAGAAGC CTGATGTTAA CATAAAAGCC GTAATAAATA G---------
     Cl45 ---------- ------GTCA CATTGGCAAC TATTTGTTCT CTGTCCTAGG GAAAACATGG TTGTTTTGTT TTGTTCGTTG CGAAAGAAGC CTGATGTTAA CATAAAAGCC GTAATAAATA G---------
     Cl53 ---------- ------GTCA CATTGGCAAC TATTTGTTCT CTGTCCTAGG GAAAACATGG TTGTTTGGTT TTGTTCGTTG CGAAAGAAGC CTGATGTTAA CATAAAAGCC GTAATAAATA G---------
     Cl43 ---------- ------GTCA CATTGGCAAC TATTTGTTCT CTGTCCTAGG GAAAACATGG TTGTTTGGTT TTGTTCGTTG CGAAAGAAGC CTGATGTTAA CATAAAAGCC GTAATAAATA G---------
Consensus .......... ......GTCA CATTGGCAAC TATTTGTTCT CTGTCCTAGG GAAAACATGG TTGTTTgGTT TTGTTCGTTG CGAAAGAAGC CTGATGTTAA CATAAAAGCC GTAATAAATA G.........

          521                                                                                                                       650
     gDNA TAATGTACTT AAAGATAGCC GAGCTGTCAT TGTTGTTGAA TATGCAGCGT AAATGTTTGT TATAAGTAAC ATCATATATG ATTTCGTTTA CTAGTGCAAT GAAGACAATA AGTAGTTCTT TGGAAGGCAT
     Cl47 ---------- ---------- ---------- ---------- ---------- ---------- ---------- ---------- ---------- ----TGCAAT GAAGACAATA AGTAGTTCTT TGGAAGGCAT
     Cl44 ---------- ---------- ---------- ---------- ---------- ---------- ---------- ---------- ---------- ----TGCAAT GAAGACAATA AGTAGTTCTT TGGAAGGCAT
     Cl45 ---------- ---------- ---------- ---------- ---------- ---------- ---------- ---------- ---------- ----TGCAAT GAAGACAATA AGTAGTTCTT TGGAAGGCAT
Consensus .......... .......... .......... .......... .......... .......... .......... .......... .......... ....tgcaat gaagacaata agtagttctt tggaaggcat

          651                                                                                                                       780
     gDNA GTCTCAGCAA GGTCCACCTC GGTGGATTGA ACCCAAGGTT AGATGGTTGT TTAGATGAAC CCCTATGTAA TTTTTAAAGG GTACAAGTGA TAATAGTTAT TTTCACTGAT AAATATAGAG TCATGTTCAA
     Cl47 GTCTCAGCAA GGTCCACCTC GGTGGATTGA ACCCAAG--- ---------- ---------- ---------- ---------- ---------- ---------- ---------- --------AG TCATGTTCAA
     Cl44 GTCTCAGCAA GGTCCACCTC GGTGGATTGA ACCCAAG--- ---------- ---------- ---------- ---------- ---------- ---------- ---------- --------AG TCATGTTCAA
     Cl45 GTCTCAGCAA GGTCCACCTC GGTGGATTGA ACCCAAG--- ---------- ---------- ---------- ---------- ---------- ---------- ---------- --------AG TCATGTTCAA
Consensus gtctcagcaa ggtccacctc ggtggattga acccaag... .......... .......... .......... .......... .......... .......... .......... ........ag tcatgttcaa

          781                                                                                                                       910
     gDNA AGTGGAGGGT ACGAGTGTGG ATACTATGTG ATGCATTGGA TGTGGTGCAT CGTTAGTGGT CGTTTGAAGG ATGACTGGAA CAGGGTATAT ATACAAAGTC TAATTTCAAT TTTCATGTTA GTTGATATTT
     Cl47 AGTGGAGGGT ACGAGTGTGG ATACTATGTG ATGCATTGGA TGTGGTGCAT CGTTAGTGGT CGTTTGAAGG ATGACTGGAA CAGG------ ---------- ---------- ---------- ----------
     Cl44 AGTGGAGGGT ACGAGTGTGG GTACTATGTG ATGCATTGGA TGTGGTGCAT CGTTAGTGGT CGTTTGAAGG ATGACTGGAA CAGG------ ---------- ---------- ---------- ----------
     Cl45 AGTGGAGGGT ACGAGTGTGG ATACTATGTG ATGCATTGGA TGTGGTGCAT CGTTAGTGGT CGTTTGAAGG ATGACTGGAA CAGG------ ---------- ---------- ---------- ----------
Consensus agtggagggt acgagtgtgg atactatgtg atgcattgga tgtggtgcat cgttagtggt cgtttgaagg atgactggaa cagg...... .......... .......... .......... ..........

          911                                                                                                                       1040
     gDNA GTTTAATTAT TAATAATGTC TTCCATTGTT AATTTTGTAG TGGTTCTCGG ATGGATCAGC ATTAGATGTG GAGGCCATGA CAATAATTCG AAAGAATTGG GCAACTTACT TTTTAGCTAT TAGAAATAAC
     Cl47 ---------- ---------- ---------- ---------- TGGTTCTCGG ATGGATCAGC ATTAGATGTG GAGGCCATGA CGATAATTCG AAAGAATTGG GCAACTTACT TTTTAGCTAT TAGAAATAAC
     Cl44 ---------- ---------- ---------- ---------- TGGTTCTCGG ATGGATCAGC ATTAGATGTG GAGGCCATGA CAATAAGTCG AAAGAATTGG GCAGCTTACT TTTTAGCTAT TAGAAATAAC
     Cl45 ---------- ---------- ---------- ---------- TGGTTCTCGG ATGGATCAGC ATTAGATGTG GAGGCCATGA CAATAATTCG AAAGAATTGG GCAACTTACT TTTTAGCTAT TAGAAATAAC
Consensus .......... .......... .......... .......... tggttctcgg atggatcagc attagatgtg gaggccatga caataattcg aaagaattgg gcaacttact ttttagctat tagaaataac
```

```
           1041                                                                                                                                         1170
     gDNA  AGATGCTAAA TATGATGTAG ATTATTATGA ATGACTACAT TTTCCTTTAA TGACACCCTT TAGTGGTATA TTTTAATGAA TTGTTTCATG TCACATTAAT GTTTTTTAAA AACCTACGTA ATGGTTTAGT
     Cl47  AGATGCTAAA TATGATGTAG ATTATTATGA ATGACTACAT CTTCCTTTAA TGACACCCTT TAGTGGTATA TTTTAATGAA TTGTTTCATG TCACATTAAT GTTTTTTAAA AACCTACGTA ATGGTTTAGT
     Cl44  AGATGCTAAA TATGATGTAG ATTATTATGA ATGACTACAT TTTCCTTTAA TGACACCCTT TAGTGGTATA TTTTAATGAA TTGTTTCATG TCACATTAAT GTTTTTTAAA AACCTACGTA ATGGTTTAGT
     Cl45  AGATGCTAAA TATGATGTAG ATTATTATGA ATGACTACAT TTTCCTTTAA TGACACCCTT TAGTGGTATA TTTTAATGAA TTGTTTCATG TCACATTAAT GTTTTTTAAA AACCTACGTA ATGGTTTAGT
Consensus  agatgctaaa tatgatgtag attattatga atgactacat tttcctttaa tgacaccctt tagtggtata ttttaatgaa ttgtttcatg tcacattaat gttttttaaa aacctacgta atggtttagt

           1171                                                                                                                                         1300
     gDNA  AAGGAGTTTA TGTATTCTGA AATTGTTTTG GTTTGTTGTA GTCTCGTATT AGAAAATATA TATTTTGATA GGATGAGTGC AGTTTTCTTA GAAATTGTAC TCCTACTATG TATGTACTGC ACAGTTGCAC
     Cl47  AAGGAGTTTA TGTATTCTGA AATTGTTTTG GTTTGTTGTA GTCTCGTATT AGAAAATATA TATCTTGATA GGATGAGTGC AGTTTTCTTA GAAATTGTAC TCCTACTATG TATGTACTGC ACAGTTGCAC
     Cl44  AAGGAGTTTA TGTATTCTGA AATTGTTTTG GTTTGTTGTA GTCTCGTATT AGAAAATATA TATTTTGATA GGATGAGTGC AGTTTTCTTA GAAATTGTAC TCCTACTATG TATGTACTGC ACAGTTGCAC
     Cl45  AAGGAGTTTA TGTATTCTGA AATTGTTTTG GTTTGTTGTA GTCTCGTATT AGAAAATATA TATTTTGATA GGATGAGTGC AGTTTTCTTA GAAATTGTAC TCCTACTATG TATGTACTGC ACAGTTGCAC
Consensus  aaggagttta tgtattctga aattgttttg gtttgttgta gtctcgtatt agaaaatata tattttgata ggatgagtgc agttttctta gaaattgtac tcctactatg tatgtactgc acagttgcac

           1301                                                                                                                                         1430
     gDNA  CTTCCATCAA CTTAAC-AAC ACGTAGTTAC TTGTGATAGC ACTTGTATTA TTTTTATTCC CATTACCGTA AGGCCTGAAG TGGTAAGTAG ATAATGCCCT GCGTGTAGCA CATAATTTCT ACTTTGGGTA
     Cl47  CTTCCATCAA CTTAAC-AAC ACGTAGTTAC TTGTGATAGC ACTTGTATTA TTTTTATTCC CATTACCGTA AGGCCTGAAG TGGTAAGTAG ATAATGCCCG GCGTGTAGCA CATAATTTCT ACTTTGGGTA
     Cl44  CTTTCATCAA CTTAAC-AAC ACGTAGTTAC TTGTGATAGC ACTTGTATTA TTTTTATTCC CATTACCGTA AGGCCTGAAG TGGTAAGTAG ATAATGCCCT GCGTGTAGCA CATAATTTCT ACTTTGGGTA
     Cl45  CTTCCATCAA CTTAACCAAC ACGTAGTTAC TTGTGATAGC ACTTGTATTA TTTTTATTCC CATTACCGTA AGGCCTGAAG TGGTAAGTAG ATAATGCCCT GCGTGTAGCA CATAATTTCT ACTTTGGGTA
Consensus  cttccatcaa cttaac.aac acgtagttac ttgtgatagc acttgtatta tttttattcc cattaccgta aggcctgaag tggtaagtag ataatgccct gcgtgtagca cataatttct actttgggta

           1431                                                                                                                                         1560
     gDNA  TAGCCTTGCA AGCGAATGTT GTTGTTTGAC CTTCTAGCAC AGGAAAAACA ATGGTTGCAA AGGCTGTGGC AACTGAAGCC GGAGGAAAAG CAATATCCCA ATAGGTATGT TAACAGATTA TTTTAACTCT
     Cl47  TAGCCTTGCA AGCGAATGTT GTTGTTTGAC CTTCTAGCAC AGGAAAAACA ATGGTTGCAA AGGCTGTGGC AACTGAAGCC GGAGGAAAAG CAATATCCCA ATAG------ ---------- ----------
     Cl44  TAGCCTTGCA AGCGAATGTT GTTGTTTGAC CTTCTAGCAC AGGAAAAACA ATGGTTGCAA AGGCTGTGGC AACTGAAGCC GGAGGAAAAG CAATATCCCA ATAG------ ---------- ----------
     Cl45  TAGCCTTGCA AGCGAATGTT GCTGTTTGAC CTTCTAGCAC AGGAAAAACA ATGGTTGCAA AGGCTGTGGC AACTGAAGCC GGAGGAAAAG CAATATCCCA ATAG------ ---------- ----------
Consensus  tagccttgca agcgaatgtt gttgtttgac cttctagcac aggaaaaaca atggttgcaa aggctgtggc aactgaagcc ggaggaaaag caatatccca atag...... .......... ..........

           1561                                                                                                                                         1690
     gDNA  TTGAAATGTT TGCAGTCAGC TGAGTTGACT TCCATGTCAA TGTAATCATG TTCTGCAGGG TCACATGCAA AGAGTGTAGT AGCCATTTCA GCCTCTGAGG TACACAACAT AGACTTTATA CTCTTTATAA
     Cl47  ---------- ---------- ---------- ---------- ---------- --------GG TCACATGCAA AGAGTGTAGT AGCCATTTCA GCCTCTGAG- ---------- ---------- ----------
     Cl44  ---------- ---------- ---------- ---------- ---------- --------GG TCACATGCAA AGAGTGTAGT AGCCATTTCA GCCTCTGAG- ---------- ---------- ----------
     Cl45  ---------- ---------- ---------- ---------- ---------- --------GG TCACATGCAA AGAGTGTAGT AGCCATTTCA GCCTCTGAG- ---------- ---------- ----------
Consensus  .......... .......... .......... .......... .......... ........gg tcacatgcaa agagtgtagt agccatttca gcctctgag. .......... .......... ..........

           1691                                                                                                                                         1820
     gDNA  TAACTAATCT TATTTCAATC TTTTTCATTC AATGAAATGA ATGAGATAGA ATTTTATTTG ATTTACCAGT GCTAAGGAAG CGCATGAAGT AAAAATAATG TGTACATATC ACTTCTCTCA AAGCCCAGAT
     Cl47  ---------- ---------- ---------- ---------- ---------- ---------- ---------T GCTAAGGAAG CGCATGAAGT AAAAATAATG TGTACATATC ACTTCTCTCA AAGCCCAGAT
     Cl44  ---------- ---------- ---------- ---------- ---------- ---------- ---------T GCTAAGGAAG CGCATGAAGT AAAAATAATG TGTACATATC ACTTCTCTCA AAGCCCAGAT
     Cl45  ---------- ---------- ---------- ---------- ---------- ---------- ---------T GCTAAGGAAG CGCATGAAGT AAAAATAATG TGTACATATC ACTTCTCTCA AAGCCCAGAT
Consensus  .......... .......... .......... .......... .......... .......... .........t gctaaggaag cgcatgaagt aaaaataatg tgtacatatc acttctctca aagcccagat

           1821                                                                                                                                         1950
     gDNA  TAATTTTACT TTTAGAGGTT AGCATAGATT ATTCCCCCTT CTTAGTATAA AAATAAATAA ATTATCTCCT TGCTCTTCTT TAAATTATCT CCCAGCATAT GTAATACTTA ATCCTTGGCA CAGTATCTTT
     Cl47  TAATTTTACT TTTAGAG--- ---------- ---------- ---------- ---------- ---------- ---------- ---------- ---------- ---------- ---------- ----------
     Cl44  TAATTTTACT TTTAGAG--- ---------- ---------- ---------- ---------- ---------- ---------- ---------- ---------- ---------- ---------- ----------
     Cl45  TAATTTTACT TTTAGAG--- ---------- ---------- ---------- ---------- ---------- ---------- ---------- ---------- ---------- ---------- ----------
Consensus  taattttact tttagag... .......... .......... .......... .......... .......... .......... .......... .......... .......... .......... ..........

           1951                                                                                                                                         2080
     gDNA  TTTCTTTTCA CTCACTCCTT GTTTCTGCTG AAAGCTAATT GGTTGAAACT CATTTTTTAG TGAGTGATAC AGATGAGGAT GTCTTGCAAA TATTTTTTAA GGAGAGAGAA TTAAATGGGG ATTTTATATC
     Cl47  ---------- ---------- ---------- ---------- ---------- ---------- TGAGTGATAC AGATGAGGAT GTCTTGCAAA TATTTTTTAA GGAGAGAGAA TTAAATGGGG ATTTTATATC
     Cl44  ---------- ---------- ---------- ---------- ---------- ---------- TGAGTGATAC AGATGAGGAT GTCTTGCAAA TATTTTTTAA GGAGAGAGAA TTAAATGGGG ATTTTATATC
     Cl45  ---------- ---------- ---------- ---------- ---------- ---------- TGAGTGATAC AGATGAGGAT GTCTTGCAAT TATTTTTTAA GGAGAGAGAA TTAAATGGGG ATTTTATATC
     Cl53  ---------- ---------- ---------- ---------- ---------- ---------- TGAGTGATAC AGATGAGGAT GTCTTGCAAA TATTTTTTAA GGAGAGAGAA TTAAATGGGG ATTTTATATC
     Cl43  ---------- ---------- ---------- ---------- ---------- ---------- TGAGTGATAC AGATGAGGAT GTCTTGCAAA TATTTTTTAA GGAGAGAGAA TTAAATGGGG ATTTTATATC
Consensus  .......... .......... .......... .......... .......... .......... TGAGTGATAC AGATGAGGAT GTCTTGCAAa TATTTTTTAA GGAGAGAGAA TTAAATGGGG ATTTTATATC

           2081                                                                                                                                         2210
     gDNA  AAGAGCTTCC GATTTATTAT GGAGAAGAGA TTTCAGAAGT TCTGGTGATT ATGATATTAG CGAGCTCACC GACAACACTT CTCAACAAAT AGAGCAGGTG CAGCAGACTC ATTATATCTA ATTTCGAACT
     Cl47  AAGAGCTTCC GATTTATTAT GGAGAAGAGA TTTCAGAAGT TCTGGTGATT ATGATATTAG CGAGCTCACC GACAACACTT CTCAACAAAT AGAGCAG--- ---------- ---------- ----------
     Cl44  AAGAGCTTCC GATTTATTAT GGAGTAGAGA TTTCAGAAGT TCTGGTGATT ATGATATTAG CGAGCTCACC GACAACACTT CTCAACAAAT AGAGCAG--- ---------- ---------- ----------
     Cl45  AAGAGCTTCC GATTTATTAT GGAGAAGAGA TTTCAGAAGT TCTGGTGAT- ATGATATTAG CGAGCTCACC GACAACACTT CTCAACAAAT AGAGCAG--- ---------- ---------- ----------
     Cl53  AAGAGCTTCC GATTTATTAT GGAGAAGAGA TTTCAGAAGT TCTGGTGATT ATGATATTAG CGAGCTCACC GACAACACTT CTCAACAAAT AGAGCAG--- ---------- ---------- ----------
     Cl43  AAGAGCTTCC GATTTATTAT GGAGAAGAGA TTTCAGAAGT TCTGGTGATT ATGATATTAG CGAGCTCACC GACAACACTT CTCAACAAAT AGAGCAG--- ---------- ---------- ----------
Consensus  AAGAGCTTCC GATTTATTAT GGAGaAGAGA TTTCAGAAGT TCTGGTGATt ATGATATTAG CGAGCTCACC GACAACACTT CTCAACAAAT AGAGCAG... .......... .......... ..........
```

```
          2211                                                                                                                              2340
     gDNA ATTCCTATCC AATTTTAAAG ATTCTATCAA GTGTGTCAGT TTTGGCAATT TGTTGCCTTG TACTGATTCA TTGCAAATTT TGTATATTGA TTCGTTGGTC AAATAATTCA ATTTCACGTG TTTTTTATAA
Consensus .......... .......... .......... .......... .......... .......... .......... .......... .......... .......... .......... .......... ..........

          2341                                                                                                                              2470
     gDNA TATTTGTAGA TCATAGAGAC TGACAGTGAT GGTGGTTTGT TGAAACTTAC AAGAACCCAA GAGTGGCTAA CAGGTGACAA TTCTCCACCA ATAAACAAGA AGGTGACTGC TAAGGTATGC TTTAGAGTAG
     Cl47 ---------A TCATAGAGAC TGACAGTGAT GGTGGTTTGT TGAAACTTAC AAGAACCCAA GAGTGGCTAA CAGGTGACAA TTCTCCACCA ATAAACAAGA AGGTGACTGC TAAG------ ----------
     Cl44 ---------A TCATAGAGAC TGACAGTGAT GGTGGTTTGT TGAAACTTAC AAGAACCCAA GAGTGGCTAA CAGGTGACAA TTCTCCACCA ATAAACAAGA AGGTGACTGC TAAG------ ----------
     Cl45 ---------A TCATAGAGAC TGACAGTGAT GGTGGTTTGT TGAAACTTAC AAGAACCCAA GAGTGGCTAA CAGGTGACAA TTCTCCACCA ATAAACAAGA AGGTGACTGC TAAG------ ----------
     Cl53 ---------A TCATAGAGAC TGACAGTGAT GGTGGTTTGT TGAAACTTAC AAGAACCCAA GAGTGGCTAA CAGGTGACAA TTCTCCACCA ATAAACAAGA AGGTGACTGC TAAG------ ----------
     Cl43 ---------A TCATAGGGAC TGACAGTGAT GGTGGTTTGT TGAAACTTAC AAGAACCCAA GAGTGGCTAA CAGGTGACAA TTCTCCACCA ATAAACAAGA AGGTGACTGC TAAG------ ----------
Consensus .........A TCATAGaGAC TGACAGTGAT GGTGGTTTGT TGAAACTTAC AAGAACCCAA GAGTGGCTAA CAGGTGACAA TTCTCCACCA ATAAACAAGA AGGTGACTGC TAAG...... ..........

          2471                                                                                                                              2600
     gDNA TTTTCATTTT ACATCCCAAC TTTCATTTTC TTCTGTATGT TATTATCTTG TCAGGGAAAT TGGATATGAA ATTGATGTGT TACAATGCAT ATTTTATTTT GTCTAATGTT TTTCAAATTC ACCTTCTCTC
Consensus .......... .......... .......... .......... .......... .......... .......... .......... .......... .......... .......... .......... ..........

          2601                                                                                                                              2730
     gDNA TGTGGTAAAT AAATAATAAC CCAGTCATCC AATAATGTTG GAAACTTCAT TTCATATATC AACAGGCATT ACAGGACAGC AGTGCAAGAC GCATGAAACT GAACATGCTC AAATATGAAT CTGTACAATA
     Cl47 ---------- ---------- ---------- ---------- ---------- ---------- -----GCATT ACAGGACAGC AGTGCAAGAC GCATGAAACT GAACATGCTC AAATATGAAT CT--------
     Cl44 ---------- ---------- ---------- ---------- ---------- ---------- -----GCATT ACAGGACAGC AGTGCAAGAC GCATGAGACT GAACATGCTC AAATATGAAT CTGTACAATA
     Cl45 ---------- ---------- ---------- ---------- ---------- ---------- -----GCATT ACAGGACAGC AGTGCAAGAC GCATGAAACT GAACATGCTC AAATATGAAT CT--------
     Cl53 ---------- ---------- ---------- ---------- ---------- ---------- -----GCATT ACAGGATAGC AGTGCAAGAC GCATGAAACT GAACATGCTC AAATATGAAT CTGTACAATA
     Cl43 ---------- ---------- ---------- ---------- ---------- ---------- -----GCATT ACAGGACAGC AGTGCAAGAC GCATGAAACT GAACATGCTC AAATATGAAT CT--------
Consensus .......... .......... .......... .......... .......... .......... .....GCATT ACAGGAcAGC AGTGCAAGAC GCATGAaACT GAACATGCTC AAATATGAAT CT........

          2731                                                                                                                              2860
     gDNA CCTCTCTTGA CCTCGTTATT GTTCTTTCTT TGCTCAAGTT TAGTGACATC AGTGTTTTCT TACCTCAAGG CATGTAGGTT TTGATTAATT ACAGTTGTTC AGATGTTGTG TAAGGCTGTT GGCTGCTTAT
     Cl47 ---------- ---------- ---------- ---------- ---------- ---------- ---------- ---------- ---------- ---------- ---------- ---------- ----------
     Cl44 CCTCTCTTGA CCTCGTTATT GTTCTTTCTT TGCTCAAGTT TAGTGACATC AGTGTTTTCT TACCTCAAG- ---------- ---------- ---------- ---------- ---------- ----------
     Cl45 ---------- ---------- ---------- ---------- ---------- ---------- ---------- ---------- ---------- ---------- ---------- ---------- ----------
     Cl53 CCTCTCTTGA CCTCGTTATT GTTCTTTCTT TGCTCAAGTT TAGTGACATC AGTGTTTTCT TACCTCAAG- ---------- ---------- ---------- ---------- ---------- ----------
     Cl43 ---------- ---------- ---------- ---------- ---------- ---------- ---------- ---------- ---------- ---------- ---------- ---------- ----------
Consensus .......... .......... .......... .......... .......... .......... .......... .......... .......... .......... .......... .......... ..........

          2861                                                                                                                              2990
     gDNA TCTGTCATTT TTATTCTTAA TTGTAGCTCA AGAGGGAATT ACTGCTTCTA TCTGTGGGTA TTGGACTGGC TTGTAGTGGA TATTGCTTGG TTATTTTTTC CGTACAGGTA AACTTTACAT TTGCATGCTG
     Cl47 ---------- ---------- ------CTCA AGAGGGAATT ACTGCTTCTA TCTGTGGGTA TTGGACTGGC TTGTAGTGGA TATTGCTTGG TTATTTTTTC CGTACAG--- ---------- ----------
     Cl44 ---------- ---------- ------CTCA AGAGGGAATT ACTGCTTCTA TCTGTGGGTA TTGGACTGGC TTGTAGTGGA TATTGCTTGG TTATTTTTTC CGTACAG--- ---------- ----------
     Cl45 ---------- ---------- ------CTCA AGAGGGAATT ACTGCTTCTA TCTGTGGGTA TTGGACTGGC TTGTAGTGGA TATTGCTTGG TTATTTTTTC CGTACAG--- ---------- ----------
     Cl53 ---------- ---------- ------CTCA AGAGGGAATT ACTGCTTCTA TCTGTGGGTA TTGGACTGGC TTGTAGTGGA TATTGCTTGG TTATTTTTTC CGTACAG--- ---------- ----------
     Cl43 ---------- ---------- ------CTCA AGAGGGAATT ACTGCTTCTA TCTGTGGGTA TTGGACTGGC TTGTAGTGGA TATTGCTTGG TTATTTTTTC CGTACAG--- ---------- ----------
Consensus .......... .......... ......CTCA AGAGGGAATT ACTGCTTCTA TCTGTGGGTA TTGGACTGGC TTGTAGTGGA TATTGCTTGG TTATTTTTTC CGTACAG... .......... ..........

          2991                                                                                                                              3120
     gDNA TGTCTCAAAA AATAATAAAA AAGTCTTATC ATTTTGCTAT TATAATTCAG AACCAGATCA TTTTTGTGCT TCTTACCACT CAATTATGTT CTCTTTCTTC ATGATAAGTC TAACTTTGGA TGGATGCCAA
Consensus .......... .......... .......... .......... .......... .......... .......... .......... .......... .......... .......... .......... ..........

          3121                                                                                                                              3250
     gDNA TGTCTTACCA TAATGCTTTA ACTTCAAACA CTACCCCATG GTGTAAGTTT CAAATTATTT CCTTCACTGA TGTGTGCCAT TCTCCTAAAT GTGTAGGCTG CTATAAGTTA TGCGATTGGA GTCCTTTTCA
     Cl47 ---------- ---------- ---------- ---------- ---------- ---------- ---------- ---------- ---------- ------GCTG CTATAAGTTA TGCGATTGGA GTCCTTTTCA
     Cl44 ---------- ---------- ---------- ---------- ---------- ---------- ---------- ---------- ---------- ------GCTG CTATAAGTTA TGCGATTGGA GTCCTTTTCA
     Cl45 ---------- ---------- ---------- ---------- ---------- ---------- ---------- ---------- ---------- ------GCTG CTATAAGTTA TGCGATTGGA GTCCTTTTCA
     Cl53 ---------- ---------- ---------- ---------- ---------- ---------- ---------- ---------- ---------- ------GCTG CTATAAGTTA TGCGATTGGA GTCCTTTTCA
     Cl43 ---------- ---------- ---------- ---------- ---------- ---------- ---------- ---------- ---------- ------GCTG CTATAAGTTA TGCGATTGGA GTCCTTTTCA
Consensus .......... .......... .......... .......... .......... .......... .......... .......... .......... ......GCTG CTATAAGTTA TGCGATTGGA GTCCTTTTCA

          3251                                                                                                                              3380
     gDNA GGTTTGTTAT TGCATTGTAT TATATTGATA CTGTCATATT GAATTATTTG TTAAATGAGC TTAAGGGCCA GTAGTAAGAC AGTTAGAGCC TAAATTGATA TTGTTCATCA TTGTTTTTTT TGTGTGTGAG
     Cl47 G--------- ---------- ---------- ---------- ---------- ---------- ---------- ---------- ---------- ---------- ---------- ---------- ----------
     Cl44 G--------- ---------- ---------- ---------- ---------- ---------- ---------- ---------- ---------- ---------- ---------- ---------- ----------
     Cl45 G--------- ---------- ---------- ---------- ---------- ---------- ---------- ---------- ---------- ---------- ---------- ---------- ----------
     Cl53 G--------- ---------- ---------- ---------- ---------- ---------- ---------- ---------- ---------- ---------- ---------- ---------- ----------
     Cl43 G--------- ---------- ---------- ---------- ---------- ---------- ---------- ---------- ---------- ---------- ---------- ---------- ----------
Consensus G......... .......... .......... .......... .......... .......... .......... .......... .......... .......... .......... .......... ..........
```

```
          3381                                                                                                                                3510
     gDNA AGAAAAGAAA AGATTAAACT GTTCTTAAAA TTTGAGATTC AGACAATAGG ATAAAAAATG TAACTATCCA CACATTCTAA TTTAATCTTT TTTTTTTAAT CTTAAGAATC GAAGATAGAT ATCAAAGAGT
Consensus .......... .......... .......... .......... .......... .......... .......... .......... .......... .......... .......... .......... ..........

          3511                                                                                                                                3640
     gDNA TTATAACAAC TCACGCATTC TAATTTAATT TAACAATAAT AGGTGATGAA TTAATTGAAT AGGTTGAAAC CTGGCAAGAC ATATTTGCCG GTGGCATCAA TGACAGTGAC ATCATCTCCA ACCTGGGAAT
     Cl47 ---------- ---------- ---------- ---------- ---------- ---------- --GTTGAAAC CTGGCAAGAC ATATTTGCCG GTGGCATCAA TGACAGTGAC ATCATCTCCA ACCTGGGAAT
Consensus .......... .......... .......... .......... .......... .......... .......... .......... .......... .......... .......... .......... ..........

          3641                                                                                                                                3770
     gDNA GGAAATGAAT CATAAGCAGA AAGTAACAAA AAGCGCAAGG TGTTAGAAAG CAAATTAAGC ACTGCTACTG CTAGGGGGTT TCAATTCATC AAATACCCTG AAGTTAGGAT TAACTGCAAC AATGATGCCA
     Cl47 GGAAATGAAT CATAAGCAGA AAGTAACAAA AAGCGCAAGG TGTTAGAAAG CAAATTAAGC ACTGCTACTG CTAGGGGGTT TCAATTCATC AAATACCCTG AAGTTAGGAT TAACTGCAAC AATGATGCCA
Consensus .......... .......... .......... .......... .......... .......... .......... .......... .......... .......... .......... .......... ..........

          3771                                                                                                                                3900
     gDNA TCTTCCACGT AAACATCAGC CATCTGTTGG TGGTGACCAT TCACAACCGT GCCTCCCTTG ATCAACAACT TGGATGATGG AATTTCAGTC CCAGCATCAC AAAACTGACT CACTCGAAAA GGACAAACAT
     Cl47 TCTTCCACGT AAACATCAGC CATCTGTTGG TGGTGACCAT TCACAACCGT GCCTCCCTTG ATCAACAACT TGGATGATGG AATTTCAGTC CCAGCATCAC AAAGCTGACT CACTCGAAAA GGACAAACAT
Consensus .......... .......... .......... .......... .......... .......... .......... .......... .......... .......... .......... .......... ..........

          3901                                                                                                                                4030
     gDNA GAATTAAACA TAGAACTAGT GGAAATGAAA ATCTCAGCTC AGTACTAATG ATCATAGCTA CTTAAAACAG TTAAGAATCG AAGCTCTAAC TCTTGGAAAA CGCAGAACGG GGTAAGTAAA CTAGGTGAGC
     Cl47 GAATTAAACA TAGAACTAGT GGAAATGAAA ATCTCAGCTC AGTACTAATG ATCATAGCTA CTTAAAACAG TTAAGAATCG AAGCTCTAAC TCTTGGAAAA CGCAGAACGG GGTAAGTAAA TTAGGTGAGC
Consensus .......... .......... .......... .......... .......... .......... .......... .......... .......... .......... .......... .......... ..........

          4031                                                                                                                                4160
     gDNA ATAGGTCAAC ACATAAGAGA ATTATTCCCT GCATTCAGTT TTTATAAAAC TTGCAGAAGT ATTAATGCAA CTCCCCCTCC CTTTGGTAGT CACTAAAAGA ACAAGCGCAC TTGAACTTCT ATGGTTGATG
     Cl47 ATAGGTCAAC ACATAAGAGA ATTATTCCCT GCATTCAGTT TTTATAAAAC TTGCAGAAGT ATTAATGCAA CTCCCCCTCC CTTTGGTAGT CACTAAAAGA ACAAGCGCAC TTGAACTTCT ATGGTTGATG
Consensus .......... .......... .......... .......... .......... .......... .......... .......... .......... .......... .......... .......... ..........

          4161                                                                                                                                4290
     gDNA TAAGGGTACG TGTCTTTGAT TTTCCCCCAT GCTTGTTTGC AAGTGTAAAC ATGCTCAATT TCACTCTTTT CAATTCTTCC TACAATTGCT TATTCATTTG CTTACTGTTT CAAAATTTAA AATAAATACT
     Cl47 TAAGGGTACG TGTCTTTGAT TTTCCCCCAT GCTTGTTTGC AAGTGTAAAC ATGCTCAATT TCACTCTTTT CAATTCTTCC TACAATTGCT TATTCATTTG CTTACTGTTT CAAAATTTAA AATAAATACT
Consensus .......... .......... .......... .......... .......... .......... .......... .......... .......... .......... .......... .......... ..........

          4291                                                                                                                                4420
     gDNA GTACAGTCAT AAATAATAGT ATATATGGCC TAGTAGTATA TTTTGATTTT GTAGTATCTC TAATTTACAT GGAAACCGCC TTTTTTAAAA ATTGTCTTTG CAAAAATTCC CTGCTAGAAC ATTTATCCAG
     Cl47 GTACAGTCAT AAATAATAGT ATATATGGCC TAGTAGTATA TTTTGATTTT GTAGTATCTC TAATTTACAT GGAAACCGCC TTTTTTAAAA ATTGTCTTTG CAAAAATTCC CTGCTAGAAC ATTTATCCAG
Consensus .......... .......... .......... .......... .......... .......... .......... .......... .......... .......... .......... .......... ..........

          4421                                                                                                                                4550
     gDNA TGGTATTCTT GAAAAAAAAT ATATGTAGAT TTATTGTTTA CATTATGTAT TCTGTCTGAA GCTTGTAAAG AGCTTTATGT TCGTTTATGC CTTTCCCATT TTTTTTCTCT GGAAGGGGTG AAATATAGGA
     Cl47 TGGTATTCTT GAAAAAAAAT ATATGTAGAT TTATTGTTTA CATTATGTAT TCTGTCTGAA GCTTGTAAAG AGCTTTATGT TCGTTTATGC CTTTCCCATT TTTTTTCTCT GGAAGGGGTG AAATATAGGA
Consensus .......... .......... .......... .......... .......... .......... .......... .......... .......... .......... .......... .......... ..........

          4551                                                                                                                                4680
     gDNA TATTCTACTT TTTCAGTTTT TAGGTTCTAA TAAGGCATAA TTAAGTGGTA GACTCAGCTT TAAAATTTTG TTCTTTCCTT TATATGGAGA ATTATGTTTC AGATTAATGT GACTTGTAAT AAAACCTTTT
     Cl47 TATTCTACTT TTTCAGTTTT TAGGTTCTAA TAAGGCATAA TTAAGTGGTA GACTCGGCCT TAAAATTTTG TTCTTTCCTT TATATGGAGA ATCATGTTTC AGATTAATGT GACTTGTAAT AAAACCTTTT
Consensus .......... .......... .......... .......... .......... .......... .......... .......... .......... .......... .......... .......... ..........

          4681                                                                                                                                4810
     gDNA GAGCAAAACT GACTTGTTTT TATAAATTTC AGATGGATGT GCGGACTAAT TCTCTCGTCA GGCCATCAGG GACAATTGAC AAAGATAAGG AAAAGCTACG GATTGCCAAC AATGGTGTCG TTCAGAGTGA
     Cl47 GAGCAAAACT GACTTGTTTT TATAAATTTC AGATGGATGT GCGGACTAAT TCTCTCGTCA GGCCATCAGG GACAATTGAC AAAGATAAGG AAAAGCTACG GATTGCCAAC AATGGTGTCG TTCAGAGTGA
     Cl44 ---------- ---------- ---------- --ATGGATGT GCGGACTAAT TCTCTCGTCA GGCCATCAGG GACAATTGAC AAAGATAAGG AAAAGCTACG GATTGCCAAC AATGGTGTCG TTCAGAGTGA
     Cl45 ---------- ---------- ---------- --ATGGATGT GCGGACTAAT TCTCTCGTCA GGCCATCAGG GACAATTGAC AAAGATAAGG AAAAGCTACG GATTGCCAAC AATGGTGTCG TTCAGAGTGA
     Cl53 ---------- ---------- ---------- --ATGGATGT GCGGACTAAT TCTCTCGTCA GGCCATCAGG GACAATTGAC AAAGATAAGG AAAAGCTACG GATTGCCAAC AATGGTGTCG TTCAGAGTGA
     Cl43 ---------- ---------- ---------- --ATGGATGT GCGGACTAAT TCTCTCGTCA GGCCATCAGG GACAATTGAC AAAGATAAGG AAAAGCTACG GATTGCCAAC AATGGTGTCG TTCAGAGTGA
Consensus .......... .......... .......... ..ATGGATGT GCGGACTAAT TCTCTCGTCA GGCCATCAGG GACAATTGAC AAAGATAAGG AAAAGCTACG GATTGCCAAC AATGGTGTCG TTCAGAGTGA

          4811                                                                                                                                4940
     gDNA AGAACAAACC TTACCTATTG GAGGTGATGG TTGGGAAAAG TCAAAAATGA AGAAGAAGCG TTCCTGTATC AAACTAGATG TTTCTCCCAG TACAACATTG ACTAAACCTG TTAACACCTT CCAAGAAACT
     Cl47 AGAACAAACC TTACCTATTG GAGGTGATGG TTGGGAAAAG TCAAAAATGA AGAAGAAGCG TTCCTGTATC AAACTAGATG TTTCTCCCAG TACAACATTG ACTAAACCTG TTAACACCTT CCAAGAAACT
     Cl44 AGAACAAACC TTACCTATTG GAGGTGATGG TTGGGAAAAG TCAAAAATGA AGAAGAAGCG TTCCTGTATC AAACTAGATG TTTCTCCCAG TACAACATTG ACTAAACCTG TTAACACCTT CCAAGAAACT
     Cl45 AGAACAAACC TTACCTATTG GAGGTGATGG TTGGGAAAAG TCAAAAATGA AGAAGAAGCG TTCCTGTATC AAACTAGATG TTTCTCCTAG TACAACATTG ACTAAACCTG TTAACACCTT CCAAGAAACT
     Cl53 AGAACAAACC TTACCTATTG GAGGTGATGG TTGGGAAAAG TCAAAAATGA AGAAGAAGCG TTCCTGTATC AAACTAGATG TTTCTCCCAG TACAACATTG ACTAAACCTG TTAACACCTT CCAAGAAACT
     Cl43 AGAACAAACC TTACCTATTG GAGGTGATGG TTGGGAAAAG TCAAAAATGA AGAAGAAGCG TTCCTGTATC AAACTAGATG TTTCTCCCAG TACAACATTG ACTAAACCTG TTAACACCTT CCAAGAAACT
Consensus AGAACAAACC TTACCTATTG GAGGTGATGG TTGGGAAAAG TCAAAAATGA AGAAGAAGCG TTCCTGTATC AAACTAGATG TTTCTCCcAG TACAACATTG ACTAAACCTG TTAACACCTT CCAAGAAACT
```

```
          4941                                                                                                                                                5070
     gDNA AAACAGGGAA TGCAACAAAG ACTTGCTACC GATTCGCGAT TCTTTCAGGT ATGAATGTGT TATTTAATTA TTTTTTTTTT GTTCTCATAT AATATTTCTT ATTTTAGGCT AAAATAATAT TTTATGGGAA
     Cl47 AAACAGGGAA TGCAACAAAG ACTTGCTACC GATTCGCGAT TCTTTCAG-- ---------- ---------- ---------- ---------- ---------- ---------- ---------- ----------
     Cl44 AAACAGGGAA TGCAACAAAG ACTTGCTACC GATTCGCGAT TCTTTCAG-- ---------- ---------- ---------- ---------- ---------- ---------- ---------- ----------
     Cl45 AAACAGGGAA TGCAACAAAG ACTTGCTACC GATTCGCGAT TCTTTCAG-- ---------- ---------- ---------- ---------- ---------- ---------- ---------- ----------
     Cl53 AAACAGGGAA TGCAACAAAG ACTTGCTACC GATTCGCGAT TCTTTCAG-- ---------- ---------- ---------- ---------- ---------- ---------- ---------- ----------
     Cl43 AAACAGGGAA TGCAACAAAG ACTTGCTACC GATTCGCGAT TCTTTCAG-- ---------- ---------- ---------- ---------- ---------- ---------- ---------- ----------
Consensus AAACAGGGAA TGCAACAAAG ACTTGCTACC GATTCGCGAT TCTTTCAG.. .......... .......... .......... .......... .......... .......... .......... ..........

          5071                                                                                                                                                5200
     gDNA GATTAGAAAG TAGGCACATT TTTTGTGGGA GGATTTAGGA TAAGAAAATC CTCCCTTAAA ATGAGGAAAA AAGAGTCAAA ATGTCCAAAA AAAAATGTTA GGCTTTTCAA TCTTTATGCA CATCAACAAA
Consensus .......... .......... .......... .......... .......... .......... .......... .......... .......... .......... .......... .......... ..........

          5201                                                                                                                                                5330
     gDNA AGAAATTCCT TTGAAATAAC CACGAGCATA TCCCTAGAGA AAATCCATAC ATGAATTTTG GGGCAAAGAG ACATTCTTGA AAATGCAATC ATTCCAATTC CTCTCTAATA AACTGCCTGA TAACTGTATA
Consensus .......... .......... .......... .......... .......... .......... .......... .......... .......... .......... .......... .......... ..........

          5331                                                                                                                                                5460
     gDNA AATTGCTTAG TTCCATAGAA TTGTAGCCTC CTGTTTGCTG CCAAATGTTA CAATTGGAGA ATGAAAATAA ATTATAGGCA GTAACTGTTT GGAGTTCTCT CTAGAGTGTT AATTGTGAAA GGATTTTCTT
Consensus .......... .......... .......... .......... .......... .......... .......... .......... .......... .......... .......... .......... ..........

          5461                                                                                                                                                5590
     gDNA CAAAGAAAAA CAATACAGTT GTGAAGGATT TTGCTGATAT TAAGGGTCAT GAATTTAAAA TCTTTGATGG ATGATATCAT GTAACAATTT ATTTATTTAT TTGCTCTATC CAATTCTGAT TTATTTTCTT
Consensus .......... .......... .......... .......... .......... .......... .......... .......... .......... .......... .......... .......... ..........

          5591                                                                                                                                                5720
     gDNA GCTTAAACCT AGCCGATAAA TATAATAATT TCATGTATTT TTTTTAGGTC AGTAGTTTGT AATGGAACTA TTGGAGTTGG AAAATCAGAT GGTATCTCTC AACAAACTGG GTTGGGCATA CGAGCTTCTA
     Cl47 ---------- ---------- ---------- ---------- -------GTC AGTAGTTTGT AATGGAACTA TTGGAGTTGG AAAATCAGAT GGTATCTCTC AACAAACTGG GTTGGGCATA CGAGCTTCTA
     Cl44 ---------- ---------- ---------- ---------- -------GTC AGTAGTTTGT AATGGAACTA TTGGAGTTGG AAAATCAGAT GGTATCTCTC AACAAACTGG GTTGGGCATA CGAGCTTCTA
     Cl45 ---------- ---------- ---------- ---------- -------GTC AGTAGTTTGT AATGGAACTA TTGGAGTTGG AAAATCAGAT GGTATCTCCC AACAAACTGG GTTGGGCATA CGAGCTTCTA
     Cl53 ---------- ---------- ---------- ---------- -------GTC AGTAGTTTGT AATGGAACTA TTGGAGTTGG AAAATCAGAT GGTATCTCTC AACAAACTGG GTTGGGCATA CGAGCTTCTA
     Cl43 ---------- ---------- ---------- ---------- -------GTC AGTAGTTCGT AATGGAACTA TTGGAGTTGG AAAATCAGAT GGTATCTCTC AACAAACTGG GTTGGGCATA CGAGCTTCTA
Consensus .......... .......... .......... .......... .......GTC AGTAGTTtGT AATGGAACTA TTGGAGTTGG AAAATCAGAT GGTATCTCtC AACAAACTGG GTTGGGCATA CGAGCTTCTA

          5721                                                                                                                                                5850
     gDNA CCCCTAGAAA CAACCAAGAT AATAATTCCC TTGTCAATGA TAGGAGGGGT CGTCCTGTTA GTTCAGACAA GGAAAGGGTG AACTTCAGAG TTGTAAACAA GTAAATACAA GCTGTGGAAC CCCTTTTCTT
     Cl47 CCCCTAGAAA CAACCAAGAT AATAATTCCC TTGTCAATGA TAGGAGGGGT CGTCCTGTTA GTTCAGACAA GGAAAGGGTG AACTTCAGAG TTGTAAACAA ---------- ---------- ----------
     Cl44 CCCCTAGAAA CAACCAAGAT AATAATTCCC TTGTCAATGA TAGGAGGGGT CGTCCTGTTA GTTCAGACAA GGAAAGGGTG AACTTCAGAG TTGTAAACAA ---------- ---------- ----------
     Cl45 CCCCTAGAAA CAACCAAGAT AATAATTCCC TTGTCAATGA TAGGAGGGGT CGTCCTGTTA GTTCAGACAA GGAAAGGGTG AACTTCAGAG TTGTAAACAA ---------- ---------- ----------
     Cl53 CCCCTAGAAA CAACCAAGAT AATAATTCCC TTGTCAATGA TAGGAGGGGT CGTCCTGT-A GTTCAGACAA GGAAAGGGTG AACTTCAGAG TTGTAAACAA ---------- ---------- ----------
     Cl43 CCCCTAGAAA CAACCAAGAT AATAATTCCC TTGTCAATGA TAGGAGGGGT CGTCCTGTTA GTTCAGACAA GGAAAGGGTG AACTTCAGAG TTGTAAACAA ---------- ---------- ----------
Consensus CCCCTAGAAA CAACCAAGAT AATAATTCCC TTGTCAATGA TAGGAGGGGT CGTCCTGTtA GTTCAGACAA GGAAAGGGTG AACTTCAGAG TTGTAAACAA .......... .......... ..........

          5851                                                                                                                                                5980
     gDNA TATATGATTG TGCGTTTGGG AAAAAGAATT GTCCTTCACT AAAATGTTTT TGCATGTTGC TTTCTCATTT TCTCCTTATT TTCGTCTTGT TTTGGTAGAA TTGTTCAATT GTTTGCTTTC TGTTTGCTAT
Consensus .......... .......... .......... .......... .......... .......... .......... .......... .......... .......... .......... .......... ..........

          5981                                                                                                                                                6110
     gDNA GGAGCCTTGC GATTATAGTT CATATATCTC TTCATCTTTG TGATATTTTT TCTGGTATTG ATGATTCATT CAGTATCTGT GTTATGTTCT ATGACCAGGG CAACTGCACG TGATGAATTT AATTCATCTA
Consensus .......... .......... .......... .......... .......... .......... .......... .......... .......... .......... .......... .......... ..........

          6111                                                                                                                                                6240
     gDNA GCCCTACCTC AAGTGCTAAA ATAAACACTG CTATCTGTGT TATGATATTT TTTATTAGTT CTATTGCATG CAATCAAGTT AATGGTATAC GTCACTATTA TTACTGAGAA GTGGTTAAAT GTCAAGTAGA
Consensus .......... .......... .......... .......... .......... .......... .......... .......... .......... .......... .......... .......... ..........

          6241                                                                                                                                                6370
     gDNA TCAAGCATGC AATATATAAT TTTGGTTTAG ATATTTATTT GGTGTTATAG ATATGATCTA GAAGATAAAA TACAGCACAG CCATTAACTG TTGTAGCTTA ATATGACATT GACACTGATC GATTTCTGTA
Consensus .......... .......... .......... .......... .......... .......... .......... .......... .......... .......... .......... .......... ..........

          6371                                                                                                                                                6500
     gDNA TGTATCCAAT GTCATTTGAA AACTGTATTT GCTAGCTTCC ATTACCTCTT GTTTAGGTTA AACTAAACCC AGAATCTAAA TTGAAAATGC TCACTGTAAT TGGCTTGTAT CTGGGAATAT GGGTGGGGTG
Consensus .......... .......... .......... .......... .......... .......... .......... .......... .......... .......... .......... .......... ..........

          6501                                                                                                                                                6630
     gDNA GGGTGTGGAA TAAACTTCTC CATAAGCACT TCTTGGGAAA AAATGAGAAG GTAAAATGCA TTGAGCTTCT CCATAAGCTA AAAAATCAGC TTATACACCT TATCTTTTGG AGAAGATAAA CGAAAAGAGC
Consensus .......... .......... .......... .......... .......... .......... .......... .......... .......... .......... .......... .......... ..........
```

```
              6631                                                                                                                                       6760
         gDNA TTTTATAAAA ATTAAGTGCA TAAGTTGATT TTAGCTTATG TGCAAAATTC AATTCATTAT ATCTTCTTAT TTTGGTAGTG AAGTTTATCA AAACGAGGTC AATGAATACA ACTTTTTTGG CTTTAGTAAT
    Consensus .......... .......... .......... .......... .......... .......... .......... .......... .......... .......... .......... .......... ..........

              6761                                                                                                                                       6890
         gDNA TTCGAGCAAC AAATAGCATT GATGTGACCT TGATTTGACT ATTTCAGTGT TAGAACTTTT TTGAAAACTA CAAATGTTTA TTATGTTCTG CAAATTCTAT ATTATCTTTC TATTAGAAAA GTCTTTGTTA
    Consensus .......... .......... .......... .......... .......... .......... .......... .......... .......... .......... .......... .......... ..........

              6891                                                                                                                                       7020
         gDNA TTTAGAGATC TTGCATGCTA ATCTTTATTA TTAAATATTA ATTTCTATTT ACTAAGTTTT GAGCCTAGAT ATATGTTTAT TTAGAGATCT GAAGCTATAT ACTATTGCCT GCATTTACTT ATGTTTATTA
    Consensus .......... .......... .......... .......... .......... .......... .......... .......... .......... .......... .......... .......... ..........

              7021                                                                                                                                       7150
         gDNA TTGGCATAAC CTGTTGGTCT AAATTTTAGT TTCTAGTGAT GCATTACTGT TTTATGCAGG TCTTTTCTGA CTTGGAGCGT TCAGTGTGTA GAATGGTCCA TCGACAGGTT TGATTCTGAT AAATTGCTTT
         Cl47 ---------- ---------- ---------- ---------- ---------- ---------G TCTTTTCAGA CTTGGAGCGT TCAGTGTGTA GAATGGTCCA TCGACAG--- ---------- ----------
         Cl44 ---------- ---------- ---------- ---------- ---------- ---------G TCTTTTCTGA CTTGGAGCGT TCAGTGTGTA GAATGGTCCA TCGACAG--- ---------- ----------
         Cl45 ---------- ---------- ---------- ---------- ---------- ---------G TCTTTTCTGA CTTGGAGCGT TCAGTGTGTA GAATGGTCCA TCGACAG--- ---------- ----------
         Cl53 ---------- ---------- ---------- ---------- ---------- ---------G TCTTTTCTGA CTTGGAGCGT TCAGTGTGTA GAATAGTCCA TCGACAG--- ---------- ----------
         Cl43 ---------- ---------- ---------- ---------- ---------- ---------G TCTTTTCTGA CTTGGAGCGT TCAGTGTGTA GAATGGTCCA TCGACAG--- ---------- ----------
    Consensus .......... .......... .......... .......... .......... .........G TCTTTTCtGA CTTGGAGCGT TCAGTGTGTA GAATgGTCCA TCGACAG--- .......... ..........

              7151                                                                                                                                       7280
         gDNA TAATGTTTTC TACTGTTGGT TTGAGAGATT ATATTTACAT TTTATTTTAG TAACTTGAGT ATCGTATTCC GATTATGGCA GAACTTGATC AAGTTCAAGT GTATGATGAA TTCCTTTTTG TTTAGCATTG
    Consensus .......... .......... .......... .......... .......... .......... .......... .......... .......... .......... .......... .......... ..........

              7281                                                                                                                                       7410
         gDNA GGATCATGGA ACAAGTTTCC TTGATTTCAT TTAAAATATT TTGAAGATTT AAATCAGTTC ATTTGATTCT GTAATATTAA GATTTGTAAC TTAATTACAA TTTTGATTGT ACATACCTTT CTCTGTTCCC
    Consensus .......... .......... .......... .......... .......... .......... .......... .......... .......... .......... .......... .......... ..........

              7411                                                                                                                                       7540
         gDNA ATGGAGCTTA TTTTTTAATA AATAGACCTT TTGGATTTGT ATTTAGTTTT TGTCTTACTA CGGATGCTTC TTTTCTTAAC TTGGGGGATT CGTTTTTTAA TTTTTTTGTA GGTTGCCACA ATTGCTTGGT
         Cl47 ---------- ---------- ---------- ---------- ---------- ---------- ---------- ---------- ---------- ---------- ---------- -GTTGCCACA ATTGCTTGGT
         Cl44 ---------- ---------- ---------- ---------- ---------- ---------- ---------- ---------- ---------- ---------- ---------- -GTTGCCACA ATTGCTTGGT
         Cl45 ---------- ---------- ---------- ---------- ---------- ---------- ---------- ---------- ---------- ---------- ---------- -GTTGCCACA ATTGCTTGGT
         Cl53 ---------- ---------- ---------- ---------- ---------- ---------- ---------- ---------- ---------- ---------- ---------- -GTTGCCACA ATTGCTTGGT
         Cl43 ---------- ---------- ---------- ---------- ---------- ---------- ---------- ---------- ---------- ---------- ---------- -GTTGCCACA ATTGCTTGGT
    Consensus .......... .......... .......... .......... .......... .......... .......... .......... .......... .......... .......... .GTTGCCACA ATTGCTTGGT

              7541                                                                                                                                       7670
         gDNA TGGAAGCTGA TTCTGTTTGT GGCAGCCACT CTATATGA-C TTCACATTTT CTTATTACCT TTTAATCTAT AATATCATGT ATTTATGCAA ATGAACTGCA TATACCTGTG TAATGAACTG CTTGCAAATG
         Cl47 TGGAAGCTGA TTCTGTTTGT GGCAGCCACT CTATATGAAC TTCACA
         Cl44 TGGAAGCTGA TTCTGTTTGT GGCAGCCACT CTATATGA-C TTCACA
         Cl45 TGGAAGCTGA TTCTGTTTGT GGCAGCCACT CTATATGA-C TTCACA
         Cl53 TGGAAGCTGA TTCTGTTTGT GGCAGCCACT CTATATGA-C TTCACA
         Cl43 TGGAAGCTGA TTCTGTTTGT GGCAGCCACT CTATATGA-C TTCACA
    Consensus TGGAAGCTGA TTCTGTTTGT GGCAGCCACT CTATATGA.C TTCACA.... .......... .......... .......... .......... .......... .......... .......... ..........

              7671                                                                                                                                       7800
         gDNA AAGTGTGAGA GGTGAATGCT TTTATGAAAA TAGATTGATT GATGAATTTC TGTTACCATT CAGTGAAATC TGTCAAAGTA TCATGTAGTT ATGCTCACAA TTTTTTTAAA TGTATTTTGA TTTTTAAACA
    Consensus .......... .......... .......... .......... .......... .......... .......... .......... .......... .......... .......... .......... ..........

              7801                                                                                                                                       7930
         gDNA ATTTATGTAA GTAATCTTTC CTTGCTGTTG TTGAGAAGAA TAAAAAATTG TTTGTTCTAG CATTTTAGAA CTAAGTGATC TTTATTTTAT TTTTATCTGA TAAACACTCG GTGATCCTTA TATCAACTTT
   Consensus .......... .......... .......... .......... .......... .......... .......... .......... .......... .......... .......... .......... ..........

              7931                                                                                                                                       8060
         gDNA TTATCAATGT CCTTTGTTTG TTCAGGGATT TATTGCATCC GCCCTTAATT ATGGACTCAT TACATGGTGA CGAAGGTTTC AGCATTGCTT TATGAAAGTT TTGTTGTCAA AGTTTGTAAT TTGTGGACAA
    Consensus .......... .......... .......... .......... .......... .......... .......... .......... .......... .......... .......... .......... ..........

              8061                                                                                                                                       8190
         gDNA GTTACAACTG AAGTTAGCAA TGTTAGATAT TCCTATGTAA TTTTAAATTC TTGCAAAACT ATATACTATC ATTTAATTAT GTAAATTCTT TTATTATGTG ATTAAACAAT TTTATAATTC TTTAAAATGA
   Consensus .......... .......... .......... .......... .......... .......... .......... .......... .......... .......... .......... .......... ..........

              8191                                                                                                                                       8320
         gDNA TGTAAATTCT TTCATTAATT ATGTAAATTC TTTTATTATG TAAATTCTTT TGCTTCAAAT CAATGTATAG AATAATTGAA ACACTACCTA AAAAACTAGA CCTGCACCCA AAAAATTAAA AAAAAATATA
    Consensus .......... .......... .......... .......... .......... .......... .......... .......... .......... .......... .......... .......... ..........
```

```
          8321                                                                                                                                      8450
     gDNA TATATTCTAA GACGGTTGTC GGAAACCGTC TTAGTATGTT TCACTTTCTA AGACGTTTGT ATGAATAACC GCCTTAGAAA CTTTTACAAA CAAAAACATA CTAAGACTGT TTCACCTGAA CCGTCTTAGT
Consensus .......... .......... .......... .......... .......... .......... .......... .......... .......... .......... .......... .......... ..........

          8451                                                                                                                                      8580
     gDNA ATGTTTGACA TTCTAAGACG GTTTTCGTAA CAATCGTCTT AGAAAGTCTC ACAAACAAAT GACATACTAA GACGGTTTTC GTAACAATCG CCTTAGAAAG TCTCACAAAC AAATGACATA CTAAGACGGT
Consensus .......... .......... .......... .......... .......... .......... .......... .......... .......... .......... .......... .......... ..........

          8581                                                                                                                                      8710
     gDNA TGTTGAAAAA TCGTCTTAGT ATGTCTCACT TTCTATGGCG GTTTTTACGA AAACCGTCTT AAAAACCTTC AAAAAAAGGA CATACTAAGA TGGTTGCAGG AGAAAACCGT CTTAGTATGT CTAACTTTCT
Consensus .......... .......... .......... .......... .......... .......... .......... .......... .......... .......... .......... .......... ..........

          8711                                                                                                                                      8840
     gDNA AAGACGGTTT TTGCAATAAC CGTCTTAGTA AGTCTCACTT TCTAAGACGG TTGTAGAAAA ACCGTCCTAG TAAGTCTCAC TTTTTAAGAC GGTTTTTGTA ATAACCGTCG TAGAAAGRTT GGTTTATTAT
Consensus .......... .......... .......... .......... .......... .......... .......... .......... .......... .......... .......... .......... ..........

          8841                                                                                                                                      8970
     gDNA AACGGTTAGT AGATGATAAC CGCCTTGAAT TAATAATAAA TTTTAAGACG GTTATTCTTA AAACCGTCTT AACAACTCCA ATCTTTAAAG ACGGTTTGAA AACCGTCGTT GTAGAGGTGG GCACATTTTA
Consensus .......... .......... .......... .......... .......... .......... .......... .......... .......... .......... .......... .......... ..........

          8971                                                                               9051
     gDNA CGACGCTGCG TGCTATGACG GTTCAAAACC GTCGTAAAAT GACTTGCAGA ACCGACTTAA AAAGCTTTAT TTGTAGTAGT G
Consensus .......... .......... .......... .......... .......... .......... .......... .......... .
```
