# Supplementary material for: A putative autonomous 20.5 kb-CACTA transposon insertion in an F3'H allele identifies a new CACTA transposon subfamily in Glycine max
Source: BMC Plant Biol. 2008 Dec 2;8:124. doi: 10.1186/1471-2229-8-124 (PMC2613891; doi:10.1186/1471-2229-8-124)
Supplement: Additional file 6 — Alignment of Mosaic-cDNA sequences to Tgmt* genomic sequence. The sequences from RT-PCR derived cDNA clones amplified with primers 1 and 7 (Figure 8) (No.:37, 38, 39, 42 and 56) were aligned to Tgmt* genomic sequence with MultAlin program (Corpet, 1988). It revealed the splicing site (marked with an asterisk) that does not conform to the canonical GT-AG intron-exon splice boundaries (GA, following the 346 bp of Exon-1 and AT, prior to the 23 bp of Intron-10). The 23 bp of Intron-10 have been highlighted in yellow. The exon sequences appear in red and blue depending on the number of clones bearing the exon. [file 1471-2229-8-124-S6.pdf]

Additional file 6: Alignment of Mosaic-cDNA sequences to *Tgmt** genomic sequence

```
           1                                                                                                                              130
     gDNA  AAAACAGCAC GCATAACTGA AGAGTACGAA AATGGGGTTG AAGGTTTCTT AAAATTTGCT AAAGATAATG CATCCGACAA TGGTGGACTA TACTTTTGTC CTTGTGTTAA ATGTTTGAAT GGGCGACGAC
     Cl37    AACAGCAC GCATAACTGA AGAGTACGAA AATGGGGTTG AAGGTTTCTT AAAATTTGCT AAAGATAATG CATCCGACAA TGGTGGACTA TACTTTTGTC CTTGTGTTAA ATGTTTGAAT GGGCGACGAC
     Cl56    AACAGCAC GCATAACTGA AGAGTACGAA AATGGGGTTG AAGGTTTCTT AAAATTTGCT AAAGATAATG CATCCGACAA TGATGGACTA TACTTTTGTC CTTGCGTTAA ATGTTTGAAT GGGCGACGAC
     Cl42    AACAGCAC GCATAACTGA AGAGTACGAA AATGGGGTTG AAGGTTTCTT AAAATTTGCT AAAGATAATG CATCCGACAA TGGTGGACTA TACTTTTGTC CTTGTGTTAA ATGTTTGAAT GGGCGACGAC
     Cl38    AACAGCAC GCATAACTGA AGAGTACGAA AATGGGGTTG AAGGTTTCTT AAAATTTGCT AAAGATAATG CATCCGACAA TGGTGGACTA TACTTTTGTC CTTGTGTTAA ATGTTTGAAT GGGCGACGAC
     Cl39    AACAGCAC GCATAACTGA AGAGTACGAA AATGGGGTTG AAGGTTTCTT AAAATTTGCT AAAGATAATG CATCCGACAA TGGTGGACTA TACTTTTGTC CTTGTGTTAA ATGTTTGAAT GGGCGACGAC
Consensus  ..AACAGCAC GCATAACTGA AGAGTACGAA AATGGGGTTG AAGGTTTCTT AAAATTTGCT AAAGATAATG CATCCGACAA TGgTGGACTA TACTTTTGTC CTTGtGTTAA ATGTTTGAAT GGGCGACGAC

           131                                                                                                                            260
     gDNA  AATGTTTGGA TGACATTAGA ACACACCTTA TCTGTGATGG TATCTGTCCT ACTTATACAA AATGGATATG GCATGGTGAG TTACCAGAAA TGTCATCAAC CCCTCCAACT GCTCCAACTG ATGAACAAGT
     Cl37  AATGTTTGGA TGACATTAGA ACACACCTTA TCTGTGATGG TATCTGTCCT ACTTATACAA AATGGATATG GCATGGTGAG TTACCAGAAA TGTCATCAAC CCCTCCAACT GCTCCAACTG ATGAACAAGT
     Cl56  AATGTTTGGA TGACATTAGA ACACACCTTA TCTGTGATGG TATCTGTCCT ACTTATACAA AATGGATATG GCATGGTGAG TTACCAGAAA TGTCATCAAC CCCTCCAACT GCTCCAACTG ATGAACAAGT
     Cl42  AATGTTTGGA TGACATTAGA ACACACCTTA TCTGTGATGG TATCTGTCCT ACTTATACAA AATGGATATG GCATGGTGAG TTACCAGAAA TGTCATCAAC CCCTCCAACT GCTCCAACTG ATGAACAAGT
     Cl38  AATGTTTGGA TGACATTAGA ACACACCTTA TCTGTGATGG TATCTGTCCT ACTTATACAA AATGGATATG GCATGGTGAG TTACCAGAAA TGTCATCAAC CCCTCCAACT GCTCCAACTG ATGAACAAGT
     Cl39  AATGTTTGGA TGACATTAGA ACACACCTTA TCTGTGATGG TATCTGTCCT ACTTATACAA AATGGATATG GCATGGTGAG TTACCAGAAA TGTCATCAAC CCCTCCAACT GCTCCAACTG ATGAACAAGT
Consensus  AATGTTTGGA TGACATTAGA ACACACCTTA TCTGTGATGG TATCTGTCCT ACTTATACAA AATGGATATG GCATGGTGAG TTACCAGAAA TGTCATCAAC CCCTCCAACT GCTCCAACTG ATGAACAAGT

                                                                                                       (*)
           261                                                                                                                            390
     gDNA  CGGTGATCAA ATAGAAGACA TGCTACGTGA TCTTGGACAA GAGGGTTTTA GGCAAGCAAA TGCACCGTAT TATGACACCT TACATAATGA TTCAAAGATT CCATTGTTTA TTGGATGCAC TAAGTACACA
     Cl37  CGGTGATCAA ATAGAAGACA TGCTACGTGA TCTTGGACAA GAGGGTTTTA GGCAAGCAAA TGCACCGTAT TATGACACCT TACATAAT-- ---------- ---------- ---------- ----------
     Cl56  CGGTGATCAA ATAGAAGACA TGCTACGTGA TCTTGGACAA GAGGGTTTTA GGCAAGCAAA TGCACCGTAT TATGACACCT TACATAAT-- ---------- ---------- ---------- ----------
     Cl42  CGGTGATCAA ATAGAAGACA TGCTACGTGA TCTTGGACAA GAGGGTTTTA GGCAAGCAAA TGCACCGTAT TATGACACCT TACATAAT-- ---------- ---------- ---------- ----------
     Cl38  CGGTGATCAA ATAGAAGACA TGCTACGTGA TCTTGGACAA GAGGGTTTTA GGCAAGCAAA TGCACCGTAT TATGACACCT TACATAAT-- ---------- ---------- ---------- ----------
     Cl39  CGGTGATCAA ATAGAAGACA TGCTACGTGA TCTTGGACAA GAGGGTTTTA GGCAAGCAAA TGCACCGTAT TATGACACCT TACATAAT-- ---------- ---------- ---------- ----------
Consensus  CGGTGATCAA ATAGAAGACA TGCTACGTGA TCTTGGACAA GAGGGTTTTA GGCAAGCAAA TGCACCGTAT TATGACACCT TACATAAT.. .......... .......... .......... ..........

           391                                                                                                                            520
     gDNA  CGGTTATCAG GGGTGTTAGC TCTGGTCAAT TTGAAAGCAA GATTTGGGTG GAGTGACAAA AGTTTCAATG AATTACTGTT GTTATTGAAG AATATGCTTC CAGGAGATAA CACGTTGCCA AAGACTCATT
Consensus  .......... .......... .......... .......... .......... .......... .......... .......... .......... .......... .......... .......... ..........

           521                                                                                                                            650
     gDNA  ACGAGGCAAA GAAGATATTA TGTCCTGTTG GAATGGAATA CCAAAAAATA CATGCTTGCC GTAATGATTG CATTTTGTAT AGACATGAGT TTGCTGAATT GCGCAACTGC CCTACATGTG GGGTGTCACG
Consensus  .......... .......... .......... .......... .......... .......... .......... .......... .......... .......... .......... .......... ..........

           651                                                                                                                            780
     gDNA  CTACAAAGTG GGTTCTGGCG CTTCCAGTGA AGCTGGATCC ACATACATTG ATCGGCCAGC AAAAGTGTGT TGGTATCTTC CAGTAATACC AAGGTTTAAG CGATTGTTTG CTAATGCAGA AGATGCAAAA
Consensus  .......... .......... .......... .......... .......... .......... .......... .......... .......... .......... .......... .......... ..........

           781                                                                                                                            910
     gDNA  AACCTAACAT GGCATGTTGA TGGTAGGACC AAAGATGGAT TGCTCCGTCA TCCTGCTGAT TCTCCTCAGT GGAAGAAAGT TGATCAGTTG TATCCAGTGT TTGCCGAAGA TCCCAGAAAC CTAAGGGTTG
Consensus  .......... .......... .......... .......... .......... .......... .......... .......... .......... .......... .......... .......... ..........

           911                                                                                                                           1040
     gDNA  GTCTCGCATC GGATGGAATG AATCCATTTC GAAGCTTAAG TTGCAATCAT AGTTCGTGGC CTGTTTTGTT GATCATTTAC AACCTGCCTC CTTGGTTGTG CATCAAGCGG AAGTACATAA TGATGTCTAT
Consensus  .......... .......... .......... .......... .......... .......... .......... .......... .......... .......... .......... .......... ..........

           1041                                                                                                                          1170
     gDNA  GATGATAGCC GGTCCAAGAC AACCAGGAAA TGACATTGAT GTGTATCTTG CTCCCTTGAT TGAAGACCTA ACAAAATTGT GGGTAGAAGG GGTTGATGTG TATGATGGGA ATGCTCATGA GTCCTTCAGG
Consensus  .......... .......... .......... .......... .......... .......... .......... .......... .......... .......... .......... .......... ..........

           1171                                                                                                                          1300
     gDNA  TTGCGAGCTA TGATTTTCTG CACCATTAAT GACTTTCCAG CATATGGGAA TTTGAGTGGA TATAGTGTGA AAGGCCACCT TGCTTGTCCC ATTTGTGAGA AAGACACAAC TTACCTCCAA TTAAAGCATG
Consensus  .......... .......... .......... .......... .......... .......... .......... .......... .......... .......... .......... .......... ..........

           1301                                                                                                                          1430
     gDNA  GCAAAAAAAC TGTATATACA AGACACCGTA GATTTTTACA ACCTTTTCAC CCATACAGGC GACTGAAGAA AGCGTTTGAT GGGACATCTG AGAATGACAG TGCATCAATT CCTTTGTCAG GTGTTGAAGT
Consensus  .......... .......... .......... .......... .......... .......... .......... .......... .......... .......... .......... .......... ..........

           1431                                                                                                                          1560
     gDNA  TTTTGATCGT GTGAAGAACA TTTGCAATAT ATATGGGAAG ACACAAAAGA AAGATGGCGC TCCCAAAAAC ATTTGGAAGA AAAGGTCCAT CTTCTTTGAT CTTCCATACT GGTGCAACTT AGATGTGAGA
Consensus  .......... .......... .......... .......... .......... .......... .......... .......... .......... .......... .......... .......... ..........
```

```
          1561                                                                                                                        1690
     gDNA CATTGTTTAG ATGTGATGCA TGTTGAAAAA AATGTCTGTG ACAGTTTGGT TGGCACACTG CTTAACATTA AAGGGAAGAC AAAAGATGGT TTGAAATGTC GTCAAGATTT AGTGGAAATG GGAGTACGAC
Consensus .......... .......... .......... .......... .......... .......... .......... .......... .......... .......... .......... .......... ..........

          1691                                                                                                                        1820
     gDNA ACCAGTTGCA TCCTGTGTCA AAAGGTCTTC GAACGTATTT GCCGCCCGCA TGTCATACGA TGTCAACATA TGAGAAAAAA AGTTTTTGTC ATTGTCTGAA AAATGTCAAA GTCCCACAAG GATACTCTTC
Consensus .......... .......... .......... .......... .......... .......... .......... .......... .......... .......... .......... .......... ..........

          1821                                                                                                                        1950
     gDNA AAATATCAAG AGCCTTGTAT CAGTGGATGA AATGAAATTG GTGGGGTTGA AGTCCCATGA TTGTCACGTT TTGATGCAAC AATTATTGCC TGTTGCCATT CGTGGAATAT TGCCTGACAA AGTTAGGGTT
Consensus .......... .......... .......... .......... .......... .......... .......... .......... .......... .......... .......... .......... ..........

          1951                                                                                                                        2080
     gDNA GCAATAACTC GATTGTGTTT TTTCTTTAAT GCAATCTGTA GCAAAGTGAT TGACCCTAAA CAGTTGGATG ATTTGGAAAA TGAGGCTGCC ATTATCATTT GTCAATTGGA GATGTACTTT CCCCCAACTT
Consensus .......... .......... .......... .......... .......... .......... .......... .......... .......... .......... .......... .......... ..........

          2081                                                                                                                        2210
     gDNA TTTTTGACAT AATGATTCAC TTACTTGTTC ATCTTGTTCG AGAAATACGT TTGTGTGGGC CTGTATATTT GCGGTGGATG TATCCGGTTG AGCGGTACAT GAAGGTGTTG AAAAGTTACA CGAAGAATCA
Consensus .......... .......... .......... .......... .......... .......... .......... .......... .......... .......... .......... .......... ..........

          2211                                                                                                                        2340
     gDNA ATATAGGCCA GAAGCAAGCA TTGTTGAAAG GTACGTGGCA GAAGAAGCTA TTGAGTTTTG CTCTACTTAC ATCGAAGATG CATCACCTGT TGGTATTCCT GAAAGTCGTC ATGAAGCTAC ACGACAAGGT
Consensus .......... .......... .......... .......... .......... .......... .......... .......... .......... .......... .......... .......... ..........

          2341                                                                                                                        2470
     gDNA AGGGGAACGC GAGGATTCAA TGTTGTAACC ATGGATCGCC AGAAACTATC ACAAGCGCAT TTGTATGTAC TTAACAACAC AGCTGAGGTA ATACCATACA TAGATGCTCA CAAAGAATAT GTGGCAGCTT
Consensus .......... .......... .......... .......... .......... .......... .......... .......... .......... .......... .......... .......... ..........

          2471                                                                                                                        2600
     gDNA CTCACCCAAA CATGAATATG ATGAGGGTGT TGCAGGAACA CAATAGAAGT TTCATTAATT GGTTTAGAAA TACAATATTT GCTAGCGACA GTGCTTCTAA GACATTATCA TTACTAGCTG TTGGGCCGAA
Consensus .......... .......... .......... .......... .......... .......... .......... .......... .......... .......... .......... .......... ..........

          2601                                                                                                                        2730
     gDNA TCTTAATGTC CTCACTTGGA AGGGTTATGA CATCAACAAT TATTCCTTCT ACACAAAGTC ACAAGATGAT AAAAGTACCG TGCAAAATAG TGGGGTCATG ATTGATGCTC ATTCAGACCA CTTTAGTCGT
Consensus .......... .......... .......... .......... .......... .......... .......... .......... .......... .......... .......... .......... ..........

          2731                                                                                                                        2860
     gDNA GCATCGGATA ACAATCCTAT TCGAGCTTCC ATGGCTTATT ATGGAGTCAT AACCGATATC TGGGAGCTAG ACTATGGTGA ATTTAGAGTG CCTGTTTTCA AGTGCCAATG GGTTAATGGA AATGTCGGAG
Consensus .......... .......... .......... .......... .......... .......... .......... .......... .......... .......... .......... .......... ..........

          2861                                                                                                                        2990
     gDNA TCCGTCAAGA CAAATTGGGT TTTACTTTGG TTGACCTTCA AAGGATTGGT TACAAGGACG AGCCTTTCAT CATGGCAGCA CAAGCAAGAC AAGTGTTTTA TGTAGAAGAT CCTAGTGACT CAACATGGTC
Consensus .......... .......... .......... .......... .......... .......... .......... .......... .......... .......... .......... .......... ..........

          2991                                                                                                                        3120
     gDNA AGTTGTACTT CAAGGGAAAA CAAGTGGTAT CCCTGCCGAT ACTGACCAAG CAACCCTTGA TGTTAACGAA ATCCCTACGT TTGCACAACA AATGCCTTCG ATAAATGCTG AAAACGACGA CGATGATGTG
Consensus .......... .......... .......... .......... .......... .......... .......... .......... .......... .......... .......... .......... ..........

          3121                                                                                                                        3250
     gDNA TATGCAAATC GTATCGATCA TGATGAAGGT TTATGGGAAA ATATGGCAAC TTAAATGCGG TAAATAAGAC AATAATGTTA AACTTGTCAA TAGTCATTTT TTATTTAATG TTTCTTCTCT TTCATTACAC
Consensus .......... .......... .......... .......... .......... .......... .......... .......... .......... .......... .......... .......... ..........

          3251                                                                                                                        3380
     gDNA TTTCATACTT ACATGTTGTT CTAATCTAAC ATTTGGTTTC TTAATATTAT TGCAGAACCC ATGGCAACAC CCCCGACATC CCCTCCACCT CCTACATCCC CTCCACCTGC TGATTCACCA AGCGCAATCT
Consensus .......... .......... .......... .......... .......... .......... .......... .......... .......... .......... .......... .......... ..........

          3381                                                                                                                        3510
     gDNA CAAAACCGAA GACTCGACAA GCAACCAGGT TGAGGAAATT GACTGCAAGA ACCTTGGATC AACCACGACC AATTGTCAAC GTCAACCCCG TTACTGGTCG AGGTTCTGGT TCGGAAAAAG ATAAATTTCA
Consensus .......... .......... .......... .......... .......... .......... .......... .......... .......... .......... .......... .......... ..........

          3511                                                                                                                        3640
     gDNA CAGTTACTTG GGGGTAGTGG CACGGGAGAA AATCCCTATT GTGCATTCAT CTTGGAAAGT TGTCCCAGAA TCACTTAAAA ATATTGTATG GAATGACATT TTGGTAAGCG CACTTAAATT AAAAATTTGA
Consensus .......... .......... .......... .......... .......... .......... .......... .......... .......... .......... .......... .......... ..........

          3641                                                                                                                        3770
     gDNA TTTCATTTGT GTCTCCTTTA GGCCAAATTG TCATTTTTAT CATTAGTGTA TGACAAATTA TTTGTTATTT CAGGGAAAAT TTGACATCCC GGAAGGAACT GCTGCCAAGA AGAAGGTCAT GTCTACTGTT
Consensus .......... .......... .......... .......... .......... .......... .......... .......... .......... .......... .......... .......... ..........
```

```
           3771                                                                                                                                 3900
     gDNA  GCGACTAGAT GGAGGCAATT TAAGTCCTCC CTGACCAGTA GATATATATA TGCTGAGAAA CATGGTGAAG ATAACCCTGA TGCAGCTTCT AAGTATGGTA TGGAGCAGCA AACATGGGAG CAATTTGCAA
Consensus  .......... .......... .......... .......... .......... .......... .......... .......... .......... .......... .......... .......... ..........

           3901                                                                                                                                 4030
     gDNA  AGAGTCGACA GACCCCAACT TGGCAGGTTT GAATTGACTT TATATTTCAC AAGTTTTGTT ACTTAAATTT TGTTAACATT ATAACACAAT GCATTGTTTT TGTTACTTCG GACAGGGAAT TCGGAAAAAA
Consensus  .......... .......... .......... .......... .......... .......... .......... .......... .......... .......... .......... .......... ..........

           4031                                                                                                                                 4160
     gDNA  GCACAGGAGA TCCAAAAATT CAATGACTCC CCTCATTTAT TGTCTCGTGG AGGGTATGAA CTTATGGAAA AAAAATTGAT GGAAGAGAAA ATGAAGACAA GACAAAGGCA AGCTGAGTGT ACAGAAAATA
Consensus  .......... .......... .......... .......... .......... .......... .......... .......... .......... .......... .......... .......... ..........

           4161                                                                                                                                 4290
     gDNA  CACCGATGGT CGTAGACCCT CCATCCCCAA TTGCAAGACA TGTTAAGTGG AAGATGGCTA GAACAAACAA ATATGGAAAA ATGACATCTG CAGCAGCTCA ACAAATCTCT GACAAAATTG TAAGTGCAAT
Consensus  .......... .......... .......... .......... .......... .......... .......... .......... .......... .......... .......... .......... ..........

           4291                                                                                                                                 4420
     gDNA  TAAAGCCTTC GTGGCAATTT GAATAGGTAG ATGGTATATC ATTTAACTTT GATCTGATTT TTGGCTATTT ATGACAGGAT GAATTAGAAG AACAAAGCAC ACAAGGTACG TTTGTGCCGC ATGGCCGGAA
Consensus  .......... .......... .......... .......... .......... .......... .......... .......... .......... .......... .......... .......... ..........

           4421                                                                                                                                 4550
     gDNA  CGACATATTG AACACTGCGC TTGGCCGTGA AGAGCATCCT GGTCGTGTCC TTGCTGCTGG ACATGGTGTC ACCATTAGTA GTTACTTTGG ACAGCGTTCA AGTGCCTCTA ATAGTTCTGC TGCTACGATA
Consensus  .......... .......... .......... .......... .......... .......... .......... .......... .......... .......... .......... .......... ..........

           4551                                                                                                                                 4680
     gDNA  ACCCCGGATC AGTTGGTTCA AATCATAGGT AATCTCAAGC AAGAGTGGAC AAAAGAGGTA GAAGATGCAA GCAAACAAAA AATGGACATG CTGCAAAAGG AGTTGGATGC AATCAAGACT GAGTTGTCCC
Consensus  .......... .......... .......... .......... .......... .......... .......... .......... .......... .......... .......... .......... ..........

           4681                                                                                                                                 4810
     gDNA  AAATGCAAAC TCAACAGTCA GCCCCTGTAC AACCGGCTAA CCCTAATGTG TTGATTGCAC GTGTTAGCAC CAAAGAAAGT TGTGCAGAAG CTGTTGCAAA TGTTGTTGCT GGGGACCCAT CTGCGGTTGA
Consensus  .......... .......... .......... .......... .......... .......... .......... .......... .......... .......... .......... .......... ..........

           4811                                                                                                                                 4940
     gDNA  GGAGAATACC ATGGGATTGT ATGTTGTTTG TGGCGACAGT AAACAATTGG TGGCCTTAGG AAAGGTGTAT CAAGTTGGCG GCATGATACA CAATGTTCCT TACGCAGATG AAGTCGTGAG GGTTTCTGTG
Consensus  .......... .......... .......... .......... .......... .......... .......... .......... .......... .......... .......... .......... ..........

           4941                                                                                                                                 5070
     gDNA  ATTACTGTTT ATGATGGTGA TGCAAGGGTC CCAATTCCCA CACCTGAGAT TGAATACGTT AGGGAGGCCA TGAACACATT CATTGGCTGG CCAACTAATC TTGTCAAACC TTTCTCCGCT GTAAGTGAAA
Consensus  .......... .......... .......... .......... .......... .......... .......... .......... .......... .......... .......... .......... ..........

           5071                                                                                                                                 5200
     gDNA  TGATTTTCCT TGTGGTTCCC CTAAATTAAA TGGCATATTC CAATAATGGT TGCTAAATTA AATTTAATAT TTATTTTATA AAACTATATA GGATTCCAAT CAAGATGTAA GGAATCCAAA AGGACATGTT
Consensus  .......... .......... .......... .......... .......... .......... .......... .......... .......... .......... .......... .......... ..........

           5201                                                                                                                                 5330
     gDNA  GATCGGTCAA ATGCAGGTGA TGCAATGGAT CCACTTGGAG AAATCATGAA AATACTTTAT GAAGTGTATA TGAATCCAGT GGAACTCCCG TGGGAGGCTA GCCGATTTGG AATTCCAAAT ATAGATGCCA
Consensus  .......... .......... .......... .......... .......... .......... .......... .......... .......... .......... .......... .......... ..........

           5331                                                                                                                                 5460
     gDNA  AATTTTACAT CACACATGCT GATATGGCTG AAATAATATC AGGTCACAAG TGTTTAAACA TTTCTATACT GCAACTATGG ATGATGTAAG TCATTTAATT ACAACCTTTA ACCCTAAATG TTATCATAAG
Consensus  .......... .......... .......... .......... .......... .......... .......... .......... .......... .......... .......... .......... ..........

           5461                                                                                                                                 5590
     gDNA  CAACAAATTG TAAATGTAAT ACATTTGTGG TATAATGATT GTATTGTCTT TCAAATAGGT ATTTGGATGA GTGTGCTACA AGCAGAGGTG ATGGCTCAGT GTATGGCTTC CTTGAGCCTC AATCAATACA
Consensus  .......... .......... .......... .......... .......... .......... .......... .......... .......... .......... .......... .......... ..........

           5591                                                                                                                                 5720
     gDNA  CATTGGTAAG GAGGACCGTC AACAATGTCA ACTTTATATT GAGACATGGG TGAAGGAATC ACAACGATGC TTGTACTTAG GAGCATACTT GCATCAGTAA GTTAAATTTT TTTGTGGCAT TTAACAAATG
Consensus  .......... .......... .......... .......... .......... .......... .......... .......... .......... .......... .......... .......... ..........

           5721                                                                                                                                 5850
     gDNA  TTATGATTTC TAAACTTGCT AATTATAATC ATCAACTTCA GGTCACATTG GCAACTATTT GTTCTCTGTC CTAGGGAAAA CATGGTTGTT TGGTTTTGTT CGTTGCGAAA GAAGCCTGAT GTTAACATAA
Consensus  .......... .......... .......... .......... .......... .......... .......... .......... .......... .......... .......... .......... ..........

           5851                                                                                                                                 5980
     gDNA  AAGCCGTAAT AAATAGGTAC GAGTGTAATG TACTTAAAGA TAGCCGAGCT GTCATTGTTG TTGAATATGC AGCGTAAATG TTTGTTATAA GTAACATCAT ATATGATTTC GTTTACTAGT GCAATGAAGA
Consensus  .......... .......... .......... .......... .......... .......... .......... .......... .......... .......... .......... .......... ..........
```

```
          5981                                                                                                                              6110
     gDNA CAATAAGTAG TTCTTTGGAA GGCATGTCTC AGCAAGGTCC ACCTCGGTGG ATTGAACCCA AGGTTAGATG GTTGTTTAGA TGAACCCCTA TGTAATTTTT AAAGGGTACA AGTGATAATA GTTATTTTCA
Consensus .......... .......... .......... .......... .......... .......... .......... .......... .......... .......... .......... .......... ..........

          6111                                                                                                                              6240
     gDNA CTGATAAATA TAGAGTCATG TTCAAAGTGG AGGGTACGAG TGTGGATACT ATGTGATGCA TTGGATGTGG TGCATCGTTA GTGGTCGTTT GAAGGATGAC TGGAACAGGG TATATATACA AAGTCTAATT
Consensus .......... .......... .......... .......... .......... .......... .......... .......... .......... .......... .......... .......... ..........

                                                      (*)
          6241                                                                                                                              6370
     gDNA TCAATTTTCA TGTTAGTTGA TATTTGTTTA ATTATTAATA ATGTCTTCCA TTGTTAATTT TGTAGTGGTT CTCGGATGGA TCAGCATTAG ATGTGGAGGC CATGACAATA ATTCGAAAGA ATTGGGCAAC
     Cl37 ---------- ---------- ---------- ---------- --GTCTTCCA TTGTTAATTT TGTAGTGGTT CTCGGATGGA TCAGCATTAG ATGTGGAGGC CATGACAATA ATTCGAAAGA ATTGGGCAAC
     Cl56 ---------- ---------- ---------- ---------- --GTCTTCCA TTGTTAATTT TGTAGTGGTT CTCGGATGGA TCAGCATTAG ATGTGGAGGC CATGACAATA ATTCGAAAGA ATTGGGCAAC
     Cl42 ---------- ---------- ---------- ---------- --GTCTTCCA TTGTTAATTT TGTAGTGGTT CTCGGATGGA TCAGCATTAG ATGTGGAGGC CATGACAATA ATTCGAAAGA ATTGGGCAAC
     Cl38 ---------- ---------- ---------- ---------- --GTCTTCCA TTGTTAATTT TGTAGTGGTT CTCGGATGGA TCAGCATTAG ATGTGGAGGC CATGACAATA ATCCGAAAGA ATTGGGCAAC
     Cl39 ---------- ---------- ---------- ---------- --GTCTTCCA TTGTTAATTT TGTAGTGGTT CTCGGATGGA TCAGCATTAG ATGTGGAGGC CATGACAATA ATTCGAAAGA ATTGGGCAAC
Consensus .......... .......... .......... .......... ..GTCTTCCA TTGTTAATTT TGTAGTGGTT CTCGGATGGA TCAGCATTAG ATGTGGAGGC CATGACAATA ATtCGAAAGA ATTGGGCAAC

          6371                                                                                                                              6500
     gDNA TTACTTTTTA GCTATTAGAA ATAACAGATG CTAAATATGA TGTAGATTAT TATGAATGAC TACATTTTCC TTTAATGACA CCCTTTAGTG GTATATTTTA ATGAATTGTT TCATGTCACA TTAATGTTTT
     Cl37 TTACTTTTTA GCTATTAGAA ATAACAGATG CTAAATATGA TGTAGATTAT TATGAATGAC TACATTTTCC TTTAATGACA CCCTTTAGTG GTATATTTTA ATGAATTGTT TCATGTCACA TTAATGTTTT
     Cl56 TTACTTTTTA GCTATTAGAA ATAACAGATG CTAAATATGA TGTAGATTAT TATGAATGAC TACATTTTCC TTTAATGACA CCCTTTAGTG GTATATTTTA ATGAATTGTT TCATGTCACA TTAATGTTTT
     Cl42 TTACTTTTTA GCTATTAGAA ATAACAGATG CTAAATATGA TGTAGATTAT TATGAATGAC TACATTTTCC TTTAATGACA CCCTTTAGTG GTATATTTTA ATGAATTGTT TCATGTCACA TTAATGTTTT
     Cl38 TTACTTTTTA GCTATTAGAA ATAACAGATG CTAAATATGA TGTAGATTAT TATGAATGAC TACATTTTCC TTTAATGACA CCCTTTAGTG GTATATTTTA ATGAATTGTT TCATGTCACA TTAATGTTTT
     Cl39 TTACTTTTTA GCTATTAGAA ATAACAGATG CTAAATATGA TGTAGATTAT TATGAATGAC TACATTTTCC TTTAATGACA CCCTTTAGTG GTATATTTTA ATGAATTGTT TCATGTCACA TTAATGTTTT
Consensus TTACTTTTTA GCTATTAGAA ATAACAGATG CTAAATATGA TGTAGATTAT TATGAATGAC TACATTTTCC TTTAATGACA CCCTTTAGTG GTATATTTTA ATGAATTGTT TCATGTCACA TTAATGTTTT

          6501                                                                                                                              6630
     gDNA TTAAAAACCT ACGTAATGGT TTAGTAAGGA GTTTATGTAT TCTGAAATTG TTTTGGTTTG TTGTAGTCTC GTATTAGAAA ATATATATTT TGATAGGATG AGTGCAGTTT TCTTAGAAA- TTGTACTCCT
     Cl37 TtAAAAACCT ACGTAATGGT TTAGTAAGGA GTTTATGTAT TCTGAAATTG TTTTGGTTTG TTGTAGTCTC GTATTAGAAA ATATATATTT TGATAGGATG AGTGCAGTTT TCTTAGAAA- TTGTACTCCT
     Cl56 TTAAAAACCT ACGTAATGGT TTAGTAAGGA GTTTATGTAT TCTGAAATTG TTTTGGTTTG TTGTAGTCTC GTATTAGAAA ATATATATTT TGATAGGATG AGTGCAGTTT TCTTAGAAA- TTGTACTCCT
     Cl42 TTAAAAACCT ACGTAATGGT TTAGTAAGGA GTTTATGTAT TCTGAAATTG TTTTGGTTTG TTGTAGTCTC GTATTAGAAA ATATATATTT TGATAGGATG AGTGCAGTTT TCTTAGAAA- TTGTACTCCT
     Cl38 TTAAAAACCT ACGTAATGGT TTAGTAAGGA GTTTATGTAT TCTGAGATTG TTTTGGTTTG TTGTAGTCTC GTATTAGAAA ATATATATTT TGATAGGATG AGTGCAGTTT TCTTAGAAAA TTGTACTCCT
     Cl39 TTAAAAACCT ACGTAATGGT TTAGTAAGGA GTTTATGTAT TCTGAAATTG TTTTGGTTTG TTGTAGTCTC GTATTAGAAA ATATATATTT TGATAGGATG AGTGCAGTTT TCTTAGAAA- TTGTACTCCT
Consensus TtAAAAACCT ACGTAATGGT TTAGTAAGGA GTTTATGTAT TCTGAaATTG TTTTGGTTTG TTGTAGTCTC GTATTAGAAA ATATATATTT TGATAGGATG AGTGCAGTTT TCTTAGAAA. TTGTACTCCT

          6631                                                                                                                              6760
     gDNA ACTATGTATG TACTGCACAG TTGCACCTTC CATCAACTTA ACAACACGTA GTTACTTGTG ATAGCACTTG TATTATTTTT ATTCCCATTA CCGTAAGGCC TGAAGTGGTA AGTAGATAAT GCCCTGCGTG
     Cl37 ACTATGTATG TACTGCACAG TTGCACCTTC CATCAACTTA ACAACACGTA GTTACTTGTG ATAGCACTTG TATTATTTTT ATTCCCATTA CCGTAAGGCC TGAAGTGGTA AGTAGATAAT GCCCTGCGTG
     Cl56 ACTATGTATG TACTGCACAG TTGCACCTTC CATCAACTTA ACAACACGTA GTTACTTGTG ATAGCACTTG TATTATTTTT ATTCCCATTA CCGTAAGGCC TGAAGTGGTA AGTAGATAAT GCCCTGCGTG
     Cl42 ACTATGTATG TACTGCACAG TTGCACCTTC CATCAACTTA ACAACACGTA GTTACTTGTG ATAGCACTTG TATTATTTTT ATTCCCATTA CCGTAAGGCC TGAAGTGGTA AGTAGATAAT GCCCTGCGTG
     Cl38 ACTATGTATG TACTGCACAG TTGCACCTTC CATCAACTTA ACAACACGTA GTTACTTGTG ATAGCACTTG TATTATTTTT ATTCCCATTA CCGTAAGGCC TGAAGTGGTA AGTAGATAAT GCCCTGCGTG
     Cl39 ACTATGTATG TACTGCACAG TTGCACCTTC CATCAACTTA ACAACACGTA GTTACTTGTG ATAGCACTTG TATTATTTTT ATTCCCATTA CCGTAAGGCC TGAAGTGGTA AGTAGATAAT GCCCTGCGTG
Consensus ACTATGTATG TACTGCACAG TTGCACCTTC CATCAACTTA ACAACACGTA GTTACTTGTG ATAGCACTTG TATTATTTTT ATTCCCATTA CCGTAAGGCC TGAAGTGGTA AGTAGATAAT GCCCTGCGTG

          6761                                                                                                                              6890
     gDNA TAGCACATAA TTTCTACTTT GGGTATAGCC TTGCAAGCGA ATGTTGTTGT TTGACCTTCT AGCACAGGAA AAACAATGGT TGCAAAGGCT GTGGCAACTG AAGCCGGAGG AAAAGCAATA TCCCAATAGG
     Cl37 TAGCACATAA TTTCTACTTT GGGTATAGCC TTGCAAGCGA ATGTTGTTGT TTGACCTTCT AGCACAGGAA AAACAATGGT TGCAAAGGCT GTGGCAACTG AAGCCGGAGG aAAAGCAATA TCCCAATAG-
     Cl56 TAGCACATAA TTTCTACTTT GGGTATAGCC TTGCAAGCGA ATGTTGTTGT TTGACCTTCT AGCACAGGAA AAACAATGGT TGCAAAGGCT GTGGCAACTG AAGCCGGAGG AAAAGCAATA TCCCAATAG-
     Cl42 TAGCACATAA TTTCTACTTT GGGTATAGCC TTGCAAGCGA ATGTTGTTGT TTGACCTTCT AGCACAGGAA AAACAATGGT TGCAAAGGCT GTGGCAACTG AAGCCGGAGG AAAAGCAATA TCCCAATAG-
     Cl38 TAGCACATAA TTCCTACTTT GGGTATAGCC TTGCAAGCGA ATGTTGTTGT TTGACCTTCT AGCACAGGAA AAACAATGGT TGCAAAGGCT GTGGCAACTG AAGCCGGAGG AAAAGCAATA TCCCAATAG-
     Cl39 TAGCACATAA TTTCTACTTT GGGTATAGCC TTGCAAGCGA ATGTTGTTGT TTGACCTTCT AGCACAGGAA AAACAATGGT TGCAAAGGCT GTGGCAACTG AAGCCGGAGG AAAAGCAATA TCCCAATAG-
Consensus TAGCACATAA TTtCTACTTT GGGTATAGCC TTGCAAGCGA ATGTTGTTGT TTGACCTTCT AGCACAGGAA AAACAATGGT TGCAAAGGCT GTGGCAACTG AAGCCGGAGG aAAAGCAATA TCCCAATAG-

          6891                                                                                                                              7020
     gDNA TATGTTAACA GATTATTTTA ACTCTTTGAA ATGTTTGCAG TCAGCTGAGT TGACTTCCAT GTCAATGTAA TCATGTTCTG CAGGGTCACA TGCAAAGAGT GTAGTAGCCA TTTCAGCCTC TGAGGTACAC
     Cl37 ---------- ---------- ---------- ---------- ---------- ---------- ---------- ---------- ---GGTCACA TGCAAAGaGT GTAGTAGCCA TTTCAGCCTC TGAG------
     Cl56 ---------- ---------- ---------- ---------- ---------- ---------- ---------- ---------- ---GGTCACA TGCAAAGAGT GTAGTAGCCA TTTCAGCCTC TGAG------
     Cl42 ---------- ---------- ---------- ---------- ---------- ---------- ---------- ---------- ---GGTCACA TGCAAAGAGT GTAGTAGCCA TTTCAGCCTC TGAG------
     Cl38 ---------- ---------- ---------- ---------- ---------- ---------- ---------- ---------- ---GGTCACA TGCAAAGAGT GTAGTAGCCA TTTCAGCCTC TGAG------
     Cl39 ---------- ---------- ---------- ---------- ---------- ---------- ---------- ---------- ---GGTCACA TGCAAAGAGT GTAGTAGCCA TTTCAGCCTC TGAG------
Consensus .......... .......... .......... .......... .......... .......... .......... .......... ...GGTCACA TGCAAAGaGT GTAGTAGCCA TTTCAGCCTC TGAG......
```

```
           7021                                                                                                                                    7150
     gDNA  AACATAGACT TTATACTCTT TATAATAACT AATCTTATTT CAATCTTTTT CATTCAATGA AATGAATGAG ATAGAATTTT ATTTGATTTA CCAGTGCTAA GGAAGCGCAT GAAGTAAAAA TAATGTGTAC
     Cl37  ---------- ---------- ---------- ---------- ---------- ---------- ---------- ---------- ---------- ----TGCTAA GGAAGCGCAT GAAGTAAAAA TAATGTGTAC
     Cl56  ---------- ---------- ---------- ---------- ---------- ---------- ---------- ---------- ---------- ----TGCTAA GGAAGCGCAT GAAGTAAAAA TAATGTGTAC
     Cl42  ---------- ---------- ---------- ---------- ---------- ---------- ---------- ---------- ---------- ---------- ---------- ---------- ----------
     Cl38  ---------- ---------- ---------- ---------- ---------- ---------- ---------- ---------- ---------- ----TGCTAA GGAAGCGCAT GAAGTAAAAA TAATGTGTAC
     Cl39  ---------- ---------- ---------- ---------- ---------- ---------- ---------- ---------- ---------- ---------- ---------- ---------- ----------
Consensus  .......... .......... .......... .......... .......... .......... .......... .......... .......... ....tgctaa ggaagcgcat gaagtaaaaa taatgtgtac

           7151                                                                                                                                    7280
     gDNA  ATATCACTTC TCTCAAAGCC CAGATTAATT TTACTTTTAG AGGTTAGCAT AGATTATTCC CCCTTCTTAG TATAAAAATA AATAAATTAT CTCCTTGCTC TTCTTTAAAT TATCTCCCAG CATATGTAAT
     Cl37  ATATCACTTC TCTCAAAGCC CAGATTAATT TTACTTTTAG AG-------- ---------- ---------- ---------- ---------- ---------- ---------- ---------- ----------
     Cl56  ATATCACTTC TCTCAAAGCC CAGATTAATT TTACTTTTAG AG-------- ---------- ---------- ---------- ---------- ---------- ---------- ---------- ----------
     Cl42  ---------- ---------- ---------- ---------- ---------- ---------- ---------- ---------- ---------- ---------- ---------- ---------- ----------
     Cl38  ATATCACTTC TCTCAAAGCC CAGATTAATT TTACTTTTAG AG-------- ---------- ---------- ---------- ---------- ---------- ---------- ---------- ----------
     Cl39  ---------- ---------- ---------- ---------- ---------- ---------- ---------- ---------- ---------- ---------- ---------- ---------- ----------
Consensus  atatcacttc tctcaaagcc cagattaatt ttacttttag ag........ .......... .......... .......... .......... .......... .......... .......... ..........

           7281                                                                                                                                    7410
     gDNA  ACTTAATCCT TGGCACAGTA TCTTTTTTCT TTTCACTCAC TCCTTGTTTC TGCTGAAAGC TAATTGGTTG AAACTCATTT TTTAGTGAGT GATACAGATG AGGATGTCTT GCAAATATTT TTTAAGGAGA
     Cl37  ---------- ---------- ---------- ---------- ---------- ---------- ---------- ---------- -----TGAGT GATACAGATG AGGATGTCTT GCAAATATTT TTTAAGGAGA
     Cl56  ---------- ---------- ---------- ---------- ---------- ---------- ---------- ---------- -----TGAGT GATACAGATG AGGATGTCTT GCAAATATTT TTTAAGGAGA
     Cl42  ---------- ---------- ---------- ---------- ---------- ---------- ---------- ---------- -----TGAGT GATACAGATG AGGATGTCTT GCAAATATTT TTTAAGGAGA
     Cl38  ---------- ---------- ---------- ---------- ---------- ---------- ---------- ---------- -----TGAGT GATACAGATG AGGATGTCTT GCAAATATTT TTTAAGGAGA
     Cl39  ---------- ---------- ---------- ---------- ---------- ---------- ---------- ---------- -----TGAGT GATACAGATG AGGATGTCTT GCAAATATTT TT-AAGGAGA
Consensus  .......... .......... .......... .......... .......... .......... .......... .......... .....TGAGT GATACAGATG AGGATGTCTT GCAAATATTT TTtAAGGAGA

           7411                                                                                                                                    7540
     gDNA  GAGAATTAAA TGGGGATTTT ATATCAAGAG CTTCCGATTT ATTATGGAGA AGAGATTTCA GAAGTTCTGG TGATT-ATGA TATTAGCGAG CTCACCGACA ACACTTCTCA ACAAATAGAG CAGGTGCAGC
     Cl37  GAGAATTAAA TGGGGATTTT ATATCAAGAG CTTCCGATTT ATTATGGAGA AGAGATTTCA GAAGTTCTGG TGATT-ATGA TATTAGCGAG CTCACCGACA ACACTTCTCA ACAAATAGAG CAG-------
     Cl56  GAGAATTAAA TGGGGATTTT ATATCAAGAG CTTCCGATTT ATTATGGAGA AGAGATTTCA GAAGTTCTGG TGATTTATGA TATTAGCGAG CTCACCGACA ACACTTCTCA ACAAATAGAG CAG-------
     Cl42  GAGTATTAAA TGGGGATTTT ATATCAAGAG CTTCCGATTT ATTATGGAGA AGAGATTTCA GAAGTTCTGG TGATT-ATGA TATTAGCGAG CTCACCGACA TCACTTCTCA ACAAATAGAG CAG-------
     Cl38  GAGAATTAAA TGGGGATTTT ATAGCAAGAG CTTCCGATTT ATTATGGAGA AGAGATTTCA GAAGTTCTGG TGATT-ATGA TATTAGCGAG CTCACCGACA ACACTTCTCA ACAAATAGAG CAG-------
     Cl39  GAGAATTAAA TGGGGATTTT ATATCAAGAG CTTCCGATTT ATTATGGAGA AGAGATTTCA GAAGTTCTGG TGATT-ATGA TATTAGCGAG CTCACCGACA ACACTTCTCA ACAAATAGAG CAG-------
Consensus  GAGaATTAAA TGGGGATTTT ATAtCAAGAG CTTCCGATTT ATTATGGAGA AGAGATTTCA GAAGTTCTGG TGATT.ATGA TATTAGCGAG CTCACCGACA aCACTTCTCA ACAAATAGAG CAG.......

           7541                                                                                                                                    7670
     gDNA  AGACTCATTA TATCTAATTT CGAACTATTC CTATCCAATT TTAAAGATTC TATCAAGTGT GTCAGTTTTG GCAATTTGTT GCCTTGTACT GATTCATTGC AAATTTTGTA TATTGATTCG TTGGTCAAAT
Consensus  .......... .......... .......... .......... .......... .......... .......... .......... .......... .......... .......... .......... ..........

           7671                                                                                                                                    7800
     gDNA  AATTCAATTT CACGTGTTTT TTATAATATT TGTAGATCAT AGAGACTGAC AGTGATGGTG GTTTGTTGAA ACTTACAAGA ACCCAAGAGT GGCTAACAGG TGACAATTCT CCACCAATAA ACAAGAAGGT
     Cl37  ---------- ---------- ---------- -----ATCAT AGAGACTGAC AGTGATGGTG GTTTGTTGAA ACTTACAAGA ACCCAAGAGT GGCTAACAGG TGACAATTCT CCACCAATAA ACAAGAAGGT
     Cl56  ---------- ---------- ---------- -----ATCAT AGAGACTGAC AGTGATGGTG GTTTGTTGAA ACTTACAAGA ACCCAAGAGT GGCTAACAGG TGACAATTCT CCACCAATAA ACAAGAAGGT
     Cl42  ---------- ---------- ---------- -----ATCAT AGAGACTGAC AGTGATGGTG GTTTGTTGAA ACTTACAAGA ACCCAAGAGT GGCTAACAGG TGACAATTCT CCACCAATAA ACAAGAAGGT
     Cl38  ---------- ---------- ---------- -----ATCAT AGAGACTGAC AGTGATGGTG GTTTGTTGAA ACTTACAAGA ACCCAAGAGT GGCTAACAGG TGACAATTCT CCACCAATAA ACAAGAAGGT
     Cl39  ---------- ---------- ---------- -----ATCAT AGAGACTGAC AGTGATGGTG GTTTGTTGAA ACTTACAAGA ACCCAAGAGT GGCTAACAGG TGACAATTCT CCACCAATAA ACAAGAAGGT
Consensus  .......... .......... .......... .....ATCAT AGAGACTGAC AGTGATGGTG GTTTGTTGAA ACTTACAAGA ACCCAAGAGT GGCTAACAGG TGACAATTCT CCACCAATAA ACAAGAAGGT

           7801                                                                                                                                    7930
     gDNA  GACTGCTAAG GTATGCTTTA GAGTAGTTTT CATTTTACAT CCCAACTTTC ATTTTCTTCT GTATGTTATT ATCTTGTCAG GGAAATTGGA TATGAAATTG ATGTGTTACA ATGCATATTT TATTTTGTCT
     Cl37  GACTGCTAAG ---------- ---------- ---------- ---------- ---------- ---------- ---------- ---------- ---------- ---------- ---------- ----------
     Cl56  GACTGCTAAG ---------- ---------- ---------- ---------- ---------- ---------- ---------- ---------- ---------- ---------- ---------- ----------
     Cl42  GACTGCTAAG ---------- ---------- ---------- ---------- ---------- ---------- ---------- ---------- ---------- ---------- ---------- ----------
     Cl38  GACTGCTAAG ---------- ---------- ---------- ---------- ---------- ---------- ---------- ---------- ---------- ---------- ---------- ----------
     Cl39  GACTGCTAAG ---------- ---------- ---------- ---------- ---------- ---------- ---------- ---------- ---------- ---------- ---------- ----------
Consensus  GACTGCTAAG -......... .......... .......... .......... .......... .......... .......... .......... .......... .......... .......... ..........

           7931                                                                                                                                    8060
     gDNA  AATGTTTTTC AAATTCACCT TCTCTCTGTG GTAAATAAAT AATAACCCAG TCATCCAATA ATGTTGGAAA CTTCATTTCA TATATCAACA GGCATTACAG GACAGCAGTG CAAGACGCAT GAAACTGAAC
     Cl37  ---------- ---------- ---------- ---------- ---------- ---------- ---------- ---------- ---------- -GCATTACAG GACAGCAGTG CAAGACGCAT GAAACTGAAC
     Cl56  ---------- ---------- ---------- ---------- ---------- ---------- ---------- ---------- ---------- -GCATTACAG GACAG---TG CAAGACGCAT GAAACTGAAC
     Cl42  ---------- ---------- ---------- ---------- ---------- ---------- ---------- ---------- ---------- -GCATTACAG GACAGCAGTG CAAGACGCAT GAAACTGAAC
     Cl38  ---------- ---------- ---------- ---------- ---------- ---------- ---------- ---------- ---------- -GCATTACAG GACAGCAGTG CAAGACGCAT GAAACTGAAC
     Cl39  ---------- ---------- ---------- ---------- ---------- ---------- ---------- ---------- ---------- -GCATTACAG GACAGCAGTG CAAGACGCAT GAAACTGAAC
Consensus  .......... .......... .......... .......... .......... .......... .......... .......... .......... .GCATTACAG GACAGcagTG CAAGACGCAT GAAACTGAAC
```

```
          8061                                                                                                                   8190
     gDNA ATGCTCAAAT ATGAATCTGT ACAATACCTC TCTTGACCTC GTTATTGTTC TTTCTTTGCT CAAGTTTAGT GACATCAGTG TTTTCTTACC TCAAGGCATG TAGGTTTTGA TTAATTACAG TTGTTCAGAT
     Cl37 ATGCTCAAAT ATGAATCT-- ---------- ---------- ---------- ---------- ---------- ---------- ---------- ---------- ---------- ---------- ----------
     Cl56 ATGCTCAAAT ATGAATCT-- ---------- ---------- ---------- ---------- ---------- ---------- ---------- ---------- ---------- ---------- ----------
     Cl42 ATGCTCAAAT ATGAATCT-- ---------- ---------- ---------- ---------- ---------- ---------- ---------- ---------- ---------- ---------- ----------
     Cl38 ATGCTCAAAT ATGAATCT-- ---------- ---------- ---------- ---------- ---------- ---------- ---------- ---------- ---------- ---------- ----------
     Cl39 ATGCTCAAAT ATGAATCT-- ---------- ---------- ---------- ---------- ---------- ---------- ---------- ---------- ---------- ---------- ----------
Consensus ATGCTCAAAT ATGAATCT.. .......... .......... .......... .......... .......... .......... .......... .......... .......... .......... ..........

          8191                                                                                                                   8320
     gDNA GTTGTGTAAG GCTGTTGGCT GCTTATTCTG TCATTTTTAT TCTTAATTGT AGCTCAAGAG GGAATTACTG CTTCTATCTG TGGGTATTGG ACTGGCTTGT AGTGGATATT GCTTGGTTAT TTTTTCCGTA
     Cl37 ---------- ---------- ---------- ---------- ---------- --CTCAAGAG GGAATTACTG CTTCTATCTG TGGGTATTGG ACTGGCTTGT AGTGGATATT GCTTGGTTAT TTTTTCCGTA
     Cl56 ---------- ---------- ---------- ---------- ---------- --CTCAAGAG GGAATTACTG CTTCTATCTG TGGGTATTGG ACTGGCTTGT AGTGGATATT GCTTGGTTAT TTTTTCCGTA
     Cl42 ---------- ---------- ---------- ---------- ---------- --CTCAAGAG GGAATTACTG CTTCTATCTG TGGGTATTGG ACTGGCTTGT AGTGGATATT GCTTGGTTAT TTTTTCTGTA
     Cl38 ---------- ---------- ---------- ---------- ---------- --CTCAAGAG GGAATTACTG CTTCTATCTG TGGGTATTGG ACTGGCCTGT AGTGGATATT GCTTGGTTAT TTTTTCCGTA
     Cl39 ---------- ---------- ---------- ---------- ---------- --CTCAAGAG GGAATTACTG CTTCTATCTG TGGGTATTGG ACTGGCTTGT AGTGGATATT GCTTGGTTAT TTTTTCCGTA
Consensus .......... .......... .......... .......... .......... ..CTCAAGAG GGAATTACTG CTTCTATCTG TGGGTATTGG ACTGGCtTGT AGTGGATATT GCTTGGTTAT TTTTTCcGTA

          8321                                                                                                                   8450
     gDNA CAGGTAAACT TTACATTTGC ATGCTGTGTC TCAAAAAATA ATAAAAAAGT CTTATCATTT TGCTATTATA ATTCAGAACC AGATCATTTT TGTGCTTCTT ACCACTCAAT TATGTTCTCT TTCTTCATGA
     Cl37 CAG------- ---------- ---------- ---------- ---------- ---------- ---------- ---------- ---------- ---------- ---------- ---------- ----------
     Cl56 CAG------- ---------- ---------- ---------- ---------- ---------- ---------- ---------- ---------- ---------- ---------- ---------- ----------
     Cl42 CAG------- ---------- ---------- ---------- ---------- ---------- ---------- ---------- ---------- ---------- ---------- ---------- ----------
     Cl38 CAG------- ---------- ---------- ---------- ---------- ---------- ---------- ---------- ---------- ---------- ---------- ---------- ----------
     Cl39 CAG------- ---------- ---------- ---------- ---------- ---------- ---------- ---------- ---------- ---------- ---------- ---------- ----------
Consensus CAG....... .......... .......... .......... .......... .......... .......... .......... .......... .......... .......... .......... ..........

          8451                                                                                                                   8580
     gDNA TAAGTCTAAC TTTGGATGGA TGCCAATGTC TTACCATAAT GCTTTAACTT CAAACACTAC CCCATGGTGT AAGTTTCAAA TTATTTCCTT CACTGATGTG TGCCATTCTC CTAAATGTGT AGGCTGCTAT
     Cl37 ---------- ---------- ---------- ---------- ---------- ---------- ---------- ---------- ---------- ---------- ---------- ---------- --GCTGCTAT
     Cl56 ---------- ---------- ---------- ---------- ---------- ---------- ---------- ---------- ---------- ---------- ---------- ---------- --GCTGCTAT
     Cl42 ---------- ---------- ---------- ---------- ---------- ---------- ---------- ---------- ---------- ---------- ---------- ---------- --GCTGCTAT
     Cl38 ---------- ---------- ---------- ---------- ---------- ---------- ---------- ---------- ---------- ---------- ---------- ---------- --GCTGCTAT
     Cl39 ---------- ---------- ---------- ---------- ---------- ---------- ---------- ---------- ---------- ---------- ---------- ---------- --GCTGCTAT
Consensus .......... .......... .......... .......... .......... .......... .......... .......... .......... .......... .......... .......... ..GCTGCTAT

          8581                                                                                                                   8710
     gDNA AAGTTATGCG ATTGGAGTCC TTTTCAGGTT TGTTATTGCA TTGTATTATA TTGATACTGT CATATTGAAT TATTTGTTAA ATGAGCTTAA GGGCCAGTAG TAAGACAGTT AGAGCCTAAA TTGATATTGT
     Cl37 AAGTTATGCG ATTGGAGTCC TTTTCAG--- ---------- ---------- ---------- ---------- ---------- ---------- ---------- ---------- ---------- ----------
     Cl56 AAGTTATGCG ATTGGAGTCC TTTTCAG--- ---------- ---------- ---------- ---------- ---------- ---------- ---------- ---------- ---------- ----------
     Cl42 AAGTTATGCG ATTGGAGTCC TTTTCAG--- ---------- ---------- ---------- ---------- ---------- ---------- ---------- ---------- ---------- ----------
     Cl38 AAGTTATGCG ATTGGAGTCC TTTTCAG--- ---------- ---------- ---------- ---------- ---------- ---------- ---------- ---------- ---------- ----------
     Cl39 AAGTTATGCG ATTGGAGTCC TTTTCAG--- ---------- ---------- ---------- ---------- ---------- ---------- ---------- ---------- ---------- ----------
Consensus AAGTTATGCG ATTGGAGTCC TTTTCAG... .......... .......... .......... .......... .......... .......... .......... .......... .......... ..........

          8711                                                                                                                   8840
     gDNA TCATCATTGT TTTTTTTGTG TGTGAGAGAA AAGAAAAGAT TAAACTGTTC TTAAAATTTG AGATTCAGAC AATAGGATAA AAAATGTAAC TATCCACACA TTCTAATTTA ATCTTTTTTT TTTAATCTTA
Consensus .......... .......... .......... .......... .......... .......... .......... .......... .......... .......... .......... .......... ..........

          8841                                                                                                                   8970
     gDNA AGAATCGAAG ATAGATATCA AAGAGTTTAT AACAACTCAC GCATTCTAAT TTAATTTAAC AATAATAGGT GATGAATTAA TTGAATAGGT TGAAACCTGG CAAGACATAT TTGCCGGTGG CATCAATGAC
     Cl37 ---------- ---------- ---------- ---------- ---------- ---------- ---------- ---------- --------GT TGAAACCTGG CAAGACATAT TTGCCGGTGG CATCAATGAC
     Cl56 ---------- ---------- ---------- ---------- ---------- ---------- ---------- ---------- --------GT TGAAACCTGG CAAGACATAT TTGCCGGTGG CATCAATGAC
     Cl42 ---------- ---------- ---------- ---------- ---------- ---------- ---------- ---------- --------GT TGAAACCTGG CAAGACATAT TTGCCGGTGG CATCAATGAC
     Cl38 ---------- ---------- ---------- ---------- ---------- ---------- ---------- ---------- ---------- ---------- ---------- ---------- ----------
     Cl39 ---------- ---------- ---------- ---------- ---------- ---------- ---------- ---------- ---------- ---------- ---------- ---------- ----------
Consensus .......... .......... .......... .......... .......... .......... .......... .......... ........gt tgaaacctgg caagacatat ttgccggtgg catcaatgac

          8971                                                                                                                   9100
     gDNA AGTGACATCA TCTCCAACCT GGGAATGGAA ATGAATCATA AGCAGAAAGT AACAAAAAGC GCAAGGTGTT AGAAAGCAAA TTAAGCACTG CTACTGCTAG GGGGTTTCAA TTCATCAAAT ACCCTGAAGT
     Cl37 AGTGACATCA TCTCCAACCT GGGAATGGAA ATGAATCATA AGCAGAAAGT AACAAAAAGC GCAAGGTGTT AGAAAGCAAA TTAAGCACTG CTACTGCTAG GGGGTTTCAA TTCATCAAAT ACCCTGAAGT
     Cl56 AGTGACATCA TCTCCAACCT GGGAATGGAA ATGAATCATA AGCAGAAAGT AACAAAAAGC GCAAGGTGTT AGAAAGCAAA TTAAGCACTG CTACTGCTAG GGGGTTTCAA TTCATCAAAT ACCCTGAAGT
     Cl42 AGTGACATCA TCTCCAACCT GGGAATGGAA ATGAATCATA AGCAGAAAGT AACAAAAAGC GCAAGGTGTT AGAAAGCAAA TTAAGCACTG CTACTGCTAG GGGGTTTCAA TTCATCAAAT ACCCTGAAGT
     Cl38 ---------- ---------- ---------- ---------- ---------- ---------- ---------- ---------- ---------- ---------- ---------- ---------- ----------
     Cl39 ---------- ---------- ---------- ---------- ---------- ---------- ---------- ---------- ---------- ---------- ---------- ---------- ----------
Consensus agtgacatca tctccaacct gggaatggaa atgaatcata agcagaaagt aacaaaaagc gcaaggtgtt agaaagcaaa ttaagcactg ctactgctag ggggtttcaa ttcatcaaat accctgaagt
```

```
          9101                                                                                                                           9230
     gDNA TAGGATTAAC TGCAACAATG ATGCCATCTT CCACGTAAAC ATCAGCCATC TGTTGGTGGT GACCATTCAC AACCGTGCCT CCCTTGATCA ACAACTTGGA TGATGGAATT TCAGTCCCAG CATCACAAAA
     Cl37 TAGGATTAAC TGCAACAATG ATGCCATCTT CCACGTAAAC ATCAGCCATC TGTTGGTGGT GACCATTCAC AACCGTGCCT CCCTTGATCA ACAACTTGGA TGATGGAATT TCAGTCCCAG CATCACAAAA
     Cl56 TAGGATTAAC TGCAACAATG ATGCCATCTT CCACGTAAAC ATCAGCCATC TGTTGGTGGT GACCATTCAC AACCGTGCCT CCCTTGATCA ACAACTTGGA TGATGGAATT TCAGTCCCAG CATCACAAAA
     Cl42 TAGGATTAAC TGCAACAATG ATGCCATCTT CCACGTAAAC ATCAGCCATC TGTTGGTGGT GACCATTCAC AACCGTGCCT CCCTTGATCA ACAACTTGGA TGATGGAATT TCAGTCCCAG CATCACAAAA
     Cl38 ---------- ---------- ---------- ---------- ---------- ---------- ---------- ---------- ---------- ---------- ---------- ---------- ----------
     Cl39 ---------- ---------- ---------- ---------- ---------- ---------- ---------- ---------- ---------- ---------- ---------- ---------- ----------
Consensus taggattaac tgcaacaatg atgccatctt ccacgtaaac atcagccatc tgttggtggt gaccattcac aaccgtgcct cccttgatca acaacttgga tgatggaatt tcagtcccag catcacaaaa

          9231                                                                                                                           9360
     gDNA CTGACTCACT CGAAAAGGAC AAACATGAAT TAAACATAGA ACTAGTGGAA ATGAAAATCT CAGCTCAGTA CTAATGATCA TAGCTACTTA AAACAGTTAA GAATCGAAGC TCTAACTCTT GGAAAACGCA
     Cl37 CTGACTCACT CGAAAAGGAC AAACATGAAT TAAACATAGA ACTAGTGGAA ATGAAAATCT CAGCTCAGTA CTAATGATCA TAGCTACTTA AAACAGTTAA GAATCGAAGC TCTAACTCTT GGAAAACGCA
     Cl56 CTGACTCACT CGAAAAGGAC AAACATGAAT TAAACATAGA ACTAGTGGAA ATGAAAATCT CAGCTCAGTA CTAATGATCA TAGCTACTTA AAACAGTTAA GAATCGAAGC TCTAACTCTT GGAAAACGCA
     Cl42 CTAACTCACT CGAAAAGGAC AAACATGAAT TAAACATAGA ACTAGTGGAA ATGAAAATCT CAGCTCAGTA CTAATGATCA TAGCTACTTA AAACAGTTAA GAATCGAAGC TCTAACTCTT GGAAAACGCA
     Cl38 ---------- ---------- ---------- ---------- ---------- ---------- ---------- ---------- ---------- ---------- ---------- ---------- ----------
     Cl39 ---------- ---------- ---------- ---------- ---------- ---------- ---------- ---------- ---------- ---------- ---------- ---------- ----------
Consensus ctgactcact cgaaaaggac aaacatgaat taaacataga actagtggaa atgaaaatct cagctcagta ctaatgatca tagctactta aaacagttaa gaatcgaagc tctaactctt ggaaaacgca

          9361                                                                                                                           9490
     gDNA GAACGGGGTA AGTAAACTAG GTGAGCATAG GTCAACACAT AAGAGAATTA TTCCCTGCAT TCAGTTTTTA TAAAACTTGC AGAAGTATTA ATGCAACTCC CCCTCCCTTT GGTAGTCACT AAAAGAACAA
     Cl37 GAACGGG--- ---------- ---------- ---------- ---------- ---------- ---------- ---------- ---------- ---------- ---------- -----TCACT AAAAGAACAA
     Cl56 GAACGGG--- ---------- ---------- ---------- ---------- ---------- ---------- ---------- ---------- ---------- ---------- -----TCACT AAAAGAACAA
     Cl42 GAACGGG--- ---------- ---------- ---------- ---------- ---------- ---------- ---------- ---------- ---------- ---------- ---------- ----------
     Cl38 ---------- ---------- ---------- ---------- ---------- ---------- ---------- ---------- ---------- ---------- ---------- ---------- ----------
     Cl39 ---------- ---------- ---------- ---------- ---------- ---------- ---------- ---------- ---------- ---------- ---------- ---------- ----------
Consensus gaacggg... .......... .......... .......... .......... .......... .......... .......... .......... .......... .......... .......... ..........

          9491                                                                                                                           9620
     gDNA GCGCACTTGA ACTTCTATGG TTGATGTAAG GGTACGTGTC TTTGATTTTC CCCCATGCTT GTTTGCAAGT GTAAACATGC TCAATTTCAC TCTTTTCAAT TCTTCCTACA ATTGCTTATT CATTTGCTTA
     Cl37 GCGCACTTGA ACTTCTATGG TTGATGTAAG G--------- ---------- ---------- ---------- ---------- ---------- ---------- ---------- ---------- ----------
     Cl56 GCGCACTTGA ACTTCTATGG TTGATGTAAG G--------- ---------- ---------- ---------- ---------- ---------- ---------- ---------- ---------- ----------
     Cl42 ---------- ---------- ---------- ---------- ---------- ---------- ---------- ---------- ---------- ---------- ---------- ---------- ----------
     Cl38 ---------- ---------- ---------- ---------- ---------- ---------- ---------- ---------- ---------- ---------- ---------- ---------- ----------
     Cl39 ---------- ---------- ---------- ---------- ---------- ---------- ---------- ---------- ---------- ---------- ---------- ---------- ----------
Consensus .......... .......... .......... .......... .......... .......... .......... .......... .......... .......... .......... .......... ..........

          9621                                                                                                                           9750
     gDNA CTGTTTCAAA ATTTAAAATA AATACTGTAC AGTCATAAAT AATAGTATAT ATGGCCTAGT AGTATATTTT GATTTTGTAG TATCTCTAAT TTACATGGAA ACCGCCTTTT TTAAAAATTG TCTTTGCAAA
Consensus .......... .......... .......... .......... .......... .......... .......... .......... .......... .......... .......... .......... ..........

          9751                                                                                                                           9880
     gDNA AATTCCCTGC TAGAACATTT ATCCAGTGGT ATTCTTGAAA AAAAATATAT GTAGATTTAT TGTTTACATT ATGTATTCTG TCTGAAGCTT GTAAAGAGCT TTATGTTCGT TTATGCCTTT CCCATTTTTT
Consensus .......... .......... .......... .......... .......... .......... .......... .......... .......... .......... .......... .......... ..........

          9881                                                                                                                          10010
     gDNA TTCTCTGGAA GGGGTGAAAT ATAGGATATT CTACTTTTTC AGTTTTTAGG TTCTAATAAG GCATAATTAA GTGGTAGACT CAGCTTTAAA ATTTTGTTCT TTCCTTTATA TGGAGAATTA TGTTTCAGAT
Consensus .......... .......... .......... .......... .......... .......... .......... .......... .......... .......... .......... .......... ..........

          10011                                                                                                                         10140
     gDNA TAATGTGACT TGTAATAAAA CCTTTTGAGC AAAACTGACT TGTTTTTATA AATTTCAGAT GGATGTGCGG ACTAATTCTC TCGTCAGGCC ATCAGGGACA ATTGACAAAG ATAAGGAAAA GCTACGGATT
     Cl37 ---------- ---------- ---------- ---------- ---------- --------AT GGATGTGCGG ACTAATTCTC TCGTCAGGCC ATCAGGGACA ATTGACAAAG ATAAGGAAAA GCTACGGATT
     Cl56 ---------- ---------- ---------- ---------- ---------- --------AT GGATGTGCGG ACTAATTCTC TCGTCAGGCC ATCAGGGACA ATTGACAAAG ATAAGGAAAA GCTACGGATT
     Cl42 ---------- ---------- ---------- ---------- ---------- --------GT GGATGTGCGG ACTAATTCTC TCGTCAGGCC ATCAGGGACA ATTGACAAAG ATAAGGAAAA GCTACGGATT
     Cl38 ---------- ---------- ---------- ---------- ---------- --------AT GGATGTGCGG ACTAATTCTC TCGTCAGGCC ATCAGGGACA ATTGACAAAG ATAAGGAAAA GCTACGGATT
     Cl39 ---------- ---------- ---------- ---------- ---------- --------AT GGATGTGCGG ACTAATTCTC TCGTCAGGCC ATCAGGGACA ATTGACAAAG ATAAGGAAAA GCTACGGATT
Consensus .......... .......... .......... .......... .......... ........aT GGATGTGCGG ACTAATTCTC TCGTCAGGCC ATCAGGGACA ATTGACAAAG ATAAGGAAAA GCTACGGATT

          10141                                                                                                                         10270
     gDNA GCCAACAATG GTGTCGTTCA GAGTGAAGAA CAAACCTTAC CTATTGGAGG TGATGGTTGG GAAAAGTCAA AAATGAAGAA GAAGCGTTCC TGTATCAAAC TAGATGTTTC TCCCAGTACA ACATTGACTA
     Cl37 GCCAACAATG GTGTCGTTCA GAGTGAAGAA CAAACCtTAC CTATTGGAGG TGATGGTTGG GAAAAGTCAA AAATGAAGAA GAAGCGTTCC TGTATCAAAC TAGATGTTTC TCCCAGTACA ACATTGACTA
     Cl56 GCCAACAATG GTGTCGTTCA GAGTGAAGAA CAAACCTTAC CTATTGGAGG TGATGGTTGG GAAAAGTCAA AAATGAAGAA GAAGCGTTCC TGTATCAAAC TAGATGTTTC TCCCAGTACA ACATTGACTA
     Cl42 GCCAACAATG GTGTCGTTCA GAGTGAAGAA CAAACCTTAC CTATTGGAGG TGATGGTTGG GAAAAGTCAA AAATGAAGAA GAAGCGTTCC TGTATCAAAC TAGATGTTTC TCCCAGTACA ACATTGACTA
     Cl38 GCCAACAATG GTGTCGTTCA GAGTGAAGAA CAAACCTTAC CTATTGGAGG TGATGGTTGG GAAAAGTCAA AAATGAAGAA GAAGCGTTCC TGTATCAAAC TAGATGTTTC TCCCAGTACA ACATTGACTA
     Cl39 GCCAACAATG GTGTCGTTCA GAGTGAAGAA CAAACCTTAC CTATTGGAGG TGATGGTTGG GAAAAGTCAA AAATGAAGAA GAAGCGTTCC TGTATCAAAC TAGATGTTTC TCCCAGTACA ACATTGACTA
Consensus GCCAACAATG GTGTCGTTCA GAGTGAAGAA CAAACCtTAC CTATTGGAGG TGATGGTTGG GAAAAGTCAA AAATGAAGAA GAAGCGTTCC TGTATCAAAC TAGATGTTTC TCCCAGTACA ACATTGACTA
```

```
           10271                                                                                                                      10400
     gDNA  AACCTGTTAA CACCTTCCAA GAAACTAAAC AGGGAATGCA ACAAAGACTT GCTACCGATT CGCGATTCTT TCAGGTATGA ATGTGTTATT TAATTATTTT TTTTTTGTTC TCATATAATA TTTCTTATTT
     Cl37  AACCTGTTAA CACCTTCCAA GAAACTAAAC AGGGAATGCA ACAAAGACTT GCTACCGATT CGCGATTCTT TCAG------ ---------- ---------- ---------- ---------- ----------
     Cl56  AACCTGTTAA CACCTTCCAA GAAACTAAAC AGGGAATGCA ACAAAGACTT GCTACCGATT CGCGATTCTT TCAG------ ---------- ---------- ---------- ---------- ----------
     Cl42  AACCTGTTAA CACCTTCCAA GAAGCTAAAC AGGGAATGCA ACAAAGACTT GCTACCGATT CGCGATTCTT TCAG------ ---------- ---------- ---------- ---------- ----------
     Cl38  AACCTGTTAA CACCTTCCAA GAAACTAAAC AGGGAATGCA ACAAAGACTT GCTACCGATT CGCGATTCTT TCAG------ ---------- ---------- ---------- ---------- ----------
     Cl39  AACCTGTTAA CACCTTCCAA GAAACTAAAC AGGGAATGCA ACAAAGACTT GCTACCGATT CGCGATTCTT TCAG------ ---------- ---------- ---------- ---------- ----------
Consensus  AACCTGTTAA CACCTTCCAA GAAaCTAAAC AGGGAATGCA ACAAAGACTT GCTACCGATT CGCGATTCTT TCAG...... .......... .......... .......... .......... ..........

           10401                                                                                                                      10530
     gDNA  TAGGCTAAAA TAATATTTTA TGGGAAGATT AGAAAGTAGG CACATTTTTT GTGGGAGGAT TTAGGATAAG AAAATCCTCC CTTAAAATGA GGAAAAAAGA GTCAAAATGT CCAAAAAAAA ATGTTAGGCT
Consensus  .......... .......... .......... .......... .......... .......... .......... .......... .......... .......... .......... .......... ..........

           10531                                                                                                                      10660
     gDNA  TTTCAATCTT TATGCACATC AACAAAAGAA ATTCCTTTGA AATAACCACG AGCATATCCC TAGAGAAAAT CCATACATGA ATTTTGGGGC AAAGAGACAT TCTTGAAAAT GCAATCATTC CAATTCCTCT
Consensus  .......... .......... .......... .......... .......... .......... .......... .......... .......... .......... .......... .......... ..........

           10661                                                                                                                      10790
     gDNA  CTAATAAACT GCCTGATAAC TGTATAAATT GCTTAGTTCC ATAGAATTGT AGCCTCCTGT TTGCTGCCAA ATGTTACAAT TGGAGAATGA AAATAAATTA TAGGCAGTAA CTGTTTGGAG TTCTCTCTAG
Consensus  .......... .......... .......... .......... .......... .......... .......... .......... .......... .......... .......... .......... ..........

           10791                                                                                                                      10920
     gDNA  AGTGTTAATT GTGAAAGGAT TTTCTTCAAA GAAAAACAAT ACAGTTGTGA AGGATTTTGC TGATATTAAG GGTCATGAAT TTAAAATCTT TGATGGATGA TATCATGTAA CAATTTATTT ATTTATTTGC
Consensus  .......... .......... .......... .......... .......... .......... .......... .......... .......... .......... .......... .......... ..........

           10921                                                                                                                      11050
     gDNA  TCTATCCAAT TCTGATTTAT TTTCTTGCTT AAACCTAGCC GATAAATATA ATAATTTCAT GTATTTTTTT TAGGTCAGTA GTTTGTAATG GAACTATTGG AGTTGGAAAA TCAGATGGTA TCTCTCAACA
     Cl37  ---------- ---------- ---------- ---------- ---------- ---------- ---------- ---GTCAGTA GTTTGTAATG GAACTATTGG AGTTGGAAAA TCAGATGGTA TCTCTCAACA
     Cl56  ---------- ---------- ---------- ---------- ---------- ---------- ---------- ---GTCAGTA GTTTGTAATG GAACTATTGG AGTTGGAAAA TCAGATGGTA TCTCTCAACA
     Cl42  ---------- ---------- ---------- ---------- ---------- ---------- ---------- ---GTCAGTA GTTTGTAATG GAACTATTGG AGTTGGAAAA TCAGATGGTA TCTCTCAACA
     Cl38  ---------- ---------- ---------- ---------- ---------- ---------- ---------- ---GTCAGTA GTTTGTAATG GAACTATTGG AGTTGGAAAA TCAGATGGTA TCTCTCAACA
     Cl39  ---------- ---------- ---------- ---------- ---------- ---------- ---------- ---GTCAGTA GTTTGTAATG GAACTATTGG AGTTGGAAAA TCAGATGGTA TCTCTCAACA
Consensus  .......... .......... .......... .......... .......... .......... .......... ...GTCAGTA GTTTGTAATG GAACTATTGG AGTTGGAAAA TCAGATGGTA TCTCTCAACA

           11051                                                                                                                      11180
     gDNA  AACTGGGTTG GGCATACGAG CTTCTACCCC TAGAAACAAC CAAGATAATA ATTCCCTTGT CAATGATAGG AGGGGTCGTC CTGTTAGTTC AGACAAGGAA AGGGTGAACT TCAGAGTTGT AAACAAGTAA
     Cl37  AACTGGGTTG GGCATACGAG CTTCTACCCC TAGAAACAAC CAAGATAATA ATTCCCTTGT CAATGATAGG AGGGGTCGTC CTGTTAGTTC AGACAAGGAA AGGGTGAACT TCAGAGTTGT AAACAA----
     Cl56  AACTGGGTTG GGCATACGAG CTTCTACCCC TAGAAACAAC CAAGATAATA ATTCCCTTGT CAATGATAGG AGGGGTCGTC CTGTTAGTTC AGACAAGGAA AGGGTGAACT TCAGAGTTGT AAACAA----
     Cl42  AACTGGGTTG GGCATACGAG CTTCTACCCC TAGAAACAAC CAAGATAATA ATTCCCTTGT CAATGATAGG AGGGGTCGTC CTGTTAGTTC AGACAAGGAA AGGGTGAACT TCAGAGTTGT AAACAA----
     Cl38  AACTGGGTTG GGCATACGAG CTTCTACCCC TAGAAACAAC CAAGATAATA ATTCCCTTGA CAATGATAGG AGGGGTCGTC CTGTTAGTTC AGACAAGGAA AGGGTGAACT TCAGAGTTGT AAACAA----
     Cl39  AACTGGGTTG GGCATACGAG CTTCTACCCC TAGAAACAAC CAAGATAATA ATTCCCTTGT CAATGATAGG AGGGGTCGTC CTGTTAGTTC AGACAAGGAA AGGGTGAACT TCAGAGTTGT AGACAA----
Consensus  AACTGGGTTG GGCATACGAG CTTCTACCCC TAGAAACAAC CAAGATAATA ATTCCCTTGt CAATGATAGG AGGGGTCGTC CTGTTAGTTC AGACAAGGAA AGGGTGAACT TCAGAGTTGT AaACAA....

           11181                                                                                                                      11310
     gDNA  ATACAAGCTG TGGAACCCCT TTTCTTTATA TGATTGTGCG TTTGGGAAAA AGAATTGTCC TTCACTAAAA TGTTTTTGCA TGTTGCTTTC TCATTTTCTC CTTATTTTCG TCTTGTTTTG GTAGAATTGT
Consensus  .......... .......... .......... .......... .......... .......... .......... .......... .......... .......... .......... .......... ..........

           11311                                                                                                                      11440
     gDNA  TCAATTGTTT GCTTTCTGTT TGCTATGGAG CCTTGCGATT ATAGTTCATA TATCTCTTCA TCTTTGTGAT ATTTTTTCTG GTATTGATGA TTCATTCAGT ATCTGTGTTA TGTTCTATGA CCAGGGCAAC
Consensus  .......... .......... .......... .......... .......... .......... .......... .......... .......... .......... .......... .......... ..........

           11441                                                                                                                      11570
     gDNA  TGCACGTGAT GAATTTAATT CATCTAGCCC TACCTCAAGT GCTAAAATAA ACACTGCTAT CTGTGTTATG ATATTTTTTA TTAGTTCTAT TGCATGCAAT CAAGTTAATG GTATACGTCA CTATTATTAC
Consensus  .......... .......... .......... .......... .......... .......... .......... .......... .......... .......... .......... .......... ..........

           11571                                                                                                                      11700
     gDNA  TGAGAAGTGG TTAAATGTCA AGTAGATCAA GCATGCAATA TATAATTTTG GTTTAGATAT TTATTTGGTG TTATAGATAT GATCTAGAAG ATAAAATACA GCACAGCCAT TAACTGTTGT AGCTTAATAT
Consensus  .......... .......... .......... .......... .......... .......... .......... .......... .......... .......... .......... .......... ..........

           11701                                                                                                                      11830
     gDNA  GACATTGACA CTGATCGATT TCTGTATGTA TCCAATGTCA TTTGAAAACT GTATTTGCTA GCTTCCATTA CCTCTTGTTT AGGTTAAACT AAACCCAGAA TCTAAATTGA AAATGCTCAC TGTAATTGGC
Consensus  .......... .......... .......... .......... .......... .......... .......... .......... .......... .......... .......... .......... ..........

           11831                                                                                                                      11960
     gDNA  TTGTATCTGG GAATATGGGT GGGGTGGGGT GTGGAATAAA CTTCTCCATA AGCACTTCTT GGGAAAAAAT GAGAAGGTAA AATGCATTGA GCTTCTCCAT AAGCTAAAAA ATCAGCTTAT ACACCTTATC
Consensus  .......... .......... .......... .......... .......... .......... .......... .......... .......... .......... .......... .......... ..........
```

```
          11961                                                                                                                                 12090
     gDNA TTTTGGAGAA GATAAACGAA AAGAGCTTTT ATAAAAATTA AGTGCATAAG TTGATTTTAG CTTATGTGCA AAATTCAATT CATTATATCT TCTTATTTTG GTAGTGAAGT TTATCAAAAC GAGGTCAATG
Consensus .......... .......... .......... .......... .......... .......... .......... .......... .......... .......... .......... .......... ..........

          12091                                                                                                                                 12220
     gDNA AATACAACTT TTTTGGCTTT AGTAATTTCG AGCAACAAAT AGCATTGATG TGACCTTGAT TTGACTATTT CAGTGTTAGA ACTTTTTTGA AAACTACAAA TGTTTATTAT GTTCTGCAAA TTCTATATTA
Consensus .......... .......... .......... .......... .......... .......... .......... .......... .......... .......... .......... .......... ..........

          12221                                                                                                                                 12350
     gDNA TCTTTCTATT AGAAAAGTCT TTGTTATTTA GAGATCTTGC ATGCTAATCT TTATTATTAA ATATTAATTT CTATTTACTA AGTTTTGAGC CTAGATATAT GTTTATTTAG AGATCTGAAG CTATATACTA
Consensus .......... .......... .......... .......... .......... .......... .......... .......... .......... .......... .......... .......... ..........

          12351                                                                                                                                 12480
     gDNA TTGCCTGCAT TTACTTATGT TTATTATTGG CATAACCTGT TGGTCTAAAT TTTAGTTTCT AGTGATGCAT TACTGTTTTA TGCAGGTCTT TTCTGACTTG GAGCGTTCAG TGTGTAGAAT GGTCCATCGA
     Cl37 ---------- ---------- ---------- ---------- ---------- ---------- ---------- ---------- -----GTCTT TTCTGACTTG GAGCGTTCAG TGTGTAGAAT GGTCCATCGA
     Cl56 ---------- ---------- ---------- ---------- ---------- ---------- ---------- ---------- -----GTCTT TTCTGACTTG GAGCGTTCAG TGTGTAGAAT GGTCCATCGA
     Cl42 ---------- ---------- ---------- ---------- ---------- ---------- ---------- ---------- -----GTCTT TTCTGACTTG GAGCGTTCAG TGTGTAGAAT GGTCCATCGA
     Cl38 ---------- ---------- ---------- ---------- ---------- ---------- ---------- ---------- -----GTCTT TTCTGACTTG GAGCGTTCAG TGTGTAGAAT GGTCCATCGA
     Cl39 ---------- ---------- ---------- ---------- ---------- ---------- ---------- ---------- -----GTCTT TTCTGACTTG GAGCGTTCAG TGTGTAGAAT GGTCCATCGA
Consensus .......... .......... .......... .......... .......... .......... .......... .......... .....GTCTT TTCTGACTTG GAGCGTTCAG TGTGTAGAAT GGTCCATCGA

          12481                                                                                                                                 12610
     gDNA CAGGTTTGAT TCTGATAAAT TGCTTTTAAT GTTTTCTACT GTTGGTTTGA GAGATTATAT TTACATTTTA TTTTAGTAAC TTGAGTATCG TATTCCGATT ATGGCAGAAC TTGATCAAGT TCAAGTGTAT
     Cl37 CAG------- ---------- ---------- ---------- ---------- ---------- ---------- ---------- ---------- ---------- ---------- ---------- ----------
     Cl56 CAG------- ---------- ---------- ---------- ---------- ---------- ---------- ---------- ---------- ---------- ---------- ---------- ----------
     Cl42 CAG------- ---------- ---------- ---------- ---------- ---------- ---------- ---------- ---------- ---------- ---------- ---------- ----------
     Cl38 CAG------- ---------- ---------- ---------- ---------- ---------- ---------- ---------- ---------- ---------- ---------- ---------- ----------
     Cl39 CAG------- ---------- ---------- ---------- ---------- ---------- ---------- ---------- ---------- ---------- ---------- ---------- ----------
Consensus CAG....... .......... .......... .......... .......... .......... .......... .......... .......... .......... .......... .......... ..........

          12611                                                                                                                                 12740
     gDNA GATGAATTCC TTTTTGTTTA GCATTGGGAT CATGGAACAA GTTTCCTTGA TTTCATTTAA AATATTTTGA AGATTTAAAT CAGTTCATTT GATTCTGTAA TATTAAGATT TGTAACTTAA TTACAATTTT
Consensus .......... .......... .......... .......... .......... .......... .......... .......... .......... .......... .......... .......... ..........

          12741                                                                                                                                 12870
     gDNA GATTGTACAT ACCTTTCTCT GTTCCCATGG AGCTTATTTT TTAATAAATA GACCTTTTGG ATTTGTATTT AGTTTTTGTC TTACTACGGA TGCTTCTTTT CTTAACTTGG GGGATTCGTT TTTTAATTTT
Consensus .......... .......... .......... .......... .......... .......... .......... .......... .......... .......... .......... .......... ..........

          12871                                                                                                                                 13000
     gDNA TTTGTAGGTT GCCACAATTG CTTGGTTGGA AGCTGATTCT GTTTGTGGCA GCCACTCTAT ATGACTTCAC ATTTTCTTAT TACCTTTTAA TCTATAATAT CATGTATTTA TGCAAATGAA CTGCATATAC
     Cl37 -------GTT GCCACAATTG CTTGGTTGGA AGCTGATTCT GTTTGTGGCA GCCACTCTAT ATGACTTCAC A
     Cl56 -------GTT GCCACAATTG CTTGGTTGGA AGCTGATTCT GTTTGTGGCA GCCACTCTAT ATGACTTCAC A
     Cl42 -------GTT GCCACAATTG CTTGGTTGGA AGCTGATTCT GTTTGTGGCA GCCACTCTAT ATGACTTCAC A
     Cl38 -------GTT GCCACAATTG CTTGGTTGGA AGCTGATTCT GTTTGTGGCA GCCACTCTAT ATGACTTCAC A
     Cl39 -------GTT GCCACAATTG CTTGGTTGGA AGCTGATTCT GTTTGTGGCA GCCACTCTAT ATGACTTCAC A
Consensus .......GTT GCCACAATTG CTTGGTTGGA AGCTGATTCT GTTTGTGGCA GCCACTCTAT ATGACTTCAC A......... .......... .......... .......... .......... ..........

          13001                                                                                                                                 13130
     gDNA CTGTGTAATG AACTGCTTGC AAATGAAGTG TGAGAGGTGA ATGCTTTTAT GAAAATAGAT TGATTGATGA ATTTCTGTTA CCATTCAGTG AAATCTGTCA AAGTATCATG TAGTTATGCT CACAATTTTT
Consensus .......... .......... .......... .......... .......... .......... .......... .......... .......... .......... .......... .......... ..........

          13131                                                                                                                                 13260
     gDNA TTAAATGTAT TTTGATTTTT AAACAATTTA TGTAAGTAAT CTTTCCTTGC TGTTGTTGAG AAGAATAAAA AATTGTTTGT TCTAGCATTT TAGAACTAAG TGATCTTTAT TTTATTTTTA TCTGATAAAC
Consensus .......... .......... .......... .......... .......... .......... .......... .......... .......... .......... .......... .......... ..........

          13261                                                                                                                                 13390
     gDNA ACTCGGTGAT CCTTATATCA ACTTTTTATC AATGTCCTTT GTTTGTTCAG GGATTTATTG CATCCGCCCT TAATTATGGA CTCATTACAT GGTGACGAAG GTTTCAGCAT TGCTTTATGA AAGTTTTGTT
Consensus .......... .......... .......... .......... .......... .......... .......... .......... .......... .......... .......... .......... ..........

          13391                                                                                                                                 13520
     gDNA GTCAAAGTTT GTAATTTGTG GACAAGTTAC AACTGAAGTT AGCAATGTTA GATATTCCTA TGTAATTTTA AATTCTTGCA AAACTATATA CTATCATTTA ATTATGTAAA TTCTTTTATT ATGTGATTAA
Consensus .......... .......... .......... .......... .......... .......... .......... .......... .......... .......... .......... .......... ..........

          13521                                                                                                                                 13650
     gDNA ACAATTTTAT AATTCTTTAA AATGATGTAA ATTCTTTCAT TAATTATGTA AATTCTTTTA TTATGTAAAT TCTTTTGCTT CAAATCAATG TATAGAATAA TTGAAACACT ACCTAAAAAA CTAGACCTGC
Consensus .......... .......... .......... .......... .......... .......... .......... .......... .......... .......... .......... .......... ..........
```
