# Supplementary material for: A putative autonomous 20.5 kb-CACTA transposon insertion in an F3'H allele identifies a new CACTA transposon subfamily in Glycine max
Source: BMC Plant Biol. 2008 Dec 2;8:124. doi: 10.1186/1471-2229-8-124 (PMC2613891; doi:10.1186/1471-2229-8-124)
Supplement: Additional file 7 — Tgmt* Gene-1 precursor transcripts sequence. The sequence of a precursor transcript (7,554 bp) expressed by Gene-1 was composed with all 24 exon sequences present in the cDNA clones analyzed (Figure 8 and see Additional file 5: Alignment of cDNA sequences of clones 43–53 to Tgmt* genomic sequence). [file 1471-2229-8-124-S7.pdf]

Additional file 7: *Tgmt Gene-1 precursor transcripts sequence** (7,554 bp)

AACAGCACGCATAACTGAAGAGTACGAAAATGGGGTTGAAGGTTTCTTAAAA
TTTGCTAAAGATAATGCATCCGACAATGGTGGACTATACTTTTGTCCTTGTGT
TAAATGTTTGAATGGGCGACGACAATGTTTGGATGACATTAGAACACACCTT
ATCTGTGATGGTATCTGTCCTACTTATACAAAATGGATATGGCATGGTGAGTT
ACCAGAAATGTCATCAACCCCTCCAACTGCTCCAACTGATGAACAAGTCGGT
GATCAAATAGAAGACATGCTACGTGATCTTGGACAAGAGGGTTTTAGGCAAG
CAAATGCACCGTATTATGACACCTTACATAATGATTCAAAGATTCCATTGTTT
ATTGGATGCACTAAGTACACACGGTTATCAGGGGTGTTAGCTCTGGTCAATTT
GAAAGCAAGATTTGGGTGGAGTGACAAAAGTTTCAATGAATTACTGTTGTTA
TTGAAGAATATGCTTCCAGGAGATAACACGTTGCCAAAGACTCATTACGAGG
CAAAGAAGATATTATGTCCTGTTGGAATGGAATACCAAAAAATACATGCTTG
CCGTAATGATTGCATTTTGTATAGACATGAGTTTGCTGAATTGCGCAACTGCC
CTACATGTGGGGTGTCACGCTACAAAGTGGGTTCTGGCGCTTCCAGTGAAGC
TGGATCCACATACATTGATCGGCCAGCAAAAGTGTGTTGGTATCTTCCAGTAA
TACCAAGGTTTAAGCGATTGTTTGCTAATGCAGAAGATGCAAAAAACCTAAC
ATGGCATGTTGATGGTAGGACCAAAGATGGATTGCTCCGTCATCCTGCTGATT
CTCCTCAGTGGAAGAAAGTTGATCAGTTGTATCCAGTGTTTGCCGAAGATCCC
AGAAACCTAAGGGTTGGTCTCGCATCGGATGGAATGAATCCATTTCGAAGCT
TAAGTTGCAATCATAGTTCGTGGCCTGTTTTGTTGATCATTTACAACCTGCCTC
CTTGGTTGTGCATCAAGCGGAAGTACATAATGATGTCTATGATGATAGCCGG
TCCAAGACAACCAGGAAATGACATTGATGTGTATCTTGCTCCCTTGATTGAAG
ACCTAACAAAATTGTGGGTAGAAGGGGTTGATGTGTATGATGGGAATGCTCA
TGAGTCCTTCAGGTTGCGAGCTATGATTTTCTGCACCATTAATGACTTTCCAG
CATATGGGAATTTGAGTGGATATAGTGTGAAAGGCCACCTTGCTTGTCCCATT
TGTGAGAAAGACACAACTTACCTCCAATTAAAGCATGGCAAAAAAACTGTAT
ATACAAGACACCGTAGATTTTTACAACCTTTTCACCCATACAGGCGACTGAA
GAAAGCGTTTGATGGGACATCTGAGAATGACAGTGCATCAATTCCTTTGTCA
GGTGTTGAAGTTTTTGATCGTGTGAAGAACATTTGCAATATATATGGGAAGA
CACAAAAGAAAGATGGCGCTCCCAAAAACATTTGGAAGAAAAGGTCCATCTT
CTTTGATCTTCCATACTGGTGCAACTTAGATGTGAGACATTGTTTAGATGTGA
TGCATGTTGAAAAAAATGTCTGTGACAGTTTGGTTGGCACACTGCTTAACATT
AAAGGGAAGACAAAAGATGGTTTGAAATGTCGTCAAGATTTAGTGGAAATG
GGAGTACGACACCAGTTGCATCCTGTGTCAAAAGGTCTTCGAACGTATTTGCC
GCCCGCATGTCATACGATGTCAACATATGAGAAAAAAAGTTTTTGTCATTGTC
TGAAAAATGTCAAAGTCCCACAAGGATACTCTTCAAATATCAAGAGCCTTGT
ATCAGTGGATGAAATGAAATTGGTGGGGTTGAAGTCCCATGATTGTCACGTT
TTGATGCAACAATTATTGCCTGTTGCCATTCGTGGAATATTGCCTGACAAAGT
TAGGGTTGCAATAACTCGATTGTGTTTTTTCTTTAATGCAATCTGTAGCAAAG
TGATTGACCCTAAACAGTTGGATGATTTGGAAAATGAGGCTGCCATTATCATT
TGTCAATTGGAGATGTACTTTCCCCCAACTTTTTTTGACATAATGATTCACTTA
CTTGTTCATCTTGTTCGAGAAATACGTTTGTGTGGGCCTGTATATTTGCGGTG
GATGTATCCGGTTGAGCGGTACATGAAGGTGTTGAAAAGTTACACGAAGAAT
CAATATAGGCCAGAAGCAAGCATTGTTGAAAGGTACGTGGCAGAAGAAGCT
ATTGAGTTTTGCTCTACTTACATCGAAGATGCATCACCTGTTGGTATTCCTGA

AAGTCGTCATGAAGCTACACGACAAGGTAGGGGAACGCGAGGATTCAATGTT
GTAACCATGGATCGCCAGAAACTATCACAAGCGCATTTGTATGTACTTAACA
ACACAGCTGAGGTAATACCATACATAGATGCTCACAAAGAATATGTGGCAGC
TTCTCACCCAAACATGAATATGATGAGGGTGTTGCAGGAACACAATAGAAGT
TTCATTAATTGGTTTAGAAATACAATATTTGCTAGCGACAGTGCTTCTAAGAC
ATTATCATTACTAGCTGTTGGGCCGAATCTTAATGTCCTCACTTGGAAGGGTT
ATGACATCAACAATTATTCCTTCTACACAAAGTCACAAGATGATAAAAGTAC
CGTGCAAAATAGTGGGGTCATGATTGATGCTCATTCAGACCACTTTAGTCGTG
CATCGGATAACAATCCTATTCGAGCTTCCATGGCTTATTATGGAGTCATAACC
GATATCTGGGAGCTAGACTATGGTGAATTTAGAGTGCCTGTTTTCAAGTGCCA
ATGGGTTAATGGAAATGTCGGAGTCCGTCAAGACAAATTGGGTTTTACTTTG
GTTGACCTTCAAAGGATTGGTTACAAGGACGAGCCTTTCATCATGGCAGCAC
AAGCAAGACAAGTGTTTTATGTAGAAGATCCTAGTGACTCAACATGGTCAGT
TGTACTTCAAGGGAAAACAAGTGGTATCCCTGCCGATACTGACCAAGCAACC
CTTGATGTTAACGAAATCCCTACGTTTGCACAACAAATGCCTTCGATAAATGC
TGAAAACGACGACGATGATGTGTATGCAAATCGTATCGATCATGATGAAGGT
TTATGGGAAAATATGGCAACTTAAATGCGAACCCATGGCAACACCCCCGACA
TCCCCTCCACCTCCTACATCCCCTCCACCTGCTGATTCACCAAGCGCAATCTC
AAAACCGAAGACTCGACAAGCAACCAGGTTGAGGAAATTGACTGCAAGAAC
CTTGGATCAACCACGACCAATTGTCAACGTCAACCCCGTTACTGGTCGAGGTT
CTGGTTCGGAAAAAGATAAATTTCACAGTTACTTGGGGGTAGTGGCACGGGA
GAAAATCCCTATTGTGCATTCATCTTGGAAAGTTGTCCCAGAATCACTTAAAA
ATATTGTATGGAATGACATTTTGGGAAAATTTGACATCCCGGAAGGAACTGC
TGCCAAGAAGAAGGTCATGTCTACTGTTGCGACTAGATGGAGGCAATTTAAG
TCCTCCCTGACCAGTAGATATATATATGCTGAGAAACATGGTGAAGATAACC
CTGATGCAGCTTCTAAGTATGGTATGGAGCAGCAAACATGGGAGCAATTTGC
AAAGAGTCGACAGACCCCAACTTGGCAGGGAATTCGGAAAAAAGCACAGGA
GATCCAAAAATTCAATGACTCCCCTCATTTATTGTCTCGTGGAGGGTATGAAC
TTATGGAAAAAAAATTGATGGAAGAGAAAATGAAGACAAGACAAAGGCAAG
CTGAGTGTACAGAAAATACACCGATGGTCGTAGACCCTCCATCCCCAATTGC
AAGACATGTTAAGTGGAAGATGGCTAGAACAAACAAATATGGAAAAATGAC
ATCTGCAGCAGCTCAACAAATCTCTGACAAAATTGATGAATTAGAAGAACAA
AGCACACAAGGTACGTTTGTGCCGCATGGCCGGAACGACATATTGAACACTG
CGCTTGGCCGTGAAGAGCATCCTGGTCGTGTCCTTGCTGCTGGACATGGTGTC
ACCATTAGTAGTTACTTTGGACAGCGTTCAAGTGCCTCTAATAGTTCTGCTGC
TACGATAACCCCGGATCAGTTGGTTCAAATCATAGGTAATCTCAAGCAAGAG
TGGACAAAAGAGGTAGAAGATGCAAGCAAACAAAAAATGGACATGCTGCAA
AAGGAGTTGGATGCAATCAAGACTGAGTTGTCCCAAATGCAAACTCAACAGT
CAGCCCCTGTACAACCGGCTAACCCTAATGTGTTGATTGCACGTGTTAGCACC
AAAGAAAGTTGTGCAGAAGCTGTTGCAAATGTTGTTGCTGGGGACCCATCTG
CGGTTGAGGAGAATACCATGGGATTGTATGTTGTTTGTGGCGACAGTAAACA
ATTGGTGGCCTTAGGAAAGGTGTATCAAGTTGGCGGCATGATACACAATGTT
CCTTACGCAGATGAAGTCGTGAGGGTTTCTGTGATTACTGTTTATGATGGTGA
TGCAAGGGTCCCAATTCCCACACCTGAGATTGAATACGTTAGGGAGGCCATG
AACACATTCATTGGCTGGCCAACTAATCTTGTCAAACCTTTCTCCGCTGATTC
CAATCAAGATGTAAGGAATCCAAAAGGACATGTTGATCGGTCAAATGCAGGT

GATGCAATGGATCCACTTGGAGAAATCATGAAAATACTTTATGAAGTGTATA
TGAATCCAGTGGAACTCCCGTGGGAGGCTAGCCGATTTGGAATTCCAAATAT
AGATGCCAAATTTTACATCACACATGCTGATATGGCTGAAATAATATCAGGT
CACAAGTGTTTAAACATTTCTATACTGCAACTATGGATGATGTATTTGGATGA
GTGTGCTACAAGCAGAGGTGATGGCTCAGTGTATGGCTTCCTTGAGCCTCAAT
CAATACACATTGGTAAGGAGGACCGTCAACAATGTCAACTTTATATTGAGAC
ATGGGTGAAGGAATCACAACGATGCTTGTACTTAGGAGCATACTTGCATCAG
TCACATTGGCAACTATTTGTTCTCTGTCCTAGGGAAAACATGGTTGTTTGGTT
TTGTTCGTTGCGAAAGAAGCCTGATGTTAACATAAAAGCCGTAATAAATAGT
GCAATGAAGACAATAAGTAGTTCTTTGGAAGGCATGTCTCAGCAAGGTCCAC
CTCGGTGGATTGAACCCAAGAGTCATGTTCAAAGTGGAGGGTACGAGTGTGG
ATACTATGTGATGCATTGGATGTGGTGCATCGTTAGTGGTCGTTTGAAGGATG
ACTGGAACAGGTGGTTCTCGGATGGATCAGCATTAGATGTGGAGGCCATGAC
AATAATTCGAAAGAATTGGGCAACTTACTTTTTAGCTATTAGAAATAACAGA
TGCTAAATATGATGTAGATTATTATGAATGACTACATTTTCCTTTAATGACAC
CCTTTAGTGGTATATTTTAATGAATTGTTTCATGTCACATTAATGTTTTTTAAA
AACCTACGTAATGGTTTAGTAAGGAGTTTATGTATTCTGAAATTGTTTTGGTT
TGTTGTAGTCTCGTATTAGAAAATATATATTTTGATAGGATGAGTGCAGTTTT
CTTAGAAATTGTACTCCTACTATGTATGTACTGCACAGTTGCACCTTCCATCA
ACTTAACAACACGTAGTTACTTGTGATAGCACTTGTATTATTTTTATTCCCATT
ACCGTAAGGCCTGAAGTGGTAAGTAGATAATGCCCTGCGTGTAGCACATAAT
TTCTACTTTGGGTATAGCCTTGCAAGCGAATGTTGTTGTTTGACCTTCTAGCA
CAGGAAAAACAATGGTTGCAAAGGCTGTGGCAACTGAAGCCGGAGGAAAAG
CAATATCCCAATAGGGTCACATGCAAAGAGTGTAGTAGCCATTTCAGCCTCT
GAGTGCTAAGGAAGCGCATGAAGTAAAAATAATGTGTACATATCACTTCTCT
CAAAGCCCAGATTAATTTTACTTTTAGAGTGAGTGATACAGATGAGGATGTCT
TGCAAATATTTTTTAAGGAGAGAGAATTAAATGGGGATTTTATATCAAGAGC
TTCCGATTTATTATGGAGAAGAGATTTCAGAAGTTCTGGTGATTATGATATTA
GCGAGCTCACCGACAACACTTCTCAACAAATAGAGCAGATCATAGAGACTGA
CAGTGATGGTGGTTTGTTGAAACTTACAAGAACCCAAGAGTGGCTAACAGGT
GACAATTCTCCACCAATAAACAAGAAGGTGACTGCTAAGGCATTACAGGACA
GCAGTGCAAGACGCATGAAACTGAACATGCTCAAATATGAATCTCTCAAGAG
GGAATTACTGCTTCTATCTGTGGGTATTGGACTGGCTTGTAGTGGATATTGCT
TGGTTATTTTTTCCGTACAGGCTGCTATAAGTTATGCGATTGGAGTCCTTTTCA
GGTTGAAACCTGGCAAGACATATTTGCCGGTGGCATCAATGACAGTGACATC
ATCTCCAACCTGGGAATGGAAATGAATCATAAGCAGAAAGTAACAAAAAGC
GCAAGGTGTTAGAAAGCAAATTAAGCACTGCTACTGCTAGGGGGTTTCAATT
CATCAAATACCCTGAAGTTAGGATTAACTGCAACAATGATGCCATCTTCCAC
GTAAACATCAGCCATCTGTTGGTGGTGACCATTCACAACCGTGCCTCCCTTGA
TCAACAACTTGGATGATGGAATTTCAGTCCCAGCATCACAAAACTGACTCAC
TCGAAAAGGACAAACATGAATTAAACATAGAACTAGTGGAAATGAAAATCT
CAGCTCAGTACTAATGATCATAGCTACTTAAAACAGTTAAGAATCGAAGCTC
TAACTCTTGGAAAACGCAGAACGGGTCACTAAAAGAACAAGCGCACTTGAAC
TTCTATGGTTGATGTAAGGATGGATGTGCGGACTAATTCTCTCGTCAGGCCAT
CAGGGACAATTGACAAAGATAAGGAAAAGCTACGGATTGCCAACAATGGTG
TCGTTCAGAGTGAAGAACAAACCTTACCTATTGGAGGTGATGGTTGGGAAAA

GTCAAAAATGAAGAAGAAGCGTTCCTGTATCAAACTAGATGTTTCTCCCAGT
ACAACATTGACTAAACCTGTTAACACCTTCCAAGAAACTAAACAGGGAATGC
AACAAAGACTTGCTACCGATTCGCGATTCTTTCAGGTCAGTAGTTTGTAATGG
AACTATTGGAGTTGGAAAATCAGATGGTATCTCTCAACAAACTGGGTTGGGC
ATACGAGCTTCTACCCCTAGAAACAACCAAGATAATAATTCCCTTGTCAATG
ATAGGAGGGGTCGTCCTGTTAGTTCAGACAAGGAAAGGGTGAACTTCAGAGT
TGTAAACAAGTCTTTTCTGACTTGGAGCGTTCAGTGTGTAGAATGGTCCATCG
ACAGGTTGCCACAATTGCTTGGTTGGAAGCTGATTCTGTTTGTGGCAGCCACT
CTATATGACTTCACA
