# Supplementary material for: A putative autonomous 20.5 kb-CACTA transposon insertion in an F3'H allele identifies a new CACTA transposon subfamily in Glycine max
Source: BMC Plant Biol. 2008 Dec 2;8:124. doi: 10.1186/1471-2229-8-124 (PMC2613891; doi:10.1186/1471-2229-8-124)
Supplement: Additional file 8 — Tgmt* Mosaic-transcript sequence. The sequence of the largest (2,572 bp) cDNA clone, No. 37, amplified with primers -1 and -7 contains all 14 exons of the Gene-1 3'-end and the 346 bp (1/9) portion of Exon-1. Purple letters represent Exon-1 bases, highlighted yellow are Intron-10 bases and orange letters are Exons 11–24 bases. Highlighted pink and green are primer 1 and 7 sequences respectively. [file 1471-2229-8-124-S8.pdf]

Additional file 8: ***Tgmt**** **Mosaic-transcript sequence** (2,572 bp = clone #37)

AACAGCACGCATAACTGAAGAGTACGAAAATGGGGTTGAAGGTTTCTTAAAA
TTTGCTAAAGATAATGCATCCGACAATGGTGGACTATACTTTTGTCCTTGTGT
TAAATGTTTGAATGGGCGACGACAATGTTTGGATGACATTAGAACACACCTT
ATCTGTGATGGTATCTGTCCTACTTATACAAAATGGATATGGCATGGTGAGTT
ACCAGAAATGTCATCAACCCCTCCAACTGCTCCAACTGATGAACAAGTCGGT
GATCAAATAGAAGACATGCTACGTGATCTTGGACAAGAGGGTTTTAGGCAAG
CAAATGCACCGTATTATGACACCTTACATAATGTCTTCCATTGTTAATTTTGT
AGTGGTTCTCGGATGGATCAGCATTAGATGTGGAGGCCATGACAATAATTCG
AAAGAATTGGGCAACTTACTTTTTAGCTATTAGAAATAACAGATGCTAAATA
TGATGTAGATTATTATGAATGACTACATTTTCCTTTAATGACACCCTTTAGTG
GTATATTTTAATGAATTGTTTCATGTCACATTAATGTTTTTTAAAAACCTACGT
AATGGTTTAGTAAGGAGTTTATGTATTCTGAAATTGTTTTGGTTTGTTGTAGTC
TCGTATTAGAAAATATATATTTTGATAGGATGAGTGCAGTTTTCTTAGAAATT
GTACTCCTACTATGTATGTACTGCACAGTTGCACCTTCCATCAACTTAACAAC
ACGTAGTTACTTGTGATAGCACTTGTATTATTTTTATTCCCATTACCGTAAGGC
CTGAAGTGGTAAGTAGATAATGCCCTGCGTGTAGCACATAATTTCTACTTTGG
GTATAGCCTTGCAAGCGAATGTTGTTGTTTGACCTTCTAGCACAGGAAAAAC
AATGGTTGCAAAGGCTGTGGCAACTGAAGCCGGAGGAAAAGCAATATCCCA
ATAGGGTCACATGCAAAGAGTGTAGTAGCCATTTCAGCCTCTGAGTGCTAAG
GAAGCGCATGAAGTAAAAATAATGTGTACATATCACTTCTCTCAAAGCCCAG
ATTAATTTTACTTTTAGAGTGAGTGATACAGATGAGGATGTCTTGCAAATATT
TTTTAAGGAGAGAGAATTAAATGGGGATTTTATATCAAGAGCTTCCGATTTAT
TATGGAGAAGAGATTTCAGAAGTTCTGGTGATTATGATATTAGCGAGCTCAC
CGACAACACTTCTCAACAAATAGAGCAGATCATAGAGACTGACAGTGATGGT
GGTTTGTTGAAACTTACAAGAACCCAAGAGTGGCTAACAGGTGACAATTCTC
CACCAATAAACAAGAAGGTGACTGCTAAGGCATTACAGGACAGCAGTGCAA
GACGCATGAAACTGAACATGCTCAAATATGAATCTCTCAAGAGGGAATTACT
GCTTCTATCTGTGGGTATTGGACTGGCTTGTAGTGGATATTGCTTGGTTATTTT
TTCCGTACAGGCTGCTATAAGTTATGCGATTGGAGTCCTTTTCAGGTTGAAAC
CTGGCAAGACATATTTGCCGGTGGCATCAATGACAGTGACATCATCTCCAAC
CTGGGAATGGAAATGAATCATAAGCAGAAAGTAACAAAAAGCGCAAGGTGT
TAGAAAGCAAATTAAGCACTGCTACTGCTAGGGGGTTTCAATTCATCAAATA
CCCTGAAGTTAGGATTAACTGCAACAATGATGCCATCTTCCACGTAAACATC
AGCCATCTGTTGGTGGTGACCATTCACAACCGTGCCTCCCTTGATCAACAACT
TGGATGATGGAATTTCAGTCCCAGCATCACAAAACTGACTCACTCGAAAAGG
ACAAACATGAATTAAACATAGAACTAGTGGAAATGAAAATCTCAGCTCAGTA
CTAATGATCATAGCTACTTAAAACAGTTAAGAATCGAAGCTCTAACTCTTGG
AAAACGCAGAACGGGTCACTAAAAGAACAAGCGCACTTGAACTTCTATGGTT
GATGTAAGGATGGATGTGCGGACTAATTCTCTCGTCAGGCCATCAGGGACAA
TTGACAAAGATAAGGAAAAGCTACGGATTGCCAACAATGGTGTCGTTCAGAG
TGAAGAACAAACCTTACCTATTGGAGGTGATGGTTGGGAAAAGTCAAAAATG
AAGAAGAAGCGTTCCTGTATCAAACTAGATGTTTCTCCCAGTACAACATTGA
CTAAACCTGTTAACACCTTCCAAGAAACTAAACAGGGAATGCAACAAAGACT
TGCTACCGATTCGCGATTCTTTCAGGTCAGTAGTTTGTAATGGAACTATTGGA

GTTGGAAAATCAGATGGTATCTCTCAACAAACTGGGTTGGGCATACGAGCTT
CTACCCCTAGAAACAACCAAGATAATAATTCCCTTGTCAATGATAGGAGGGG
TCGTCCTGTTAGTTCAGACAAGGAAAGGGTGAACTTCAGAGTTGTAAACAAG
TCTTTTCTGACTTGGAGCGTTCAGTGTGTAGAATGGTCCATCGACAGGTTGCC
ACAATTGCTTGGTTGGAAGCTGATTCTGTTTGTGGCAGCCACTCTATATGACT
TCACA

Orf: 189 aa x100 =18.9 kDa

```
CVHITSLKAQINFTFRVSDTDEDVLQIFFKERELNGDFISRASDLLWRRDFRSSGDYDISELTDNTSQQIE
QIIETDSDGGLLKLTRTQEWLTGDNSPPINKKVTAKALQDSSARRMKLNMLKYESLKRELLLLSVGIGLAC
SGYCLVIFSVQAAISYAIGVLFRLKPGKTYLPVASMTVTSSPTWEWK
```
