# Supplementary material for: A putative autonomous 20.5 kb-CACTA transposon insertion in an F3'H allele identifies a new CACTA transposon subfamily in Glycine max
Source: BMC Plant Biol. 2008 Dec 2;8:124. doi: 10.1186/1471-2229-8-124 (PMC2613891; doi:10.1186/1471-2229-8-124)
Supplement: Additional file 9 — Tgmt* genomic sequence (20,544 bp). The reverse complement of Tgmt* genomic sequence (20,544 bp) is shown. Exons are in orange letters. Purple letters are the portion of Exon-1 that splices with Intron-10 to form the mosaic-transcripts. The 23 bases of Intron-10 that are part of Mosaic transcripts are shown in yellow. Also in yellow are the bases of Intron-19 and -20 that are not spliced out in cDNA clone 47. The direct and reverse subterminal repeats are highlighted in yellow and green respectively. The 13 bp CACTA terminal inverted repeats are highlighted in blue. Sequences of Primers 1, 3 and 5 are highlighted in pink and those of primers 2, 4, 6 and 7 are highlighted in green. [file 1471-2229-8-124-S9.pdf]

Additional file 9: *Tgmt genomic sequence** (20,544 bp)

CACTACTAGAATAATGTTTTTTTACGACGTAGTATTTAAGTCGGTTATCCAGA
ACCGCCTTAAATAGTGGCGCGGTGGCAAACTTGTAATTATCAAAGCTGAGAA
TAATTTTTTACAACGTAAATTCTAAGGCGGTTATAAATAACCATCTTAGTAAC
TCGCTGGGTGGCATTTTTGTAAATATCAAAAAATGTTCAACGACGGGTTTGGA
TAAGGAACCGCCTTTGTTTTATTTCCCTTAAATTAGGGATTTTACAGCCATCCT
TTTCCATTCTTCACCCAGCGTCTCCTTYGATCTTTACCAGCATCCCTTTCCACT
CTTCACCCACTCTTCACCCACGACGAGAAGTTTGTGACCCATCATCACTAAGC
CTTGTCGAACAGAAGTGTGTCGTGCCTTGTCTTCTGATTTTGCCCAATCCAAG
CTGCGACCTTCAAAGCTATTTCCGCCCACCACGGTAAGCATCTTACAAATCTA
CTCCTATCAAAGCCTTCCTTGTGTCAACCACAACGAAACCTATCTACTTTCCT
ATTATTTTGGCCACTTTTTCGGTAACTCCTAATTTCAAATCTCGTCAAGTGCTA
CACCGAAAGTTCGTTGCATATCTCAAATCTATTAGATAGAACTGCTGTTGTAA
TTTTTATTTAGGGATTGCATGTTCTTTGGATTTGAGATTGTGTGGATGTACTGT
TGGTATTCAATTTTAGGTAGGCTTGCTTCTGATTTTCCTCTGTATACAAAGAG
YTAATATGTTTTGTTTCTCTTGCCAGAAAAAACAATGGAAATACGTAGTGATT
TAATATTTTTCTTGTTTTAGCTTGCAAATTATCTGAAAGATATTGGTGTTGTGA
AGAAGGGAGATGCTATAATTATTTTTCTTGTTTCAGCTTGTTTAGTCATGTCA
AAATCTCCAGGTTGCTTGATATGTTCCACTTGTCTTTTAAGTTAGTAATATAAT
TTTTTATTTTTTTTTGTTATGTATATGGTTCTGTCCATAAATCACAATATATAC
TGAAAACTGGAATCCATACTTGTATGAGGATGAAGGATTTATTTTAGGAAAT
AGGAACAAGGATACCGTGTATCAGTGCTTCAGCGATATAAAAGCTACCTTTG
TAGGGTTTTGTTTATGTGAGGAGAAATTAGCTACTTTAGTTAAATATAATAAT
CGATTGTTATTGTTAGTGTTTGATGGTATCTCAACTCTCAAGCTCTTGCTCATG
TGTGTCTTAACTAATGGCTTCAACTCTCTATTTGTGTCTCCTTATCATTTAGGA
TTTCTTGAAAAGACATGTGTTTATGCGCCTCAGTTCTTTTAGTTCCTTGAATTC
TCGAATAACATTTCTTCTACAATCTCTGCAAAATCTCTTGCTATCGAACCTGA
TAGATATGGTAACTCATTAATAAAAACCAAAAGCATAAAACATCCTTATCCT
TAAAAACATACAATTGTGTGCATATATATATAGAGAAAAAGGGATCTCACCA
AATACTATAAAGCTTTGAAGCAAAGCTTCACATATAGTTTCTGATGATGAAA
AAATAGATTAAATCATACTAGGAATGAATGAATAGGCATTGTGTTTATGTATT
CAAATGAGAATTAGTACAAAATGTTCAAAAAAATGACTAAAAATTAAAAAC
AAAAATCAGTAGTCTAACTCTAATAACACAAAAAGACCCACCTGATGATAGA
AAGTAGCAAGTTCTTTGACCCACTACAATGAGACAGCACCAGACACAATGTT
GGAAATTTTAAATAAACAGTTTCCCTTAATATTTTTTTGCTGACAGAACATGT
AACTCCAGAGACATAACACTCAAAACTTCAATAAACCAGTAATAACAGGCAA
AAATAAAATAGTGCAGTGAATCTATAATAGATAGTTTTTCCAATTTATATCAG
AAAGATAAAGTTAACATTCTAATCTACCAAGGTGCCTTTGTAAAATCACTTTT
GAGAGATTTGCTTCATTCATTTTCAATTTTGCTCCCTTTCTTCAAACCAAGCAT
ACAATAATACTTTAGAATCCCTTGTTTCCATATATATGCAACTGATGGATCAT
TTTCTAACTCGATGTCTTTGCTCATTACCCTCTATATAATTTATAATTAGTTCT
TTGCTAATTAGACAGAAAAAATGTATCATTACTCTTTGAATTGATCTTAGGTT
AGTTTTTCTCGAACATGATCATTTTAGCAATTTATTTATCCATCTCCACATTAA
GAAAGGAAAAGGGCTATACATGTTGAACTTCATTGCAGTTCTACATTATTTTT
GCAGCCTGCAAAACTAGCGTTGCGGATTACATGTTAGCTGTTGTTGAAAATTA

AGAGGCACATATCAGACCTTCATTTTCTAAGGTAACCTGTAATTGCATTTTTT
TAGCATTTTTATTTCTTACTTTTTGTGTCTCTAGTTTTTTCTCTAGTTATGGTTT
GTTTGATAAGTTTGAATGTTTTGTATTCTTTGATATTTTACTATGTGTGAGAGA
GATCGATATATTAGATGTTGATGTTGCTATAATTATTAATTTTTACTCTAGAG
GAACTTTTCTTTTTCTCTTTTTATGGGGTGAGAGTGTTAGAACTGTTATCTTGA
TCATGAAAATTGAAAAGGACAAATAGTAGTTTTCTGTTTTCATATGTTTTAAT
CATTTGATGTGTGTTAAGATAAATCATATGCATAGTATGAATTGGTTTTTTGG
TAAATAGGTATAGACGTATGCACAGCGAAAATACCTTAGCATTTTATTAAAA
AATATCATTTTTGTTTTGATCATCTCGTCTGGCCTTATGGAGTGATGCCTACTG
ACTCAAAAAGACGTGAAGAATGGAATCACATTTTGGTTTTTATAACTCAAAT
ATCCCAGCAATTTTACAGTAATATCAATTTCCCTGGATTTCAATAAGATTTAT
ATTTTTTTTGTTAACATAAAAACAAATATTAATATATTTTCAATCCTATCTAAC
AAAAGGTCAGATATGTCCCAAAAAGAATGCCTGACAATTTATTTATAGAGAT
TACAATTTATAATATTTTCTGTAAAAAAATAAATAAAATCAAATAAACAATA
AAAGTTATCTCTCTTTTATATAAAAAAGAAATATTTATAATTATATATATGAT
ATGAACTGTATGTAAAATAGTTATATATGCTAATACTTTTGATACTTATGTGA
TGTCCTTGGTGATTCCTCATGAGTATTCAAGAACGAAATAAAGCATTTTTATT
TAAATATTCTTTTGTTATATCTCTTCTTTTAGCTTAATTTGATTTGTAACTGTG
ACTTCAACTAACCCTGTCAAAAAAAAAAAAAAAAAGTAGTGTGACTTCAACCA
ACAATTTTTTTTATCATATTACTTTCTTTTTAGACAACCTAATTTTCTATAGAG
ATTACGTGATTTGATATGAACAAGGTACGTAAAAGCTGAAACAGATTCTTGT
TTCATAAGCTTTGGGTCGTATAGATTTTCTTATTTTTCTTATAATATTCGATTT
ATTTTCTTATGGTGGTTTTTAGTTCCACTTGATTTGCTTAAAACGTGCTGTAGA
CATTGGTCCAAAACGAGATTGAAGGAGGTGGGGTACCTATAAAGGTACTCTA
AAGTCCAAATTAAAATGTCAATTAGCAAAATGATTATTGTGAACAGTGTGCA
CATCTAGTACCAATCTTAGTGGTTTATTTATTGGGGTAACTCACAAAAAGGTA
GTCATGCGTGGCCATAAGGTAATCTCCTTTTAATTTACTTGTCTGGTGTGAAC
AAGTATATATCAGGGATAATGGATATCAGATGGTGATTGACCATTTCAATAC
TCCCCGTTAGGCCTTGCTAGTGCTATTAGTTCTCTGCTGCTTAATCCGGTATAG
TTAATTTGATATTAATATGTTTGGTTGGGATGCAATTTGTTTATGGAATAAGG
ATGGAAAAGAGTTTGAATAAAGCGGAACTTAATATCATGTTTTATTTTGAACT
GTATGTATTCATAATAATATTTTTATTGGATATAATATTTTTTAATTGGCAAAG
CAAGCATATAATAGATAATTAAGATGGTACAAGATATATCTTAACACAGACG
AAATTGGAACATATTACACAACACTTTCATACAAAAGTATAGAATCCATAGA
CCAATTAAAAAATATAAAGAAAAAACAATAGTTAGCCACAACGCAGTACTG
AATTTGAGTTGATAGGCACTGCCAACGTTGCTTGTGTTGCTAAATTGAGGCTT
GAGGAAATTTGGATCTGAACTTAAAATTCTGGCTTTGTATTGTTAACTCATCT
CATAATGTGGCTTTGTGTTTCATTTTTATTTGTTACAAAACTGTACTCCTAGTA
TGGTTGTAAATGATTTTTATGTTTTTAGGTTGTACTGTCAAACGTTGCGTAGTC
TGTTGAGGATAGAAGTAGGCAGGTAGTTGCTGGACTGAAGTTACACTCATCC
ATTCATCAGCAGTGTCACCACAAGCAAAGGTTTAGTTAGTAGCCTATTTAGA
GGCATGTATGTGATTCTGAAACTTATGTCCTCATTTATTACGTTGTTGCTTGAT
ATGTAGCCTAGAGATTCCTAAATATTAGCAGTTAGTTACTAGTTAGAGAGTAT
GGAAAGACGGGTATAGCTTAAATAGGAGGTTAAATAGGGAGTGGGATTCAG
CTTTGTTAGAAAAGGGTGTAGTGGCTTGGTTGTAAATAGGCGATATATATATA
AACTTTACAACAGATAACAAACACTTTTTCTTAACAATAAGAACATGTGGGC

TGAGCTCATATACTGAAGAGTAAATCTTATTGACTTCCATGATAGTAAGTTGA
CTCTCAACACTATGAAAACAAGTGGTTTAGTGAGATAGTTATCAATTTTTTTA
GTATCATAGAAATGACATAGAAGAACTTGGTAGAAAATGATGGCATAATAAG
TTATTTTAAGAATTTCTATTTTCCTTTGTTTCGACAAACAGTAGAGAAAATAA
TTTATGAATTTAGATGAAATCATCAAAGTGCATCTAATTAGAAATAAATATCT
ATAGCGTAAATTTTTTTAAAATTAATAAATAAGTATTATGATAAGAAAAAAA
TAATGGAACAAAACAATACAATTGTTAAATTAAAAATAATTACTATCTTTTGA
TGAAGTCATCAAATTAGAATAAACTGTTTTTGATTTGGGACTCAGTGATAACA
TGTTAATATGTTAAGACTTTTTTCACAAAATATTATGACAGAACTAATTAGAA
GCATTTCCTTAATTTTAGAACTTTTTTCAATCGAAACTGGAGTTTCATCTTGGT
AACCAAAGAAAAAACTCCAATTGTAATTGAAGAACAATACTAAAAGCACCTA
AAGAACTATACTAAGTTTTGTCAAAAATATTGTTCAAATTATAGACATTATTT
CCTTTCCCATTGTTCAATAGACTACTCTAGACTACATATGAGAGCTATAAGAT
TAGTTTAGTGGTTGAAGAGAGAAAGAGAGAGGGAGAGGTCACTCACACTCCC
TTTTTGGCAGTGTTTTAATACAACAATAGTATCTGTTATGTTTTTCAATTTTGA
ACATTTTGTTAATTTCCTTCATTTAACAATGAATACTTTATTTGTATGCCATTT
TCACATTTATGTCGTTATTTATTATATTGTTGGTTCACATGAAACTATTGTGTA
GTACTACATTCTACTTCATTGAGCGAGCCATAGTTCCCCGTTCGAGGTTCAAT
ACAACGATTAATACGGATAGTTTGGATTAATTGTTGTATTGAATCTTGAATTG
TCCGTTTGGACAGTTTGGGAAGACAAACTTTTTTTACTTCATTTTCACTTAAA
ATTTAACTACGTTGTGTTATTTTAACTAGATTCGGACACTGCATCTTGCTTTGT
TCTCCAGGTGCATACATGATTGGGGTGAATCGTTGTCACCGCTTCCATGTCGT
GTACTTGGAGACCAAAGCGAAATGCTGCCGAAATTTAGTTAAAAATAACACA
ACATAGCTAACTTTTAAGTGAAAATGAAGTAAAAAAATGTTTTCCTGAATGG
GGTAGGGTCACACCTTTGGTTTAATAAAAGAGAGGTTCACATGCATATCGAA
TAATGTATGTCGCCTGACAGTACAACAAAGATGGATCGGCATTGGATGAAAA
CAGCACGCATAACTGAAGAGTACGAAAATGGGGTTGAAGGTTTCTTAAAATT
TGCTAAAGATAATGCATCCGACAATGGTGGACTATACTTTTGTCCTTGTGTTA
AATGTTTGAATGGGCGACGACAATGTTTGGATGACATTAGAACACACCTTAT
CTGTGATGGTATCTGTCCTACTTATACAAAATGGATATGGCATGGTGAGTTAC
CAGAAATGTCATCAACCCCTCCAACTGCTCCAACTGATGAACAAGTCGGTGA
TCAAATAGAAGACATGCTACGTGATCTTGGACAAGAGGGTTTAGGCAAGCA
AATGCACCGTATTATGACACCTTACATAATGATTCAAAGATTCCATTGTTTAT
TGGATGCACTAAGTACACACGGTTATCAGGGGTGTTAGCTCTGGTCAATTTGA
AAGCAAGATTTGGGTGGAGTGACAAAAGTTTCAATGAATTACTGTTGTTATT
GAAGAATATGCTTCCAGGAGATAACACGTTGCCAAAGACTCATTACGAGGCA
AAGAAGATATTATGTCCTGTTGGAATGGAATACCAAAAAATACATGCTTGCC
GTAATGATTGCATTTTGTATAGACATGAGTTTGCTGAATTGCGCAACTGCCCT
ACATGTGGGGTGTCACGCTACAAAGTGGGTTCTGGCGCTTCCAGTGAAGCTG
GATCCACATACATTGATCGGCCAGCAAAAGTGTGTTGGTATCTTCCAGTAATA
CCAAGGTTTAAGCGATTGTTTGCTAATGCAGAAGATGCAAAAAACCTAACAT
GGCATGTTGATGGTAGGACCAAAGATGGATTGCTCCGTCATCCTGCTGATTCT
CCTCAGTGGAAGAAAGTTGATCAGTTGTATCCAGTGTTTGCCGAAGATCCCA
GAAACCTAAGGGTTGGTCTCGCATCGGATGGAATGAATCCATTTCGAAGCTT
AAGTTGCAATCATAGTTCGTGGCCTGTTTTGTTGATCATTTACAACCTGCCTC
CTTGGTTGTGCATCAAGCGGAAGTACATAATGATGTCTATGATGATAGCCGG

TCCAAGACAACCAGGAAATGACATTGATGTGTATCTTGCTCCCTTGATTGAAG
ACCTAACAAAATTGTGGGTAGAAGGGGTTGATGTGTATGATGGGAATGCTCA
TGAGTCCTTCAGGTTGCGAGCTATGATTTTCTGCACCATTAATGACTTTCCAG
CATATGGGAATTTGAGTGGATATAGTGTGAAAGGCCACCTTGCTTGTCCCATT
TGTGAGAAAGACACAACTTACCTCCAATTAAAGCATGGCAAAAAAACTGTAT
ATACAAGACACCGTAGATTTTTACAACCTTTTCACCCATACAGGCGACTGAA
GAAAGCGTTTGATGGGACATCTGAGAATGACAGTGCATCAATTCCTTTGTCA
GGTGTTGAAGTTTTTGATCGTGTGAAGAACATTTGCAATATATATGGGAAGA
CACAAAAGAAAGATGGCGCTCCCAAAAACATTTGGAAGAAAAGGTCCATCTT
CTTTGATCTTCCATACTGGTGCAACTTAGATGTGAGACATTGTTTAGATGTGA
TGCATGTTGAAAAAAATGTCTGTGACAGTTTGGTTGGCACACTGCTTAACATT
AAAGGGAAGACAAAAGATGGTTTGAAATGTCGTCAAGATTTAGTGGAAATG
GGAGTACGACACCAGTTGCATCCTGTGTCAAAAGGTCTTCGAACGTATTTGCC
GCCCGCATGTCATACGATGTCAACATATGAGAAAAAAAGTTTTTGTCATTGTC
TGAAAAATGTCAAAGTCCCACAAGGATACTCTTCAAATATCAAGAGCCTTGT
ATCAGTGGATGAAATGAAATTGGTGGGGTTGAAGTCCCATGATTGTCACGTT
TTGATGCAACAATTATTGCCTGTTGCCATTCGTGGAATATTGCCTGACAAAGT
TAGGGTTGCAATAACTCGATTGTGTTTTTTCTTTAATGCAATCTGTAGCAAAG
TGATTGACCCTAAACAGTTGGATGATTTGGAAAATGAGGCTGCCATTATCATT
TGTCAATTGGAGATGTACTTTCCCCCAACTTTTTTTGACATAATGATTCACTTA
CTTGTTCATCTTGTTCGAGAAATACGTTTGTGTGGGCCTGTATATTTGCGGTG
GATGTATCCGGTTGAGCGGTACATGAAGGTGTTGAAAAGTTACACGAAGAAT
CAATATAGGCCAGAAGCAAGCATTGTTGAAAGGTACGTGGCAGAAGAAGCT
ATTGAGTTTTGCTCTACTTACATCGAAGATGCATCACCTGTTGGTATTCCTGA
AAGTCGTCATGAAGCTACACGACAAGGTAGGGGAACGCGAGGATTCAATGTT
GTAACCATGGATCGCCAGAAACTATCACAAGCGCATTTGTATGTACTTAACA
ACACAGCTGAGGTAATACCATACATAGATGCTCACAAAGAATATGTGGCAGC
TTCTCACCCAAACATGAATATGATGAGGGTGTTGCAGGAACACAATAGAAGT
TTCATTAATTGGTTTAGAAATACAATATTTGCTAGCGACAGTGCTTCTAAGAC
ATTATCATTACTAGCTGTTGGGCCGAATCTTAATGTCCTCACTTGGAAGGGTT
ATGACATCAACAATTATTCCTTCTACACAAAGTCACAAGATGATAAAAGTAC
CGTGCAAAATAGTGGGGTCATGATTGATGCTCATTCAGACCACTTTAGTCGTG
CATCGGATAACAATCCTATTCGAGCTTCCATGGCTTATTATGGAGTCATAACC
GATATCTGGGAGCTAGACTATGGTGAATTTAGAGTGCCTGTTTTCAAGTGCCA
ATGGGTTAATGGAAATGTCGGAGTCCGTCAAGACAAATTGGGTTTTACTTTG
GTTGACCTTCAAAGGATTGGTTACAAGGACGAGCCTTTCATCATGGCAGCAC
AAGCAAGACAAGTGTTTTATGTAGAAGATCCTAGTGACTCAACATGGTCAGT
TGTACTTCAAGGGAAAACAAGTGGTATCCCTGCCGATACTGACCAAGCAACC
CTTGATGTTAACGAAATCCCTACGTTTGCACAACAAATGCCTTCGATAAATGC
TGAAAACGACGACGATGATGTGTATGCAAATCGTATCGATCATGATGAAGGT
TTATGGGAAAATATGGCAACTTAAATGCGGTAAATAAGACAATAATGTTAAA
CTTGTCAATAGTCATTTTTTATTTAATGTTTCTTCTCTTTCATTACACTTTCATA
CTTACATGTTGTTCTAATCTAACATTTGGTTTCTTAATATTATTGCAGAACCCA
TGGCAACACCCCCGACATCCCCTCCACCTCCTACATCCCCTCCACCTGCTGAT
TCACCAAGCGCAATCTCAAAACCGAAGACTCGACAAGCAACCAGGTTGAGG
AAATTGACTGCAAGAACCTTGGATCAACCACGACCAATTGTCAACGTCAACC

CCGTTACTGGTCGAGGTTCTGGTTCGGAAAAAGATAAATTTCACAGTTACTTG
GGGGTAGTGGCACGGGAGAAAATCCCTATTGTGCATTCATCTTGGAAAGTTG
TCCCAGAATCACTTAAAAATATTGTATGGAATGACATTTTGGTAAGCGCACTT
AAATTAAAAATTTGATTTCATTTGTGTCTCCTTTAGGCCAAATTGTCATTTTTA
TCATTAGTGTATGACAAATTATTTGTTATTTCAGGGAAAATTTGACATCCCGG
AAGGAACTGCTGCCAAGAAGAAGGTCATGTCTACTGTTGCGACTAGATGGAG
GCAATTTAAGTCCTCCCTGACCAGTAGATATATATATGCTGAGAAACATGGT
GAAGATAACCCTGATGCAGCTTCTAAGTATGGTATGGAGCAGCAAACATGGG
AGCAATTTGCAAAGAGTCGACAGACCCCAACTTGGCAGGTTTGAATTGACTT
TATATTTCACAAGTTTTGTTACTTAAATTTTGTTAACATTATAACACAATGCAT
TGTTTTTGTTACTTCGGACAGGGAATTCGGAAAAAAGCACAGGAGATCCAAA
AATTCAATGACTCCCCTCATTTATTGTCTCGTGGAGGGTATGAACTTATGGAA
AAAAAATTGATGGAAGAGAAAATGAAGACAAGACAAAGGCAAGCTGAGTGT
ACAGAAAATACACCGATGGTCGTAGACCCTCCATCCCCAATTGCAAGACATG
TTAAGTGGAAGATGGCTAGAACAAACAAATATGGAAAAATGACATCTGCAG
CAGCTCAACAAATCTCTGACAAAATTGTAAGTGCAATTAAAGCCTTCGTGGC
AATTTGAATAGGTAGATGGTATATCATTTAACTTTGATCTGATTTTTGGCTATT
TATGACAGGATGAATTAGAAGAACAAAGCACACAAGGTACGTTTGTGCCGCA
TGGCCGGAACGACATATTGAACACTGCGCTTGGCCGTGAAGAGCATCCTGGT
CGTGTCCTTGCTGCTGGACATGGTGTCACCATTAGTAGTTACTTTGGACAGCG
TTCAAGTGCCTCTAATAGTTCTGCTGCTACGATAACCCCGGATCAGTTGGTTC
AAATCATAGGTAATCTCAAGCAAGAGTGGACAAAAGAGGTAGAAGATGCAA
GCAAACAAAAAATGGACATGCTGCAAAAGGAGTTGGATGCAATCAAGACTG
AGTTGTCCCAAATGCAAACTCAACAGTCAGCCCCTGTACAACCGGCTAACCC
TAATGTGTTGATTGCACGTGTTAGCACCAAAGAAAGTTGTGCAGAAGCTGTT
GCAAATGTTGTTGCTGGGGACCCATCTGCGGTTGAGGAGAATACCATGGGAT
TGTATGTTGTTTGTGGCGACAGTAAACAATTGGTGGCCTTAGGAAAGGTGTAT
CAAGTTGGCGGCATGATACACAATGTTCCTTACGCAGATGAAGTCGTGAGGG
TTTCTGTGATTACTGTTTATGATGGTGATGCAAGGGTCCCAATTCCCACACCT
GAGATTGAATACGTTAGGGAGGCCATGAACACATTCATTGGCTGGCCAACTA
ATCTTGTCAAACCTTTCTCCGCTGTAAGTGAAATGATTTTCCTTGTGGTTCCCC
TAAATTAAATGGCATATTCCAATAATGGTTGCTAAATTAAATTTAATATTTAT
TTTATAAAACTATATAGGATTCCAATCAAGATGTAAGGAATCCAAAAGGACA
TGTTGATCGGTCAAATGCAGGTGATGCAATGGATCCACTTGGAGAAATCATG
AAAATACTTTATGAAGTGTATATGAATCCAGTGGAACTCCCGTGGGAGGCTA
GCCGATTTGGAATTCCAAATATAGATGCCAAATTTTACATCACACATGCTGAT
ATGGCTGAAATAATATCAGGTCACAAGTGTTTAAACATTTCTATACTGCAACT
ATGGATGATGTAAGTCATTTAATTACAACCTTTAACCCTAAATGTTATCATAA
GCAACAAATTGTAAATGTAATACATTTGTGGTATAATGATTGTATTGTCTTTC
AAATAGGTATTTGGATGAGTGTGCTACAAGCAGAGGTGATGGCTCAGTGTAT
GGCTTCCTTGAGCCTCAATCAATACACATTGGTAAGGAGGACCGTCAACAAT
GTCAACTTTATATTGAGACATGGGTGAAGGAATCACAACGATGCTTGTACTT
AGGAGCATACTTGCATCAGTAAGTTAAATTTTTTTGTGGCATTTAACAAATGT
TATGATTTCTAAACTTGCTAATTATAATCATCAACTTCAGGTCACATTGGCAA
CTATTTGTTCTCTGTCCTAGGGAAAACATGGTTGTTTGGTTTTGTTCGTTGCGA
AAGAAGCCTGATGTTAACATAAAAGCCGTAATAAATAGGTACGAGTGTAATG

TACTTAAAGATAGCCGAGCTGTCATTGTTGTTGAATATGCAGCGTAAATGTTT
GTTATAAGTAACATCATATATGATTTCGTTTACTAGTGCAATGAAGACAATAA
GTAGTTCTTTGGAAGGCATGTCTCAGCAAGGTCCACCTCGGTGGATTGAACCC
AAGGTTAGATGGTTGTTTAGATGAACCCCTATGTAATTTTTAAAGGGTACAAG
TGATAATAGTTATTTTCACTGATAAATATAGAGTCATGTTCAAAGTGGAGGGT
ACGAGTGTGGATACTATGTGATGCATTGGATGTGGTGCATCGTTAGTGGTCGT
TTGAAGGATGACTGGAACAGGGTATATATACAAAGTCTAATTTCAATTTTCAT
GTTAGTTGATATTTGTTTAATTATTAATAATGTCTTCCATTGTTAATTTTGTAG
TGGTTCTCGGATGGATCAGCATTAGATGTGGAGGCCATGACAATAATTCGAA
AGAATTGGGCAACTTACTTTTTAGCTATTAGAAATAACAGATGCTAAATATG
ATGTAGATTATTATGAATGACTACATTTTCCTTTAATGACACCCTTTAGTGGT
ATATTTTAATGAATTGTTTCATGTCACATTAATGTTTTTTAAAAACCTACGTAA
TGGTTTAGTAAGGAGTTTATGTATTCTGAAATTGTTTTGGTTTGTTGTAGTCTC
GTATTAGAAAATATATATTTTGATAGGATGAGTGCAGTTTTCTTAGAAATTGT
ACTCCTACTATGTATGTACTGCACAGTTGCACCTTCCATCAACTTAACAACAC
GTAGTTACTTGTGATAGCACTTGTATTATTTTTATTCCCATTACCGTAAGGCCT
GAAGTGGTAAGTAGATAATGCCCTGCGTGTAGCACATAATTTCTACTTTGGGT
ATAGCCTTGCAAGCGAATGTTGTTGTTTGACCTTCTAGCACAGGAAAAACAA
TGGTTGCAAAGGCTGTGGCAACTGAAGCCGGAGGAAAAGCAATATCCCAATA
GGTATGTTAACAGATTATTTTAACTCTTTGAAATGTTTGCAGTCAGCTGAGTT
GACTTCCATGTCAATGTAATCATGTTCTGCAGGGTCACATGCAAAGAGTGTA
GTAGCCATTTCAGCCTCTGAGGTACACAACATAGACTTTATACTCTTTATAAT
AACTAATCTTATTTCAATCTTTTTCATTCAATGAAATGAATGAGATAGAATTT
TATTTGATTTACCAGTGCTAAGGAAGCGCATGAAGTAAAAATAATGTGTACA
TATCACTTCTCTCAAAGCCCAGATTAATTTTACTTTTAGAGGTTAGCATAGAT
TATTCCCCCTTCTTAGTATAAAAATAAATAAATTATCTCCTTGCTCTTCTTTAA
ATTATCTCCCAGCATATGTAATACTTAATCCTTGGCACAGTATCTTTTTTCTTT
TCACTCACTCCTTGTTTCTGCTGAAAGCTAATTGGTTGAAACTCATTTTTTAGT
GAGTGATACAGATGAGGATGTCTTGCAAATATTTTTTAAGGAGAGAGAATTA
AATGGGGATTTTATATCAAGAGCTTCCGATTTATTATGGAGAAGAGATTTCAG
AAGTTCTGGTGATTATGATATTAGCGAGCTCACCGACAACACTTCTCAACAA
ATAGAGCAGGTGCAGCAGACTCATTATATCTAATTTCGAACTATTCCTATCCA
ATTTTAAAGATTCTATCAAGTGTGTCAGTTTTGGCAATTTGTTGCCTTGTACTG
ATTCATTGCAAATTTTGTATATTGATTCGTTGGTCAAATAATTCAATTTCACGT
GTTTTTTATAATATTTGTAGATCATAGAGACTGACAGTGATGGTGGTTTGTTG
AAACTTACAAGAACCCAAGAGTGGCTAACAGGTGACAATTCTCCACCAATAA
ACAAGAAGGTGACTGCTAAGGTATGCTTTAGAGTAGTTTTCATTTTACATCCC
AACTTTCATTTTCTTCTGTATGTTATTATCTTGTCAGGGAAATTGGATATGAAA
TTGATGTGTTACAATGCATATTTTATTTTGTCTAATGTTTTTCAAATTCACCTT
CTCTCTGTGGTAAATAAATAATAACCCAGTCATCCAATAATGTTGGAAACTTC
ATTTCATATATCAACAGGCATTACAGGACAGCAGTGCAAGACGCATGAAACT
GAACATGCTCAAATATGAATCTGTACAATACCTCTCTTGACCTCGTTATTGTT
CTTTCTTTGCTCAAGTTTAGTGACATCAGTGTTTTCTTACCTCAAGGCATGTAG
GTTTTGATTAATTACAGTTGTTCAGATGTTGTGTAAGGCTGTTGGCTGCTTATT
CTGTCATTTTTATTCTTAATTGTAGCTCAAGAGGGAATTACTGCTTCTATCTGT
GGGTATTGGACTGGCTTGTAGTGGATATTGCTTGGTTATTTTTTCCGTACAGG

TAAACTTTACATTTGCATGCTGTGTCTCAAAAAATAATAAAAAAGTCTTATCA
TTTTGCTATTATAATTCAGAACCAGATCATTTTTGTGCTTCTTACCACTCAATT
ATGTTCTCTTTCTTCATGATAAGTCTAACTTTGGATGGATGCCAATGTCTTACC
ATAATGCTTTAACTTCAAACACTACCCCATGGTGTAAGTTTCAAATTATTTCC
TTCACTGATGTGTGCCATTCTCCTAAATGTGTAGGCTGCTATAAGTTATGCGA
TTGGAGTCCTTTTCAGGTTTGTTATTGCATTGTATTATATTGATACTGTCATAT
TGAATTATTTGTTAAATGAGCTTAAGGGCCAGTAGTAAGACAGTTAGAGCCT
AAATTGATATTGTTCATCATTGTTTTTTTTGTGTGTGAGAGAAAAGAAAAGAT
TAAACTGTTCTTAAAATTTGAGATTCAGACAATAGGATAAAAAATGTAACTA
TCCACACATTCTAATTTAATCTTTTTTTTTTTAATCTTAAGAATCGAAGATAGAT
ATCAAAGAGTTTATAACAACTCACGCATTCTAATTTAATTTAACAATAATAGG
TGATGAATTAATTGAATAGGTTGAAACCTGGCAAGACATATTTGCCGGTGGC
ATCAATGACAGTGACATCATCTCCAACCTGGGAATGGAAATGAATCATAAGC
AGAAAGTAACAAAAAGCGCAAGGTGTTAGAAAGCAAATTAAGCACTGCTAC
TGCTAGGGGGTTTCAATTCATCAAATACCCTGAAGTTAGGATTAACTGCAAC
AATGATGCCATCTTCCACGTAAACATCAGCCATCTGTTGGTGGTGACCATTCA
CAACCGTGCCTCCCTTGATCAACAACTTGGATGATGGAATTTCAGTCCCAGCA
TCACAAAACTGACTCACTCGAAAAGGACAAACATGAATTAAACATAGAACTA
GTGGAAATGAAAATCTCAGCTCAGTACTAATGATCATAGCTACTTAAAACAG
TTAAGAATCGAAGCTCTAACTCTTGGAAAACGCAGAACGGGGTAAGTAAACT
AGGTGAGCATAGGTCAACACATAAGAGAATTATTCCCTGCATTCAGTTTTTAT
AAAACTTGCAGAAGTATTAATGCAACTCCCCCTCCCTTTGGTAGTCACTAAAA
GAACAAGCGCACTTGAACTTCTATGGTTGATGTAAGGGTACGTGTCTTTGATT
TTCCCCCATGCTTGTTTGCAAGTGTAAACATGCTCAATTTCACTCTTTTCAATT
CTTCCTACAATTGCTTATTCATTTGCTTACTGTTTCAAAATTTAAAATAAATAC
TGTACAGTCATAAATAATAGTATATATGGCCTAGTAGTATATTTTGATTTTGT
AGTATCTCTAATTTACATGGAAACCGCCTTTTTTAAAAATTGTCTTTGCAAAA
ATTCCCTGCTAGAACATTTATCCAGTGGTATTCTTGAAAAAAAAATATATGTAG
ATTTATTGTTTACATTATGTATTCTGTCTGAAGCTTGTAAAGAGCTTTATGTTC
GTTTATGCCTTTCCCATTTTTTTTCTCTGGAAGGGGTGAAATATAGGATATTCT
ACTTTTTCAGTTTTTAGGTTCTAATAAGGCATAATTAAGTGGTAGACTCAGCT
TTAAAATTTTGTTCTTTCCTTTATATGGAGAATTATGTTTCAGATTAATGTGAC
TTGTAATAAAACCTTTTGAGCAAAACTGACTTGTTTTTATAAATTTCAGATGG
ATGTGCGGACTAATTCTCTCGTCAGGCCATCAGGGACAATTGACAAAGATAA
GGAAAAGCTACGGATTGCCAACAATGGTGTCGTTCAGAGTGAAGAACAAACC
TTACCTATTGGAGGTGATGGTTGGGAAAAGTCAAAAATGAAGAAGAAGCGTT
CCTGTATCAAACTAGATGTTTCTCCCAGTACAACATTGACTAAACCTGTTAAC
ACCTTCCAAGAAACTAAACAGGGAATGCAACAAAGACTTGCTACCGATTCGC
GATTCTTTCAGGTATGAATGTGTTATTTAATTATTTTTTTTTTGTTCTCATATA
ATATTTCTTATTTTAGGCTAAAATAATATTTTATGGGAAGATTAGAAAGTAGG
CACATTTTTTGTGGGAGGATTTAGGATAAGAAAATCCTCCCTTAAAATGAGG
AAAAAAGAGTCAAAATGTCCAAAAAAAAATGTTAGGCTTTTCAATCTTTATG
CACATCAACAAAAGAAATTCCTTTGAAATAACCACGAGCATATCCCTAGAGA
AAATCCATACATGAATTTTGGGGCAAAGAGACATTCTTGAAAATGCAATCAT
TCCAATTCCTCTCTAATAAACTGCCTGATAACTGTATAAATTGCTTAGTTCCA
TAGAATTGTAGCCTCCTGTTTGCTGCCAAATGTTACAATTGGAGAATGAAAAT

AAATTATAGGCAGTAACTGTTTGGAGTTCTCTCTAGAGTGTTAATTGTGAAAG
GATTTTCTTCAAAGAAAAACAATACAGTTGTGAAGGATTTTGCTGATATTAAG
GGTCATGAATTTAAAATCTTTGATGGATGATATCATGTAACAATTTATTTATT
TATTTGCTCTATCCAATTCTGATTTATTTTCTTGCTTAAACCTAGCCGATAAAT
ATAATAATTTCATGTATTTTTTTTAGGTCAGTAGTTTGTAATGGAACTATTGG
AGTTGGAAAATCAGATGGTATCTCTCAACAAACTGGGTTGGGCATACGAGCT
TCTACCCCTAGAAACAACCAAGATAATAATTCCCTTGTCAATGATAGGAGGG
GTCGTCCTGTTAGTTCAGACAAGGAAAGGGTGAACTTCAGAGTTGTAAACAA
GTAAATACAAGCTGTGGAACCCCTTTTCTTTATATGATTGTGCGTTTGGGAAA
AAGAATTGTCCTTCACTAAAATGTTTTTGCATGTTGCTTTCTCATTTTCTCCTT
ATTTTCGTCTTGTTTTGGTAGAATTGTTCAATTGTTTGCTTTCTGTTTGCTATG
GAGCCTTGCGATTATAGTTCATATATCTCTTCATCTTTGTGATATTTTTTCTGG
TATTGATGATTCATTCAGTATCTGTGTTATGTTCTATGACCAGGGCAACTGCA
CGTGATGAATTTAATTCATCTAGCCCTACCTCAAGTGCTAAAATAAACACTGC
TATCTGTGTTATGATATTTTTTATTAGTTCTATTGCATGCAATCAAGTTAATGG
TATACGTCACTATTATTACTGAGAAGTGGTTAAATGTCAAGTAGATCAAGCAT
GCAATATATAATTTTGGTTTAGATATTTATTTGGTGTTATAGATATGATCTAG
AAGATAAAATACAGCACAGCCATTAACTGTTGTAGCTTAATATGACATTGAC
ACTGATCGATTTCTGTATGTATCCAATGTCATTTGAAAACTGTATTTGCTAGC
TTCCATTACCTCTTGTTTAGGTTAAACTAAACCCAGAATCTAAATTGAAAATG
CTCACTGTAATTGGCTTGTATCTGGGAATATGGGTGGGGTGGGGTGTGGAAT
AAACTTCTCCATAAGCACTTCTTGGGAAAAAATGAGAAGGTAAAATGCATTG
AGCTTCTCCATAAGCTAAAAAATCAGCTTATACACCTTATCTTTTGGAGAAGA
TAAACGAAAAGAGCTTTTATAAAAATTAAGTGCATAAGTTGATTTTAGCTTAT
GTGCAAAATTCAATTCATTATATCTTCTTATTTTGGTAGTGAAGTTTATCAAA
ACGAGGTCAATGAATACAACTTTTTTGGCTTTAGTAATTTCGAGCAACAAATA
GCATTGATGTGACCTTGATTTGACTATTTCAGTGTTAGAACTTTTTTGAAAAC
TACAAATGTTTATTATGTTCTGCAAATTCTATATTATCTTTCTATTAGAAAAGT
CTTTGTTATTTAGAGATCTTGCATGCTAATCTTTATTATTAAATATTAATTTCT
ATTTACTAAGTTTTGAGCCTAGATATATGTTTATTTAGAGATCTGAAGCTATA
TACTATTGCCTGCATTTACTTATGTTTATTATTGGCATAACCTGTTGGTCTAAA
TTTTAGTTTCTAGTGATGCATTACTGTTTTATGCAGGTCTTTTCTGACTTGGAG
CGTTCAGTGTGTAGAATGGTCCATCGACAGGTTTGATTCTGATAAATTGCTTT
TAATGTTTTCTACTGTTGGTTTGAGAGATTATATTTACATTTTATTTTAGTAAC
TTGAGTATCGTATTCCGATTATGGCAGAACTTGATCAAGTTCAAGTGTATGAT
GAATTCCTTTTTGTTTAGCATTGGGATCATGGAACAAGTTTCCTTGATTTCATT
TAAAATATTTTGAAGATTTAAATCAGTTCATTTGATTCTGTAATATTAAGATT
TGTAACTTAATTACAATTTTGATTGTACATACCTTTCTCTGTTCCCATGGAGCT
TATTTTTTAATAAATAGACCTTTTGGATTTGTATTTAGTTTTTGTCTTACTACG
GATGCTTCTTTTCTTAACTTGGGGGATTCGTTTTTTAATTTTTTTGTAGGTTGC
CACAATTGCTTGGTTGGAAGCTGATTCTGTTTGTGGCAGCCACTCTATATGAC
TTCACATTTTCTTATTACCTTTTAATCTATAATATCATGTATTTATGCAAATGA
ACTGCATATACCTGTGTAATGAACTGCTTGCAAATGAAGTGTGAGAGGTGAA
TGCTTTTATGAAAATAGATTGATTGATGAATTTCTGTTACCATTCAGTGAAAT
CTGTCAAAGTATCATGTAGTTATGCTCACAATTTTTTTAAATGTATTTTGATTT
TTAAACAATTTATGTAAGTAATCTTTCCTTGCTGTTGTTGAGAAGAATAAAAA

ATTGTTTGTTCTAGCATTTTAGAACTAAGTGATCTTTATTTTATTTTTATCTGA
TAAACACTCGGTGATCCTTATATCAACTTTTTATCAATGTCCTTTGTTTGTTCA
GGGATTTATTGCATCCGCCCTTAATTATGGACTCATTACATGGTGACGAAGGT
TTCAGCATTGCTTTATGAAAGTTTTGTTGTCAAAGTTTGTAATTTGTGGACAA
GTTACAACTGAAGTTAGCAATGTTAGATATTCCTATGTAATTTTAAATTCTTG
CAAAACTATATACTATCATTTAATTATGTAAATTCTTTTATTATGTGATTAAAC
AATTTTATAATTCTTTAAAATGATGTAAATTCTTTCATTAATTATGTAAATTCT
TTTATTATGTAAATTCTTTTGCTTCAAATCAATGTATAGAATAATTGAAACAC
TACCTAAAAAACTAGACCTGCACCCAAAAAATTAAAAAAAAATATATATATT
CTAAGACGGTTGTCGGAAACCGTCTTAGTATGTTTCACTTTCTAAGACGTTTG
TATGAATAACCGCCTTAGAAACTTTTACAAACAAAAACATACTAAGACTGTT
TCACCTGAACCGTCTTAGTATGTTTGACATTCTAAGACGGTTTTCGTAACAAT
CGTCTTAGAAAGTCTCACAAACAAATGACATACTAAGACGGTTTTCGTAACA
ATCGCCTTAGAAAGTCTCACAAACAAATGACATACTAAGACGGTTGTTGAAA
AATCGTCTTAGTATGTCTCACTTTCTATGGCGGTTTTTACGAAAACCGTCTTA
AAAACCTTCAAAAAAAGGACATACTAAGATGGTTGCAGGAGAAAACCGTCTT
AGTATGTCTAACTTTCTAAGACGGTTTTTGCAATAACCGTCTTAGTAAGTCTC
ACTTTCTAAGACGGTTGTAGAAAAACCGTCCTAGTAAGTCTCACTTTTTTAAGA
CGGTTTTTGTAATAACCGTCGTAGAAAGTTTGGTTTATTATAACGGTTAGTAG
ATGATAACCGCCTTGAATTAATAATAAATTTTAAGACGGTTATTCTTAAAACC
GTCTTAACAACTCCAATCTTTAAAGACGGTTTGAAAACCGTCGTTGTAGAGGT
GGGCACATTTTACGACGCTGCGTGCTATGACGGTTCAAAACCGTCGTAAAAT
GACTTGCAGAACCGACTTAAAAAGCTTTATTTGTAGTAGTG
