# Supplementary material for: A putative autonomous 20.5 kb-CACTA transposon insertion in an F3'H allele identifies a new CACTA transposon subfamily in Glycine max
Source: BMC Plant Biol. 2008 Dec 2;8:124. doi: 10.1186/1471-2229-8-124 (PMC2613891; doi:10.1186/1471-2229-8-124)
Supplement: Additional file 10 — Alignment of sequences of 3 contigs from 71B1 BAC clone in the 7× draft sequence assembly (JGI). [file 1471-2229-8-124-S10.pdf]

Additional file 10. Alignment of sequences of 3 contigs from 71B1 BAC clone in the 7x draft sequence assembly (JGI)

Query_ID	Hit_ID	Alignment_	HSP	EValue	Identity	Query_Le	Sub_Len	Quer	Target	Query_Sta	Query_En	Sub_Start	Sub_End
ZT_finalcontig.2(40518	scaffold_83	15419	28084.55	0	95	40518	3617036	C	D	1	15395	2365666	2381076
ZT_finalcontig.2(40518	scaffold_83	3874	7188.52	0	95	40518	3617036	C	D	15399	19272	2380665	2384531
ZT_finalcontig.2(40518	scaffold_83	15070	26468.93	0	89	40518	3617036	C	D	18026	33095	2392007	2407075
ZT_finalcontig.2(40518	scaffold_83	6502	12025.47	0	93	40518	3617036	C	D	33122	39622	2407075	2413576
ZT_finalcontig.1(23207	scaffold_83	2900	5584.79	0	97	23207	3617036	C	D	768	3667	2413623	2416522
ZT_finalcontig.1(23207	scaffold_83	7632	12766.87	0	85	23207	3617036	C	D	3664	11294	2416510	2424137
ZT_finalcontig.1(23207	scaffold_83	2010	3243.63	0	85	23207	3617036	C	D	11294	13302	2424151	2426157
ZT_finalcontig.1(23207	scaffold_83	224	436.61	3.10E-121	99	23207	3617036	C	D	13327	13550	2426158	2426381
ZT_finalcontig.1(23207	scaffold_83	922	1217.66	0	77	23207	3617036	C	D	13716	14634	2426530	2427449
ZT_finalcontig.1(23207	scaffold_83	3671	6129.94	0	87	23207	3617036	C	D	14635	18303	2426906	2430569
ZT_finalcontig.3(9467nt)	scaffold_83	3599	7000.2	0	99	9467	3617036	C	D	5825	9423	2383285	2386883
ZT_finalcontig.3(9467nt)	scaffold_83	7021	13686.69	0	98	9467	3617036	C	D	51	7071	2386233	2393253
